# A pairwise and network meta-analysis of anti-inflammatory strategies after myocardial infarction: the TITIAN study

**DOI:** 10.1093/ehjcvp/pvae100

**Published:** 2025-01-03

**Authors:** Claudio Laudani, Giovanni Occhipinti, Antonio Greco, Daniele Giacoppo, Marco Spagnolo, Davide Capodanno

**Affiliations:** Division of Cardiology, Azienda Ospedaliero-Universitaria Policlinico “Rodolico—San Marco”, University of Catania, Via S. Sofia 76, 95125 Catania, Italy; Division of Cardiology, Azienda Ospedaliero-Universitaria Policlinico “Rodolico—San Marco”, University of Catania, Via S. Sofia 76, 95125 Catania, Italy; Institut Clinic Cardiovascular, Hospital Clínic de Barcelona, University of Barcelona, Carrer de Villarroel, 170, L'Eixample, 08036 Barcelona, Spain; Division of Cardiology, Azienda Ospedaliero-Universitaria Policlinico “Rodolico—San Marco”, University of Catania, Via S. Sofia 76, 95125 Catania, Italy; Division of Cardiology, Azienda Ospedaliero-Universitaria Policlinico “Rodolico—San Marco”, University of Catania, Via S. Sofia 76, 95125 Catania, Italy; Division of Cardiology, Azienda Ospedaliero-Universitaria Policlinico “Rodolico—San Marco”, University of Catania, Via S. Sofia 76, 95125 Catania, Italy; Division of Cardiology, Azienda Ospedaliero-Universitaria Policlinico “Rodolico—San Marco”, University of Catania, Via S. Sofia 76, 95125 Catania, Italy

**Keywords:** Coronary artery disease, Myocardial infarction, Acute coronary syndrome, Anti-inflammatory therapy, Cardiovascular pharmacotherapy

## Abstract

**Background and aims:**

Multiple anti-inflammatory drugs have been tested for secondary prevention after myocardial infarction (MI), giving mixed results and questioning the efficacy of anti-inflammatory therapy. No head-to-head comparisons between anti-inflammatory drugs have been performed. This study aimed to compare the efficacy and safety of anti-inflammatory drugs for secondary prevention after MI and the relative merits of specific drugs and administration strategies.

**Methods and results:**

Randomized trials of anti-inflammatory therapy for secondary prevention after MI were identified. Primary efficacy and safety endpoints were trial-defined major adverse cardiovascular events (MACEs) and serious adverse events. Secondary endpoints included all-cause death, individual MACE components, serious infection, cancer, and gastrointestinal adverse events. Pairwise meta-analyses were conducted with interaction analyses for drug type and timing of administration, in addition to network meta-analyses. Multiple sensitivity and meta-regression analyses were conducted to explore potential heterogeneity sources. Twenty-eight studies, involving 44 406 patients with a mean follow-up of 11 months, were included. Anti-inflammatory therapy reduced the incidence of MACEs [incidence rate ratio (IRR): 0.92; 95% confidence interval (CI): 0.86–0.98] compared to control, without increasing serious adverse events. However, it was associated with a higher incidence of gastrointestinal adverse events (IRR: 1.21; 95% CI: 1.07–1.36). No significant interaction was observed between the effects of anti-inflammatory therapy on MACE and the timing of administration.

**Conclusion:**

In secondary prevention for MI, anti-inflammatory therapy significantly reduces MACE without increasing serious adverse events, but it is associated with an increased risk of gastrointestinal adverse events.

## Graphical Abstract

**Graphical Abstract:**
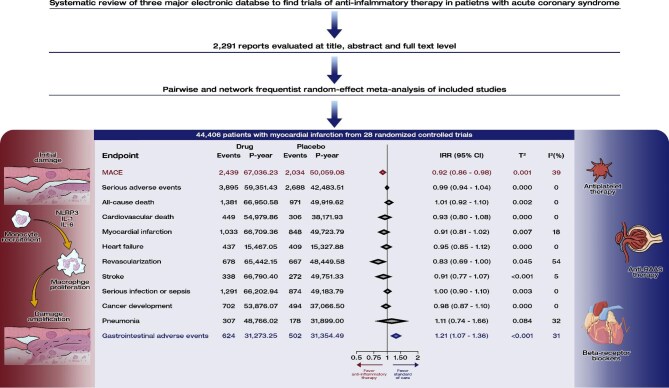
**Treatment effects of anti-inflammatory therapy**. Significant results are highlighted in red when favouring anti-inflammatory therapy or blue when favouring control. The box on the left side highlights how inflammation may increase tissue damage, while the box on the right side highlights current pillars of post-infarction pharmacotherapy. Abbreviations: IRR, incidence rate ratio; P-year, patient-year.

## Introduction

Atherosclerosis is the biological substrate of ischaemic heart disease—the leading global cause of mortality—and myocardial infarction (MI).^1^ The paradigm of atherosclerosis pathophysiology has shifted over time from being regarded as a ‘lipid storage disease’ to the contemporary hypothesis that cardiovascular risk factors act synergistically as inflammatory triggers, culminating in arterial plaque development.^2^

A pivotal role in both the progression of atherosclerosis and post-MI cardiac remodelling has been attributed to inflammation. Initial complement activation and local interleukin (IL) production are essential to allow for early macrophage recruitment and tissue healing during ischaemia,^3^ but an excess of cytokines induced by nucleotide-binding oligomerization domain-like receptor protein 3 (NLRP3) stimulation may hamper the natural transition towards a pro-fibrotic environment, thereby hindering positive ventricular remodelling over the long-term.^4^ Additionally, persistent low-grade systemic inflammation following the index event continues to influence the myocardium and arterial walls, intensifying plaque progression and adverse remodelling.^5^

Several anti-inflammatory drugs have been tested in randomized trials for secondary cardiovascular prevention in patients with a prior MI, targeting different inflammatory pathways and administered at various timepoints post-event. These trials have produced mixed results, raising questions about the overall efficacy of anti-inflammatory therapy in this context. Colchicine has the most robust evidence supporting its use, but its effectiveness has recently been questioned in patients with acute MI following the neutral results of the CLEAR SYNERGY (OASIS 9) trial.^6^ This contrasts with previous studies like COLCOT and LoDoCo2, which showed benefits of colchicine across the spectrum of coronary artery disease.^7,8^ Additionally, the potential benefits of anti-inflammatory therapy must be weighed against its associated risks, including increased susceptibility to infections and cancer, which could lead to higher mortality. The timing of drug administration is another important factor. Early administration during an acute MI may prevent initial complement activation, cytokine release, and excessive macrophage recruitment, promoting positive remodelling. In contrast, later administration might slow plaque progression and prevent ischaemic recurrences.^5^

No head-to-head trials have directly compared anti-inflammatory drugs, and previous meta-analyses have either focused on individual drugs in pairwise comparisons^9^ or assessed only a limited number of treatments without incorporating newer data.^10,11^ Against this background, the objective of this systematic review and meta-analysis was to assess the efficacy and safety of anti-inflammatory therapy for secondary prevention following MI, as well as to compare the relative merits of different drugs and administration timing.

## Methods

The TITIAN (Targeting InflammaTIon After myocardial iNfarction) meta-analysis has been conducted in adherence to the 2020 PRISMA recommendations (Supplementary material online, *Tables S1* and *S2*) and Cochrane Collaboration.^12,13^ The study protocol was registered in PROSPERO (CRD42024508874, Supplementary material). Approval from the ethics committee was waived due to the nature of this study.

### Eligibility criteria

Studies were eligible for inclusion if they: (i) included at least 80% of patients with acute coronary syndrome or prior MI and (ii) had random allocation to any anti-inflammatory drug or standard of care. No restrictions were imposed based on patients’ age, sex, ethnicity, or language of publication.

### Search, data extraction, and qualitative assessment

Details of search strategies, data extraction, and qualitative assessment are reported in Supplementary material online, *Table S3* and the Supplementary methods. In brief, the MEDLINE, Cochrane, and Web of Science databases were screened from inception to January 2024, and clinical, procedural and outcome data were extracted. For trials comparing different doses of the same drug, following the recommendations of the Cochrane collaboration, the investigational arms were lumped to prevent double-counting of events in the control group that would spuriously increase the degree of precision.^14^ The Cochrane's Risk of Bias 2 tool and grading of recommendations assessment, development and evaluation (GRADE) approach were used for qualitative assessment of the studies,^15^ while visual appraisal of funnel plots and Egger's test were used to assess the chance of publication bias.

### Study endpoints

The primary efficacy endpoint was the composite of trial-defined major adverse cardiovascular events (MACEs), with priority for definitions aligned with the composite of cardiovascular death, MI, or stroke. The primary safety endpoint was the composite of trial-defined serious adverse events. Secondary endpoints included all-cause death, cardiovascular death, MI, revascularization, heart failure, stroke, serious infections or sepsis, pneumonia, cancer, and gastrointestinal adverse events.

### Statistical analysis

Pairwise meta-analyses were performed with frequentist random-effect models to compute incidence rate ratios (IRRs) and 95% confidence intervals (CIs) of the treatment effect of anti-inflammatory therapy compared to placebo or no anti-inflammatory therapy (i.e. control). Interaction analyses were conducted by drug, clinical presentation [i.e. acute MI (within 30 days before randomization) or prior MI] and timing of drug administration [i.e. immediate (within 2 h from index event), early (between 2 h and 30 days), or late (more than 30 days after the index event)] using Q tests for between-group heterogeneity.^16^

Statistical heterogeneity was assessed using *τ*^2^, *I*^2^ and prediction intervals, and was categorized as low, moderate, or high for *I*^2^ levels of <25%, 25% to 50%, and >50%, respectively. Endpoints with moderate or high heterogeneity were further scrutinized to identify potential outliers.

Exploratory indirect comparisons of anti-inflammatory therapies were performed by means of frequentist random-effect network meta-analyses using control as a common reference treatment. Treatments were ranked based on the surface under the cumulative ranking values.

Sensitivity analyses were also performed, being restricted to trials using consistent definitions of MACE, phase 3 trials, trials with at least 6 months of follow-up, and trials that only enrolled patients with previous MI, presenting in the acute phase, or presenting with ST-segment elevation MI. Additionally, meta-regressions were conducted to explore the influence of the year of publication, age, sex, diabetes, hypertension, statins, and antiplatelet therapy on estimates of the treatment effect for outcomes reported in at least 10 trials, as recommended by the Cochrane collaboration. Lastly, leave-one-out analyses were conducted to evaluate the influence of each trial on treatment effects.

More details on the study methodology is available in the Supplementary methods. All the analyses were performed with R 4.3.1 (The R Foundation for Statistical Computing—Vienna, Austria).

## Results

### Study selection and baseline characteristics

The study selection process is summarized in Supplementary material online, *Figure S1*. After the screening phase, 28 studies were selected, appraised for quality and included in the quantitative synthesis of treatment effects.^6–8,17–41^ The treatment network displayed a star-shaped configuration (Supplementary material online, *Figure S2*), with all treatments being compared to control (i.e. placebo or no anti-inflammatory drug). Key design characteristics of these studies are summarized in *Table 1* and Supplementary material online, *Table S4*. Overall, there were ten trials of colchicine, four of cyclosporine, three of anakinra, three of pexelizumab, three of tocilizumab, two of methotrexate, one of canakinumab, one of inclacumab, and one of everolimus. Sixteen trials evaluated immediate administration of anti-inflammatory therapy, while nine and three trials investigated early and late administration timing, respectively. The mean follow-up across trials was 11 ± 15 months.

**Table 1:** Main characteristics of included studies

**Study, year**	**Design**	**Blinding**	**Sample size**	**Presentation**	**Experimental drug**	**Follow-up time**
**COMPLY, 2003**	Phase 2, 1:1:1 randomization	Double-blind	920	STEMI	Pexelizumab	3 months
**COMMA, 2003**	Phase 2, 1:1:1 randomization	Double-blind	814	STEMI	Pexelizumab	3 months
**APEX AMI, 2007**	Phase 3, 1:1 randomization	Double-blind	5745	STEMI	Pexelizumab	3 months
**Piot et al., 2008**	Pilot, 1:1 randomization	Single-blind	58	STEMI	Cyclosporine	In-hospital
**Ghaffari et al., 2012**	Phase 2, 1:1 randomization	Double-blind	101	STEMI	Cyclosporine	6 months
**VCU-ART2, 2013**	Pilot, 1:1 randomization	Double-blind	30	STEMI	Anakinra	12 weeks
**SELECT-ACS, 2013**	Phase 2, 1:1:1 randomization	Double-blind	530	STEMI	Inclacumab	4 months
**MRC-ILA, 2014**	Phase 2, 1:1 randomization	Double-blind	182	NSTEMI	Anakinra	1 year
**CIRCUS, 2015**	Phase 3, 1:1 randomization	Double-blind	791	STEMI	Cyclosporine	1 year
**CYCLE, 2015**	Phase 2, 1:1 randomization	Open label	410	STEMI	Cyclosporine	6 months
**Deftereos et al., 2015**	Pilot, 1:1 randomization	Double-blind	151	STEMI	Colchicine	In-hospital
**COLIN, 2016**	Phase 2, 1:1 randomization	Open label	45	STEMI	Colchicine	1 month
**Kleveland et al., 2016**	Phase 2, 1:1 randomization	Double-blind	118	NSTEMI	Tocilizumab	6 months
**CANTOS, 2017**	Phase 3, 1.5:1:1:1 randomization	Double-blind	10 061	Previous MI	Canakinumab	3.7 years*
**STAT-MI, 2017**	Pilot, 1:1 randomization	Double-blind	27	Acute MI	Tocilizumab	1 month
**TETHYS, 2017**	Phase 2, 1:1 randomization	Double-blind	81	STEMI	Methotrexate	3 months
**CIRT, 2018**	Phase 3, 1:1 randomization	Double-blind	4786	Previous MI	Methotrexate	2.3 years*
**COLCOT, 2019**	Phase 3, 1:1 randomization	Double-blind	4745	Acute MI	Colchicine	22.6 months*
**VCUART3, 2019**	Phase 2, 1:1:1 randomization	Double-blind	99	STEMI	Anakinra	12 months
**LoDoCo-MI, 2019**	Pilot, 1:1 randomization	Double-blind	222	Acute MI	Colchicine	1 month
**LoDoCo2, 2020**	Phase 3, 1:1 randomization	Double-blind	5522	Previous ACS	Colchicine	28.6 months*
**Australian COPS, 2020**	Phase 3, 1:1 randomization	Double-blind	795	ACS	Colchicine	400 days
**ASSAIL -MI, 2021**	Phase 2, 1:1 randomization	Double-blind	199	STEMI	Tocilizumab	6 months
**Akrami et al., 2021**	Phase 3, 1:1 randomization	Double-blind	249	Acute MI	Colchicine	6 months
**CLEVER-ACS, 2022**	Phase 2, 1:1 randomization	Double-blind	150	STEMI	Everolimus	30 days
**PodCAST-PCI, 2022**	Phase 3, 1:1 randomization	Double-blind	321	STEMI	Colchicine	1 year
**COVERT-MI, 2023**	Phase 2, 1:1 randomization	Double-blind	192	STEMI	Colchicine	1 year
**CLEAR SINERGY (OASIS 9), 2024**	Phase 3, 1:1 randomization	Double-blind	7062	Acute MI	Colchicine	3 years*

*Median time.

Abbreviations: MI, myocardial infarction; NSTE-ACS, non-ST-segment elevation acute coronary syndrome; NSTEMI, non-ST-segment elevated myocardial infarction; and STEMI, ST-segment elevation myocardial infarction.

A total of 44 406 patients were included (24 265 allocated to anti-inflammatory therapy and 20 141 patients allocated to control). Of them, 43% presented with acute MI while 57% had a prior MI. The mean age was 61.9 ± 2.9 years, 20.6% were women and 24.4% had diabetes mellitus (*Table 2* and Supplementary material online, *Table S5*).

**Table 2:** Baseline characteristics of included studies’ population

**Study, year**	**Mean age**	**Female (%)**	**Diabetes (%)**	**Hypertension (%)**	**Statins (%)**	**Antiplatelet therapy (%)**	**Hs-CRP levels (mg/L)**
**COMPLY, 2003**	60.3	9.9	5.1	18.5	20.2	27.5	NR
**COMMA, 2003**	60.3	9.0	7.1	22.7	27.9	35.1	NR
**APEX AMI, 2007**	61.0 (I) vs.	23.1	15.9	NR	91.0	94.4	NR
	61.0 (C)*						
**Piot et al., 2008**	57.5	20.7	13.8	48.3	NR	91.4	NR
**Ghaffari et al., 2012**	62.1	16.8	34.7	42.6	99.0	99.0	NR
**VCU-ART2, 2013**	58.7	26.7	20.0	60.0	NR	NR	7.0 (I) vs. 4.3 (C)
**SELECT-ACS, 2013**	61.1	21.1	22.7	NR	95.1	93.2	NR
**MRC-ILA, 2014**	61.4	28.6	12.6	33.0	95.0	95.0	5.4 (I) vs. 5.2 (C)
**CIRCUS, 2015**	59.9	18.0	12.7	37.3	NR	92.7	NR
**CYCLE, 2015**	62.9	20.2	14.2	55.4	89.0	99.3	NR
**Deftereos et al., 2015**	58.0	31.1	21.2	39.7	100.0	100.0	2.5 (I) vs. 2.4 (C)
**COLIN, 2016**	59.9	18.6	14.0	44.2	100.0	100.0	NR
**Kleveland et al., 2016**	60.0	12.0	17.9	36.8	90.6	99.1	4.2 (I) vs. 2.0 (C)^¶^
**CANTOS, 2017**	61.1	25.7	40.0	79.6	91.0	NR	4.1 (I) vs. 4.2 (C)
**STAT-MI, 2017**	70.0	14.3	64.3	89.3	100.0	100.0	NR
**TETHYS, 2017**	58.4	26.2	20.2	58.3	91.7	100.0	5.3 (I) vs. 4.5 (C)
**CIRT, 2018**	65.6 (I) vs.	18.8	33.7	90.3	85.9	NR	1.5 (I) vs. 1.5 (C)
	66.0 (C)*						
**COLCOT, 2019**	60.5	19.2	20.2	51.0	99.0	98.8	4.28
**VCUART3, 2019**	54.7	19.2	30.3	56.6	99.0	100.0	NR
**LoDoCo-MI, 2019**	61.0	23.2	21.9	47.3	98.3	99.6	6.9 (I) vs 7.7 (C)
**LoDoCo2, 2020**	65.8	15.3	18.2	50.9	93.9	99.7	NR
**Australian COPS, 2020**	59.9	20.5	19.0	50.3	98.9	98.6	NR
**ASSAIL -MI, 2021**	61.0	15.6	7.0	32.0	14.1	100.0	2.4 (I) vs. 2.9 (C)
**Akrami et al., 2021**	56.9	30.5	23.7	44.6	100.0	100.0	NR
**CLEVER-ACS, 2022**	61.9	14.7	9.3	40.7	86.7	92.7	NR
**PodCAST-PCI, 2022**	58.9	20.9	35.5	39.6	NR	NR	176.5 (I) vs. 244.5 (C)
**COVERT-MI, 2023**	59.9	19.8	13.0	30.7	82.8	NR	3.0 (I) vs. 3.0 (C)
**CLEAR SINERGY (OASIS 9), 2024**	60.7	20.4	18.5	45.8	96.7	96.8	NR

*Median.

¶ Median area under the curve.

Abbreviations: C, control and I, intervention.

The global and individual risks of bias of the studies included in the meta-analysis are shown in Supplementary material online, *Figures S3* and *S4*, respectively. One study^31^ was deemed at high risk of bias and five studies^20,21,32,35,39^ were fraught with concern due to uncertainty in the randomization process. Funnel plots and Egger's tests did not display overt signs of publication bias (Supplementary material online, *Figure S5*). The endpoints extracted from each trial and their definitions are reported in Supplementary material online, *Tables S6* and *S7*, respectively, while complete GRADE assessment of evidence is displayed in Supplementary material online, *Table S8*.

### Major adverse cardiovascular events

Patients receiving anti-inflammatory therapy had significantly lower trial-defined MACE compared to controls (IRR: 0.92; 95% CI: 0.86–0.98), with moderate heterogeneity (*τ*^2^ <0.001; *I*^2^: 39%; prediction interval: 0.84–1.01) (Graphical abstract, *Figure 1*). Three trials were responsible for most of such heterogeneity (Supplementary material online, *Figure S6*).^24,31,38^ Removal of these studies did not unduly influence the treatment estimate for MACE (IRR: 0.94; 95% CI: 0.88–0.99; no heterogeneity) (Supplementary material online, *Figure S7*).

**Figure 1:**
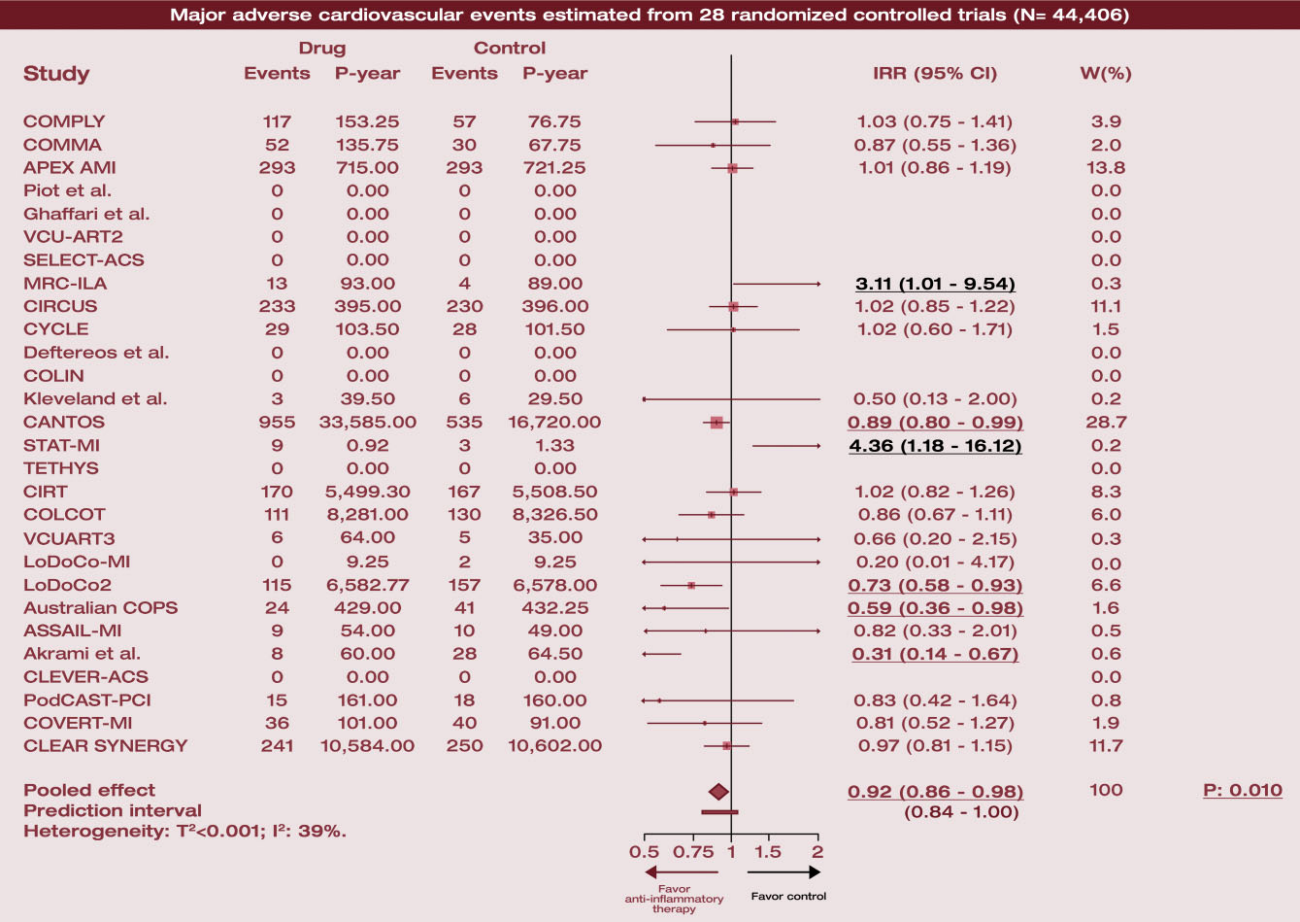
Forest plot for the primary efficacy endpoint. For each trial, relative treatment effect estimates and confidence intervals are displayed as square and horizontal bar, respectively, along with events and P-year and contribution to the analysis. The overall pooled effect is displayed at the bottom as a diamond shape, along with measures of heterogeneity. Abbreviations: N, number of patients; IRR, incidence rate ratio; *P, P*-value; P-year, patient-year; and W(%), percentage weight on pooled estimate.

No significant interactions by drug type (*P* = 0.253 for interaction), timing of administration (*P* = 0.113 for interaction) or clinical presentation (*P* = 0.170 for interaction; Supplementary material online, *Table S9*) were observed. The MACE findings were mostly driven by trials of colchicine (IRR: 0.78; 95% CI: 0.66–0.93) and canakinumab (IRR: 0.89; 95% CI: 0.80–0.99) (*Figure 2*).

**Figure 1:**
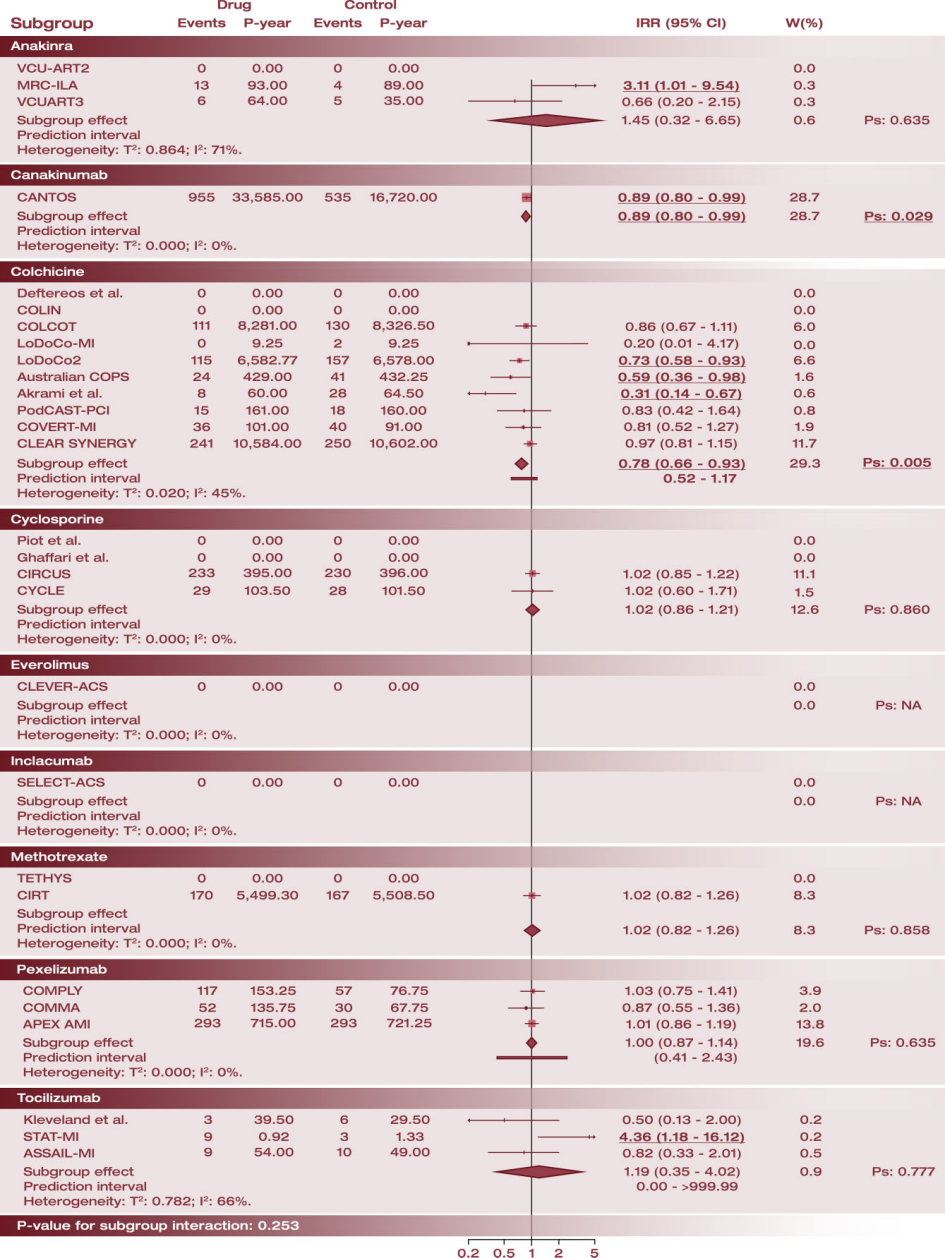
Interaction analysis for drug administered for the primary efficacy endpoint. For each subgroup, the pooled effect is displayed as a diamond shape, along with heterogeneity estimates and *P*-value for subgroup effect. Results of the between-subgroup interaction test is displayed at the bottom. Abbreviations: IRR, incidence rate ratio; Ps, *P*-value for subgroup effect; P-year, patient-year; and W(%), percentage weight on the overall estimation.

In exploratory indirect comparisons, no significant differences were apparent between drugs (Supplementary material online, *Table S10*), but colchicine had a 91.9% probability of ranking first for this endpoint (Supplementary material online, *Table S11*). The heterogeneity of the network meta-analysis was moderate (*τ*^2^:0.007; *I*^2^: 42%), and the exclusion of trials that mostly contributed to high heterogeneity resulted in similar findings with no heterogeneity (Supplementary material online, *Table S12*).

### Serious adverse events

**Figure 3:**
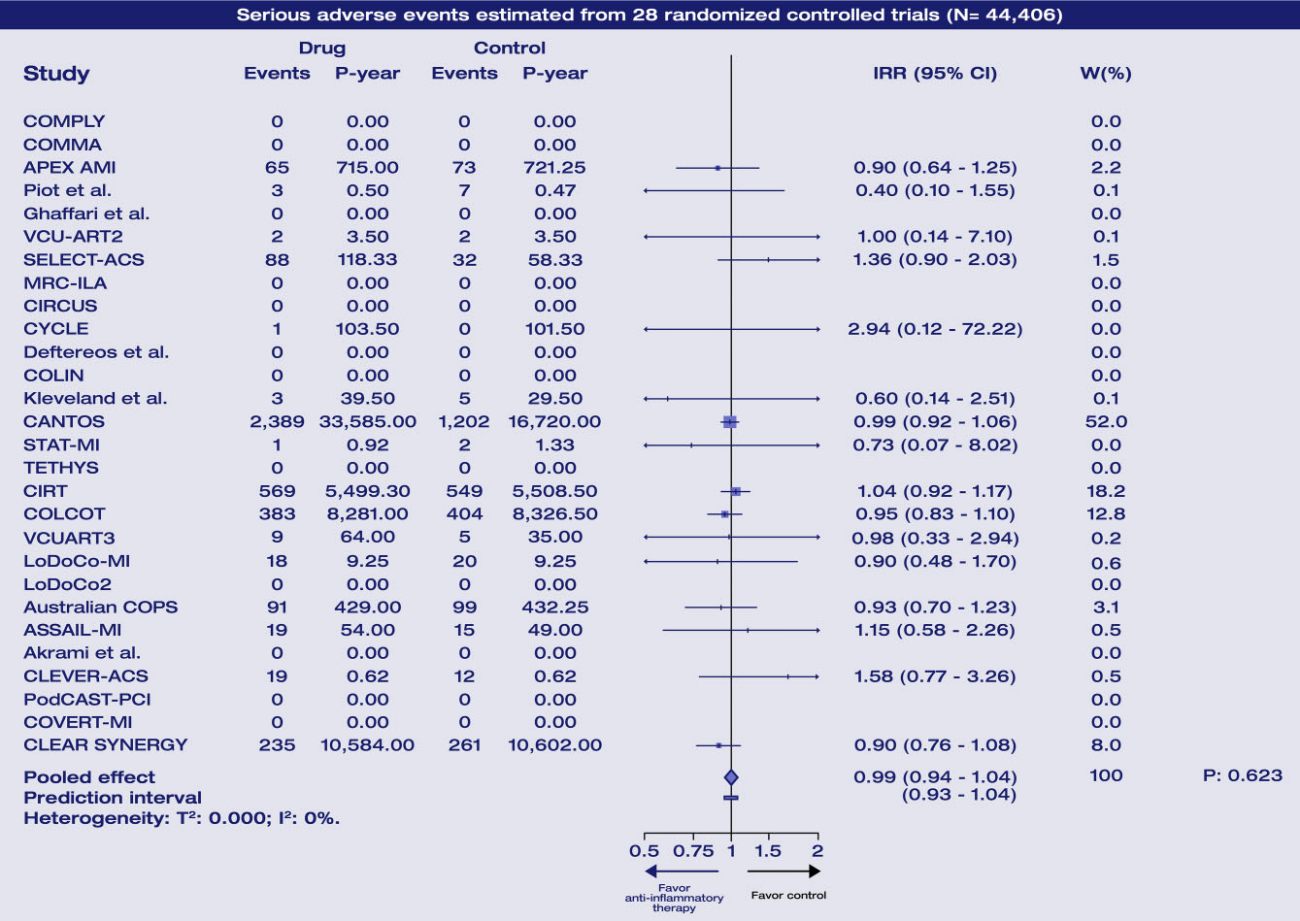
Forest plot for the primary safety endpoint. For each trial, relative treatment effect estimates and confidence intervals are displayed as square and horizontal bar, respectively, along with events and P-year and contribution on the analysis. The overall pooled effect is displayed at the bottom as a diamond shape, along with measures of heterogeneity. Abbreviations: N, number of patients; IRR, incidence rate ratio; P, *P*-value; P-year, patient-year; W(%), percentage weight on pooled estimate.

**Figure 4:**
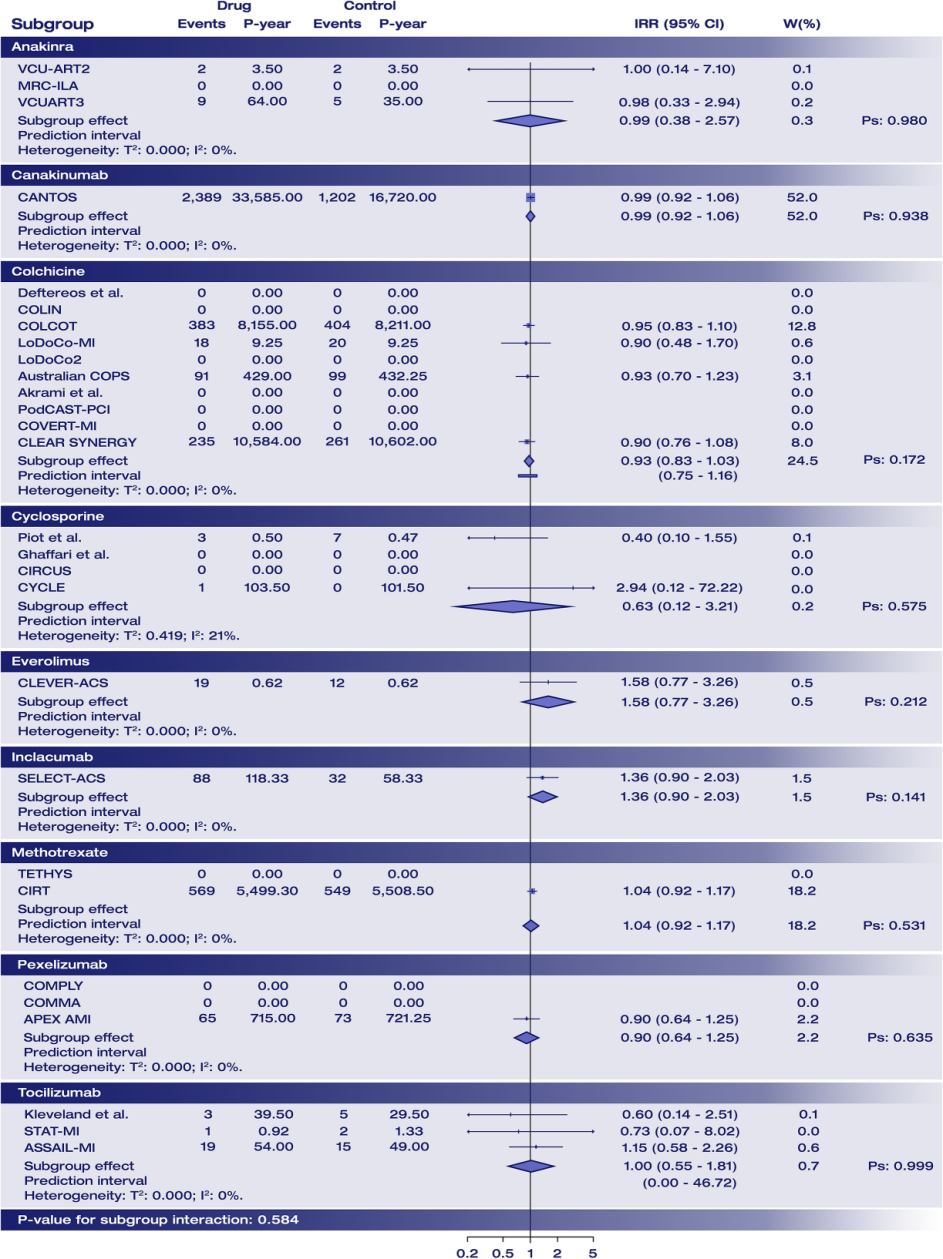
Interaction analysis for drug administered for the primary safety endpoint. For each subgroup, the pooled effect is displayed as a diamond shape, along with heterogeneity estimates and *P*-value for subgroup effect. Results of the between-subgroup interaction test is displayed at the bottom. Abbreviations: IRR, incidence rate ratio; Ps, *P*-value for subgroup effect; P-year, patient-year; W(%), percentage weight on the overall estimation.

Anti-inflammatory therapy did not increase the risk of serious adverse events (IRR: 0.99; 95% CI: 0.94–1.04; Graphical abstract, *Figure 3*), and heterogeneity was null (*τ*^2^:0.000; *I*^2^: 0%; prediction interval: 0.94–1.04). There was no significant interaction by drug type (*P* = 0.584 for interaction; *Figure 4*), clinical presentation (*P* = 0.549) or timing of administration (*P* = 0.558 for interaction) (Supplementary material online, *Table S9*).

In indirect comparisons, no significant differences were observed between treatments (Supplementary material online, *Table S10*), and no heterogeneity was detected (τ^2^ < 0.000; *I*^2^: 0%).

### Secondary endpoints

Patients on anti-inflammatory therapy had a higher risk of gastrointestinal adverse events compared to controls (IRR: 1.21; 95% CI: 1.07–1.36), with no difference in other secondary endpoints (Graphical abstract), including all-cause death.

Although no significant interaction by drug type was found in any secondary endpoint (Supplementary material online, *Figures S8*–*S17*), colchicine was found to reduce the risk of MI (IRR: 0.84; 95% CI: 72–0.99; *τ*^2^:0; *I*^2^: 0%), while canakinumab reduced the risk of MI (IRR: 0.86; 95% CI: 74–0.99; *τ*^2^:0; *I*^2^: 0%) and revascularization compared to controls (IRR: 0.64; 95% CI: 0.49–0.86; *τ*^2^:0; *I*^2^: 0%) (Supplementary material online, *Figures S10* and *S12*). Heterogeneity was low for all the secondary endpoints, with the exception of revascularization (*τ*^2^: 0.045; *I*^2^: 54%; prediction interval: 0.49–1.40), pneumonia (*τ*^2^: 0.084; *I*^2^: 32%; prediction interval: 0.41–2.97), and gastrointestinal events (*τ*^2^ <0.001; *I*^2^: 31%; prediction interval: 1.07–1.36).

Statistically significant interactions between anti-inflammatory therapy and the timing of drug administration and clinical presentation were observed for revascularization (Supplementary material online, *Table S9*). In particular, revascularization was significantly reduced in studies where the anti-inflammatory therapy was administered at a later time (IRR: 0.73; 95% CI: 0.62–0.86), and in patients with prior MI (IRR: 0.70; 95% CI: 0.60–0.82), while no effect was observed when the anti-inflammatory drugs were given earlier in time (*P* = 0.012 for interaction) or in patients with acute MI (*P* <0.001 for interaction).

The indirect comparison of anti-inflammatory drugs suggested that anakinra significantly increases the risk of MI compared to canakinumab, colchicine, cyclosporine, and tocilizumab (Supplementary  material online, *Table S10*). Low heterogeneity was noted in all the secondary endpoints except revascularization and pneumonia, which showed high heterogeneity (τ^2^: 0.090; *I*^2^: 59% for revascularization; τ^2^: 0.404; *I*^2^: 69% for pneumonia, respectively), and gastrointestinal adverse events, which showed moderate heterogeneity (τ^2^ < 0.001; *I*^2^: 46%). In the leave-one-out analyses, the LoDoCo2 trial had the highest impact on heterogeneity for revascularization and pneumonia, while the trial by Deftereos et al. had the highest impact on heterogeneity for gastrointestinal adverse events. Repeat analyses after removal of these trials highlighted the potential benefits of colchicine over anakinra, inclacumab, and pexelizumab for the endpoint of revascularization, despite a potential higher risk of pneumonia compared to canakinumab (Supplementary material online, *Table S12*).

### Sensitivity and meta-regression analyses

The results of the sensitivity analyses, which are detailed in Supplementary material online, *Tables S13*–*S25* and Supplementary material online, *Figures S18*–*S25*, were generally consistent with the main analysis, with no evidence of major influence of one trial over another and minor differences regarding the endpoint of revascularization, whose risk was reduced by anti-inflammatory therapy after excluding trials with greater impact on heterogeneity (Supplementary material online, *Figure S19*).

The meta-regression analyses did not show significant associations of specific variables with the efficacy of anti-inflammatory therapy for the primary efficacy and safety endpoints but suggested that the use of beta-blockers might mitigate the treatment effect for MI (Supplementary material online, *Table S26*).

## Discussion

The results of the TITIAN study can be summarized as follows (Graphical abstract): (i) anti-inflammatory therapy for secondary cardiovascular prevention after MI decreases the risk of MACE; (ii) although no excess of serious adverse events was associated with anti-inflammatory therapy, the potential benefits of these drugs should be weighed against the increased risk of gastrointestinal adverse events; and (iii) among anti-inflammatory drugs, colchicine had the highest probability of reducing MACE. Overall, our data highlight a significant class effect of anti-inflammatory therapy in reducing MACE in secondary prevention after MI, with colchicine currently playing a prominent role over other compounds.

The understanding of MI pathophysiology suggests that inflammation plays a pivotal role in both disease progression and myocardium remodelling.^42,43^ The number and type of cytokines produced during the initial phase appear to be of key importance in determining the type and extension of heart remodelling.^44^ Indeed, although the initial IL -1 and IL -6 burst is necessary to induce macrophage recruitment and tissue replacement, it may also lead to myocardial damage, which can be mitigated by anti-inflammatory therapy.^44^ In addition, residual inflammation after the index event appears to be the main driver of atherosclerosis progression and plaque destabilization, suggesting a role of anti-inflammatory therapy for long-term secondary prevention.^42,45^

Randomized trials investigating anti-inflammatory therapy after MI have yielded mixed results, particularly following the publication of the CLEAR SYNERGY (OASIS 9) trial, which raised doubts about the effectiveness of colchicine specifically, and even the broader strategy of targeting inflammation in this setting. Discrepancies in the overall risk-benefit profile of anti-inflammatory therapy have been attributed to factors such as drug-specific effects, differences in targeted pathways, and the timing of administration after the index event.^5^ To the best of our knowledge, TITIAN is the first study to investigate these interactions in the post-MI prevention setting, addressing limitations of previous meta-analyses, which were restricted to pairwise comparisons,^9,10^ assessed fewer treatments,^11^ and included a smaller number of trials.^9–11^

Although our study suggests a potential class-effect benefit of anti-inflammatory therapy, some differences depending on the drug type may be hypothesized. Specifically, no significant reduction in MACE was observed with drugs targeting initial macrophage recruitment by inhibiting the complement system (e.g. pexelizumab) or interacting with adhesion molecules (e.g. inclacumab), as well as with drugs affecting immune cell proliferation (e.g. everolimus, cyclosporine, and methotrexate).^17,18,23,25,26,32,39^ In contrast, drugs targeting the NLRP3/IL -1/IL -6 pathway demonstrated potential benefits.^8,30,36,38^ The CANTOS trial was the first large study to clearly show the efficacy of targeting this pathway, with canakinumab significantly reducing MACE, though at the cost of a higher risk of fatal infections or sepsis.^30^ Our study does not add new evidence on canakinumab, as CANTOS remains the only large-scale trial assessing its efficacy for secondary cardiovascular prevention.

In contrast, targeting the inflammatory pathway upstream by inhibiting NLRP3 activation with colchicine has produced mixed results over time.^46^ This is particularly evident in smaller trials such as LODOCO-MI, Podcast-MI, and COVERT-MI, which yielded neutral results.^35,40,41^ Conversely, larger studies like COLCOT and LoDoCO2 confirmed statistically significant and clinically relevant benefits of colchicine in reducing MACE.^7,8,36,38^ A key exception is the recent CLEAR SYNERGY (OASIS 9) trial, which, despite being the largest study on colchicine after MI, showed neutral results for ischaemic endpoints in the colchicine vs. placebo comparison.^6^ This discrepancy in treatment effects may be attributed to differences between the trials. Notably, CLEAR SYNERGY (OASIS 9) recruited more than 95% of patients with STEMI and administered colchicine immediately after the index event, whereas other trials mostly enrolled patients with prior MI and started colchicine treatment 30 days after the event. Furthermore, a significant interaction with colchicine dosage was observed in the CLEAR SYNERGY (OASIS 9) trial, where patients weighting more than 70 kg benefited more from a twice-daily regimen, suggesting that higher doses may be needed for this higher-risk population. However, the narrow therapeutic index of colchicine limits the potential for dose escalation.

Despite the differences between trials and the neutral results from the CLEAR SYNERGY (OASIS 9) study, our pooled analysis overall confirmed the beneficial effects of colchicine for secondary prevention after MI. In assessing the merits of a drug, it is essential to look at the totality of the evidence. Subgroup analysis of colchicine trials showed a significant reduction in MACE and recurrent MI in this population, consistent with a prior meta-analysis of five colchicine trials in patients with ischaemic heart disease.^9^ While this reduction did not translate into a significant decrease in revascularization, this effect seems to be influenced by the inclusion of trials with different designs and populations. Indeed, removing studies that introduced heterogeneity revealed a significant reduction in revascularization for colchicine, suggesting that further efforts should focus on identifying the correct patient subgroups and optimal timing of administration.

Given colchicine's relatively narrow therapeutic index and potential interactions with certain medications, low-dose regimens and avoidance in cases of severe renal dysfunction should be prioritized. These strategies have shown a favourable safety profile and no clinically significant interactions with statins, as demonstrated after following-up more than 10 000 patients for a median of 2 years in the COLCOT and LoDoCO2 trials.^46^

Despite earlier concerns about increased MACE with anakinra and tocilizumab, based on small phase 2 trials, our study did not confirm these findings when pooling data for these two drugs.^24,31^ The idea of potential differences between drug types is consistent with the results of the network meta-analysis, suggesting that colchicine had the highest probability of ranking first for reducing MACE compared to other anti-inflammatory drugs.

According to the pathophysiological mechanisms of inflammation in ischaemic heart disease, the observed differences in the effects of anti-inflammatory therapy could be attributed to the timing of administration.^5^ In our analysis, we found no significant interaction between MACE and timing of administration. However, an interaction was detected for revascularization, with larger benefits seen with later administration of anti-inflammatory therapy compared to earlier initiation. This was also reflected in a significant benefit for revascularization in patients with stabilized MI, as opposed to those presenting during the acute phase. While biologically plausible—since later administration could reduce plaque progression without interfering with initial scar formation—the interpretation of this result should be cautious. None of the trials included in our analysis directly compared different timings of administration, so further investigation is needed to confirm this finding.

Meta-regression analyses revealed that beta-blockers attenuated the treatment effect of anti-inflammatory therapy for MI prevention. Beta-blockers have variable effects in patients with MI, with the most beneficial outcomes typically seen in those with post-ischaemic left ventricular dysfunction.^47,48^ This population is well represented in trials of anti-inflammatory therapy, which may explain the reduced effectiveness of anti-inflammatory treatments in the presence of beta-blocker use.

Despite the role of the immune system in infection and cancer prevention, we did not observe any statistically significant increase in serious adverse events with anti-inflammatory drugs, including all-cause death and pneumonia. Previous evidence suggesting a higher incidence of all-cause mortality with anti-inflammatory therapy largely came from small, underpowered trials, and observational registries, which reported increased mortality long after drug discontinuation. The LoDoCo-2 trial, while reporting a non-significant increase in all-cause mortality due to non-cardiac deaths, further raised concerns about the overall benefits of anti-inflammatory drugs in patients with MI.^8^ Our pooled analysis largely alleviates concerns regarding the safety of colchicine in the context of polypharmacy, as no differences in all-cause mortality were observed across a total cohort of 36 661 patient-years. This supports the hypothesis that earlier reports may have been affected by a type I error.^9,30,36,46^ Similarly, the COLCOT trial reported higher rates of pneumonia in patients receiving anti-inflammatory therapy,^7^ introducing significant heterogeneity within the analysis. Although this could be due to chance, the definition of a significant adverse event in the COLCOT trial was left to the discretion of the physician, except for events resulting in prolonged hospitalization or death. This may have led to an overestimation of event incidence, as other trials employed more stringent criteria. In contrast, our analysis did not find a significant increase in the risk of severe lower respiratory tract infections, even when including data from the COLCOT trial, further supporting the safety of these agents. Our study also found a significant increase in adverse gastrointestinal events, primarily induced by colchicine and methotrexate, both of which are associated with altered mucosal turnover.^49,50^ These findings align with previous evidence, showing that gastrointestinal effects are more common during the early phase of colchicine therapy, while they tend to persist longer with methotrexate.^49,50^

The findings of the TITIAN study should be interpreted in the light of several limitations. Firstly, since comparisons between drugs relied on indirect evidence, the fulfilment of the transitivity assumption is limited, making our findings hypothesis-generating. Secondly, the lack of inpatient data limited our ability to explore contribution of specific confounders on the treatment estimate, although multiple sensitivity and meta-regression analyses have been conducted to mitigate this risk. Thirdly, some endpoints had heterogeneous definitions among the included trials; however, multiple sensitivity analyses and a hierarchical selection of definitions—when feasible—have been performed. Fourthly, including trials of drugs at different stages of development may have introduced some heterogeneity; however, restricting the analysis to phase-3 trials provided results consistent with the main analysis. Lastly, some endpoints were associated with moderate heterogeneity, which may limit the generalizability of the study results; however, replicating the analyses after the removal of trials mostly contributing to such heterogeneity led to results consistent with the main analysis.

## Conclusions

In patients with acute or prior MI, anti-inflammatory therapy was associated with a significant reduction in MACE. Among the anti-inflammatory drugs, colchicine had the highest probability of ranking first for reducing MACE. Despite an increased risk of gastrointestinal adverse events, anti-inflammatory therapy was not associated with an excess of serious adverse events, including severe infections, sepsis, or cancer development.

## Supplementary Material

pvae100_Supplemental_File

## Notes

**Conflict of interes**. All authors declare that they have no competing interest relevant to the topic of this manuscript.

## Data availability

All data are available upon reasonable request to the corresponding author.
