# Supplementary material for: A pairwise and network meta-analysis of anti-inflammatory strategies after myocardial infarction: the TITIAN study
Source: Eur Heart J Cardiovasc Pharmacother. 2025 Jan 3;11(3):218–29. doi: 10.1093/ehjcvp/pvae100 (PMC12046582; doi:10.1093/ehjcvp/pvae100)
Supplement: pvae100_Supplemental_File [file pvae100_supplemental_file.docx]

**SUPPLEMENTARY APPENDIX**

**Targeting InflammaTIon After myocardial iNfarction: the TITIAN study, a Systematic Review and Pairwise and Network Meta-analysis encompassing 37,344 patients from 27 randomized controlled trials**

Claudio Laudani, MD, MSc,^1^ Giovanni Occhipinti, MD,^1,2^ Antonio Greco, MD,^1^

Daniele Giacoppo, MD, MSc, PhD,^1^ Marco Spagnolo, MD^1^ and Davide Capodanno, MD, PhD^1^

^1^ Division of Cardiology, Azienda Ospedaliero-Universitaria Policlinico "Rodolico – San Marco", University of Catania, Catania, Italy.

^2^ Institut Clinic Cardiovascular, Hospital Clínic de Barcelona, University of Barcelona, Barcelona, Spain.

**INDEX OF CONTENTS**

**PROTOCOL REGISTRATION4
SUPPLEMENTARY METHODS16**

SUPPLEMENTARY TABLES18

Supplementary Table 1. Prisma checklist for pairwise meta-analysis. 18

Supplementary Table 2. Prisma checklist for network meta-analysis. 21

Supplementary Table 3. Full electronic search strategy through 31^st^ January 2024. 25

Supplementary Table 4. Selected characteristics of included trials. 26

Supplementary Table 5. Baseline characteristics across treatment in the included trials. 27

Supplementary Table 6. Available endpoints across included trials. 28

Supplementary Table 7. Available endpoint definitions across included trials. 29

Supplementary Table 8. Posterior assessment of the results according to GRADE approach. 36

Supplementary Table 9. Interaction analysis for timing of administration. 40

Supplementary Table 10. Indirect comparisons of anti-inflammatory drugs. 43

Supplementary Table 11. SUCRA values for prespecified endpoints. 49

Supplementary Table 12. Indirect comparisons on MACE, revascularization and pneumonia after removing influential trials.50

Supplementary Table 13. Sensitivity analyses for MACE.52

Supplementary Table 14. Sensitivity analyses for serious adverse events. 54

Supplementary Table 15. Sensitivity analyses for all-cause death. 56

Supplementary Table 16. Sensitivity analyses for cardiovascular death.58

Supplementary Table 17. Sensitivity analyses for myocardial infarction.60

Supplementary Table 18. Sensitivity analyses for heart failure.62

Supplementary Table 19. Sensitivity analyses for revascularization.65

Supplementary Table 20. Sensitivity analyses for stroke.66

Supplementary Table 21. Sensitivity analyses for serious infection or sepsis68

Supplementary Table 22. Sensitivity analyses for cancer development.70

Supplementary Table 23. Sensitivity analyses for pneumonia.72

Supplementary Table 24. Sensitivity analyses for gastrointestinal adverse events.74

Supplementary Table 25. Leave-one-out analysis. 76

Supplementary Table 26. Meta-regression analysis.83

SUPPLEMENTARY FIGURES85

Supplementary Figure 1. Systematic research flow.85

Supplementary Figure 2. Network of treatment. 86

Supplementary Figure 3. Overall risk of bias. 87

Supplementary Figure 4. Individual risk of bias across the included studies. 88

Supplementary Figure 5. Funnel plots and Egger’s test for publication bias. 89

Supplementary Figure 6. Influence analysis for MACE. 90

Supplementary Figure 7. Forest plot and interaction analysis for MACE after excluding MRC-ILA, STAT-MI and Akramy et al.91

Supplementary Figure 8. Forest plot and interaction analysis for all-cause death.92

Supplementary Figure 9. Forest plot and interaction analysis for cardiovascular death.93

Supplementary Figure 10. Forest plot and interaction analysis for myocardial infarction. 94

Supplementary Figure 11. Forest plot and interaction analysis for heart failure.95

Supplementary Figure 12. Forest plot and interaction analysis for revascularization.96

Supplementary Figure 13. Forest plot and interaction analysis for stroke.97

Supplementary Figure 14. Forest plot and interaction analysis for serious infection or sepsis.98

Supplementary Figure 15. Forest plot and interaction analysis for cancer development.99

Supplementary Figure 16. Forest plot and interaction analysis for pneumonia.100

Supplementary Figure 17. Forest plot and interaction analysis for gastrointestinal adverse events.101

Supplementary Figure 18. Influence analysis for revascularization.102

Supplementary Figure 19. Forest plot and interaction analysis for revascularization after excluding Australian COPS, MRC-ILA, SELECT-ACS and COLCOT trials.103

Supplementary Figure 20. Influence analysis for revascularization within the colchicine subgroup.104

Supplementary Figure 21. Forest plot for revascularization within the colchicine subgroup after excluding LoDoCo2 trial.105

Supplementary Figure 22. Influence analysis for pneumonia. 106

Supplementary Figure 23. Influence analysis for gastrointestinal adverse events. 107

Supplementary Figure 24. Forest plot and interaction analysis for pneumonia after removal of COLCOT trial.108

Supplementary Figure 25. Forest plot and interaction analysis for gastrointestinal adverse events after removal of Deftereos et al. trial.109

**PROTOCOL REGISTRATION**

**
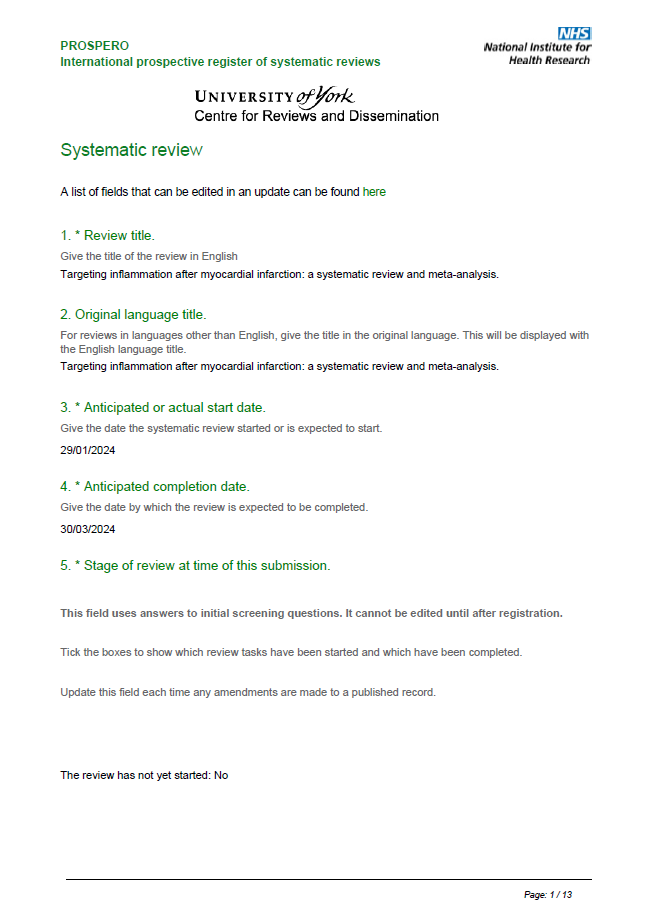
**

**
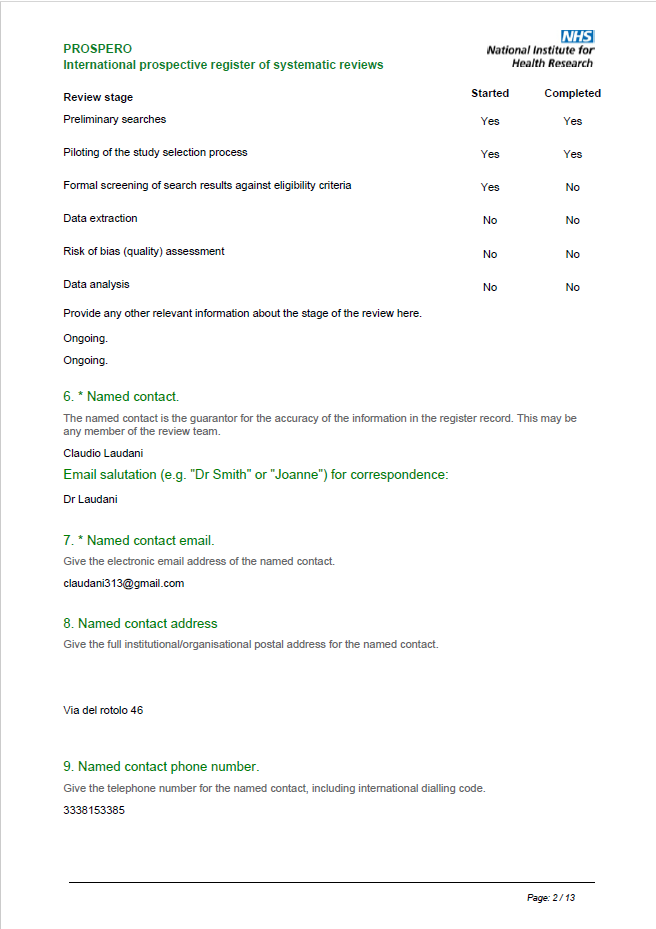
**

**
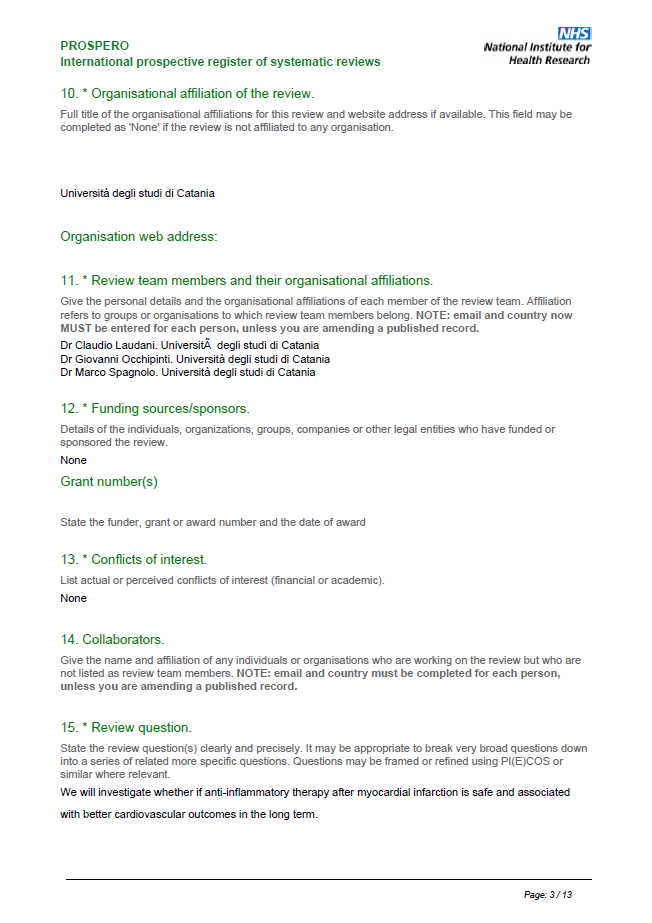
**

**
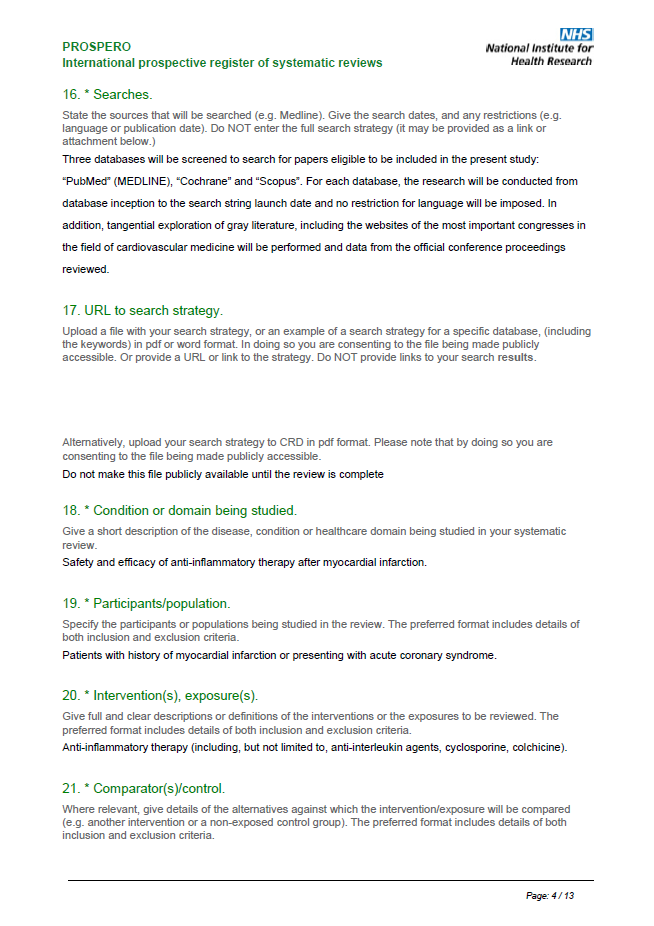
**

**
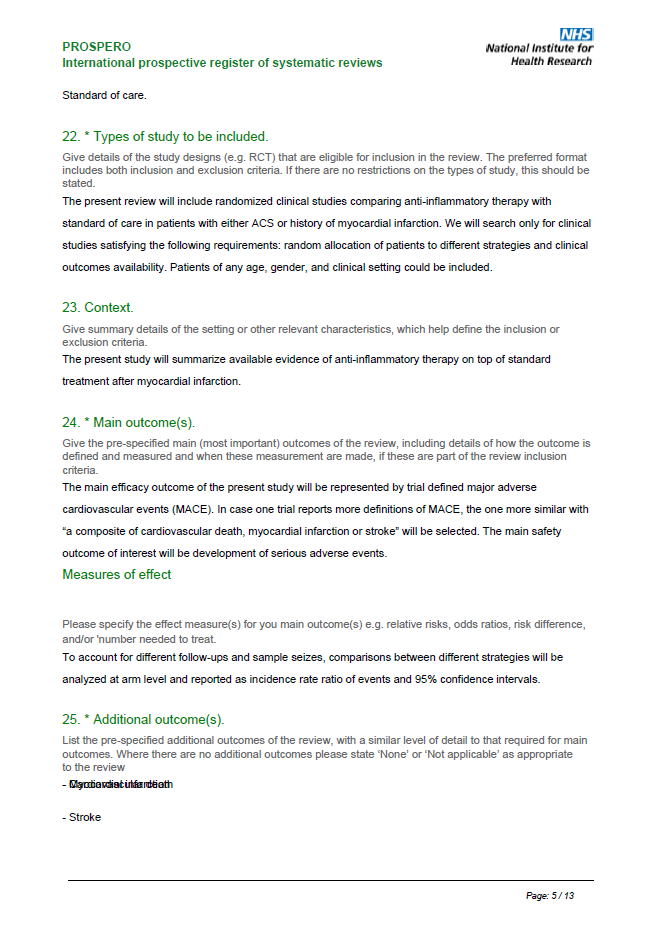
**

**
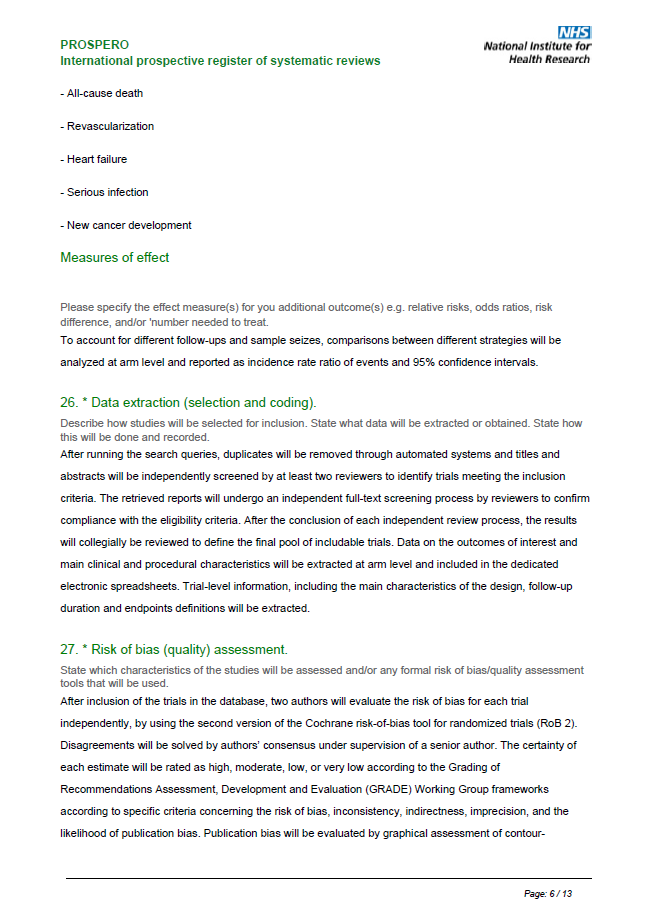
**

**
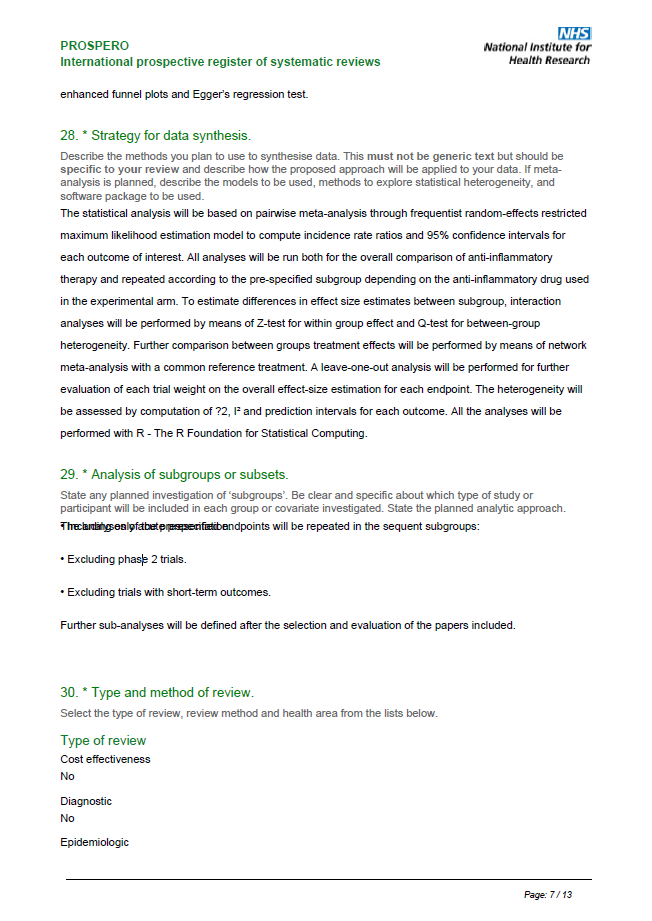
**

**
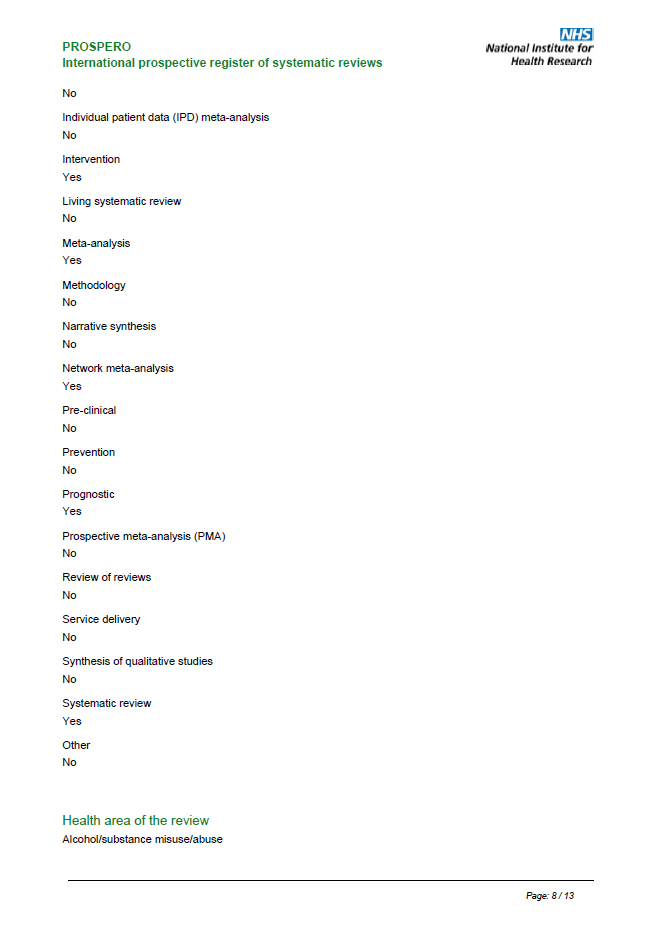
**

**
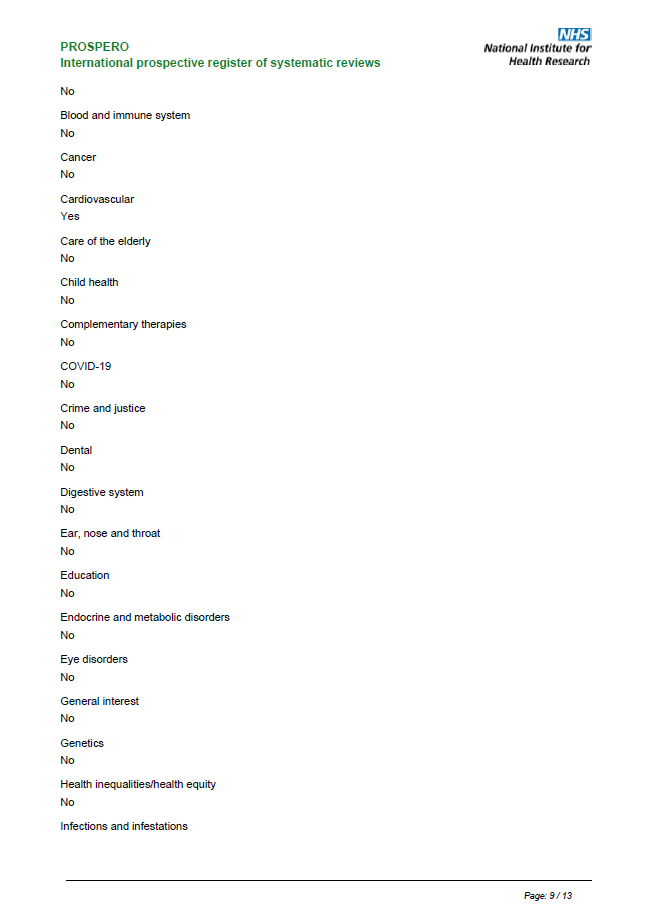
**

**
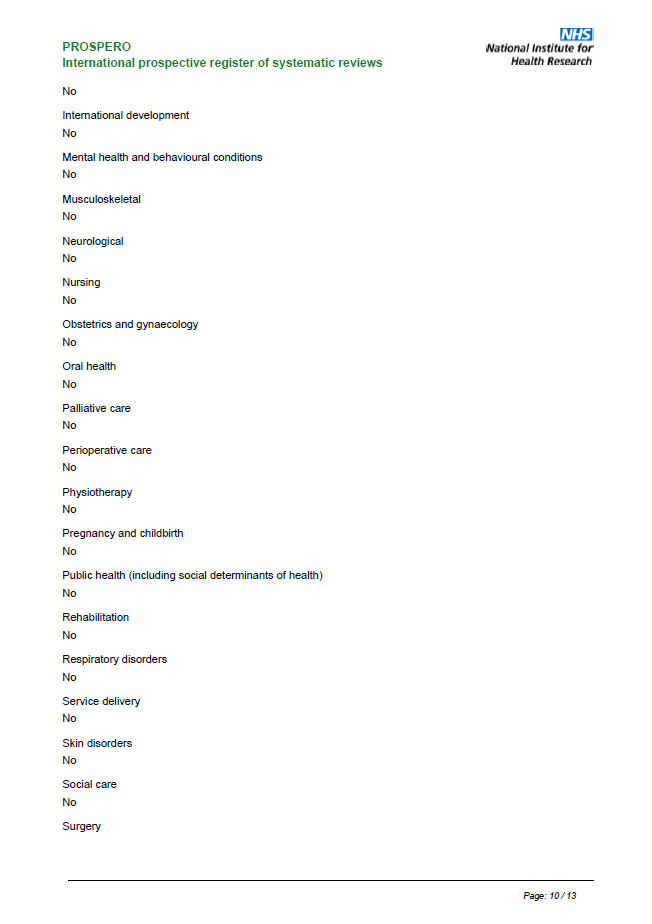
**

**
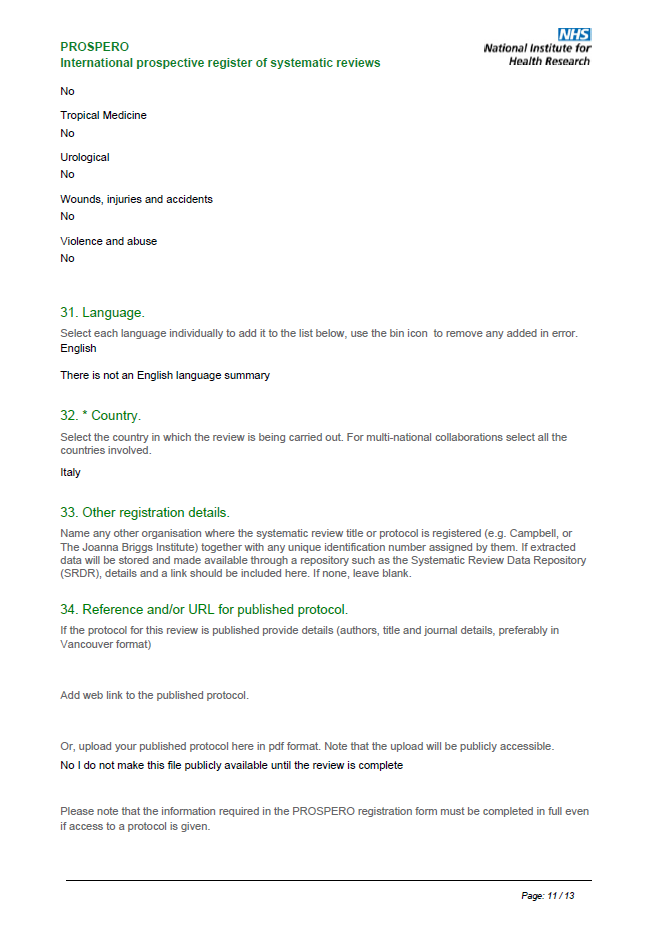
**

**
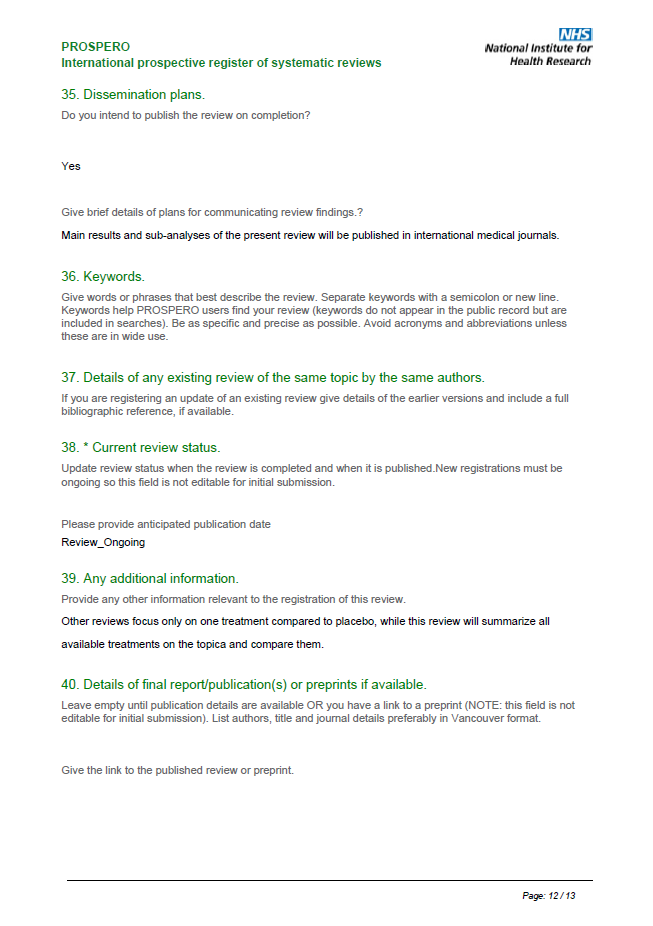
**

**SUPPLEMENTARY METHODS**

**Data extraction**

Information sources included MEDLINE through PubMED, Cochrane and Web of Science databases from inception to January 2024. The exploration of eligible studies extended to the examination of reference list through backward citation chasing. Two investigators (CL, GO) independently screened the studies at the title and abstract level, and the retrieved reports underwent independent full-text screening by the same reviewers to confirm possible eligibility in the meta-analysis according to the prespecified criteria. After completing each independent review process, the results were collectively reviewed, and discordant results were solved by consensus. Data pertaining to the outcomes of interest, clinical and procedural characteristics were extracted at the study level and incorporated into dedicated electronic spreadsheets, as well as key trial-level data encompassing design features, follow-up duration and endpoints definitions. Baseline characteristics are expressed as weighted means and percentages. Events in the investigational arms from trials exploring different dosages of the same drug were merged to avoid replication of control group and evaluate the treatment effect of the drug itself.

To reduce heterogeneity for the primary efficacy endpoint, we prioritized definitions more aligned with “the composite of cardiovascular death, myocardial infarction or stroke” in case of trials reporting multiple definitions of MACE.

**Qualitative assessment**

The risk-of-bias of each trial was assessed by two reviewers (CL, GO) through the Cochrane’s Risk of Bias (RoB) 2 tool, while posterior qualitative assessment of the meta-analysis results was performed in accordance with GRADE. Publication bias was assessed through visual inspection of contour-enhanced funnel plots and Egger’s regression test.

**Statistical analysis**

*Heterogeneity and influence analysis*

Statistical heterogeneity was assessed through *τ*^2^, I^2^ and prediction intervals, and ranked as low, moderate or high for I^2^ levels of <25%, 25% to 50% and >50%, respectively. Endpoints with moderate or high heterogeneity underwent exploration of individual trial contribution to heterogeneity against contribution to overall treatment effect, in order to detect possible outliers.

*Indirect comparisons*

After recalculation of each trial’s person-year and incidence rate ratio for estimate of treatment effects compared to placebo, indirect comparisons of all experimental drugs were performed by means of frequentist random-effect restricted maximum likelihood network meta-analysis using placebo as a common reference treatment. Treatments were ranked based on the surface under the cumulative ranking.

**Exploratory and sensitivity analyses**

Further exploratory analyses included interaction analysis to evaluate the influence of drug administration from presentation (i.e., periprocedural, immediately after revascularization and at late time) and influence of specific pathway targeted. Sensitivity analyses included restricting the analysis to trials with consistent definitions of MACE, patients with previous myocardial infarction, patients presenting in the acute phase, patients presenting with STEMI, phase 3 trials and trials with more than 6-month follow-up. Finally, meta-regression analyses were conducted to explore the influence of year of publication, age, female, diabetes, hypertension, statins and antiplatelet therapy on treatment effect estimates, and leave-one-out analysis was also conducted to evaluate specific influence of each trial on overall treatment effects and heterogeneity.

**SUPPLEMENTARY TABLES**

**Supplementary table 1. Prisma checklist for pairwise meta-analysis.**

| **Section and Topic** | **Item #** | **Checklist item** | **Location where item is reported** |
| --- | --- | --- | --- |
| **TITLE** | | |  |
| Title | 1 | Identify the report as a systematic review. | 1 |
| **ABSTRACT** | | |  |
| Abstract | 2 | See the PRISMA 2020 for Abstracts checklist. | 2 |
| **INTRODUCTION** | | |  |
| Rationale | 3 | Describe the rationale for the review in the context of existing knowledge. | 4-5 |
| Objectives | 4 | Provide an explicit statement of the objective(s) or question(s) the review addresses. | 5 |
| **METHODS** | | |  |
| Eligibility criteria | 5 | Specify the inclusion and exclusion criteria for the review and how studies were grouped for the syntheses. | 5 |
| Information sources | 6 | Specify all databases, registers, websites, organisations, reference lists and other sources searched or consulted to identify studies. Specify the date when each source was last searched or consulted. | 6 |
| Search strategy | 7 | Present the full search strategies for all databases, registers and websites, including any filters and limits used. | 6; S25 |
| Selection process | 8 | Specify the methods used to decide whether a study met the inclusion criteria of the review, including how many reviewers screened each record and each report retrieved, whether they worked independently, and if applicable, details of automation tools used in the process. | 6, S16 |
| Data collection process | 9 | Specify the methods used to collect data from reports, including how many reviewers collected data from each report, whether they worked independently, any processes for obtaining or confirming data from study investigators, and if applicable, details of automation tools used in the process. | 6, S16 |
| Data items | 10a | List and define all outcomes for which data were sought. Specify whether all results that were compatible with each outcome domain in each study were sought (e.g. for all measures, time points, analyses), and if not, the methods used to decide which results to collect. | 6; S16 |
|  | 10b | List and define all other variables for which data were sought (e.g. participant and intervention characteristics, funding sources). Describe any assumptions made about any missing or unclear information. | 6; S16 |
| Study risk of bias assessment | 11 | Specify the methods used to assess risk of bias in the included studies, including details of the tool(s) used, how many reviewers assessed each study and whether they worked independently, and if applicable, details of automation tools used in the process. | 6 |
| Effect measures | 12 | Specify for each outcome the effect measure(s) (e.g. risk ratio, mean difference) used in the synthesis or presentation of results. | 6 |
| Synthesis methods | 13a | Describe the processes used to decide which studies were eligible for each synthesis (e.g. tabulating the study intervention characteristics and comparing against the planned groups for each synthesis (item #5)). | 6 |
|  | 13b | Describe any methods required to prepare the data for presentation or synthesis, such as handling of missing summary statistics, or data conversions. | 6 |
|  | 13c | Describe any methods used to tabulate or visually display results of individual studies and syntheses. | 6-7 |
|  | 13d | Describe any methods used to synthesize results and provide a rationale for the choice(s). If meta-analysis was performed, describe the model(s), method(s) to identify the presence and extent of statistical heterogeneity, and software package(s) used. | 6-7 |
|  | 13e | Describe any methods used to explore possible causes of heterogeneity among study results (e.g. subgroup analysis, meta-regression). | 6-7 |
|  | 13f | Describe any sensitivity analyses conducted to assess robustness of the synthesized results. | 7; S17 |
| Reporting bias assessment | 14 | Describe any methods used to assess risk of bias due to missing results in a synthesis (arising from reporting biases). | 6-7 |
| Certainty assessment | 15 | Describe any methods used to assess certainty (or confidence) in the body of evidence for an outcome. | 7; S17 |
| **RESULTS** | | |  |
| Study selection | 16a | Describe the results of the search and selection process, from the number of records identified in the search to the number of studies included in the review, ideally using a flow diagram. | 7-8 |
|  | 16b | Cite studies that might appear to meet the inclusion criteria, but which were excluded, and explain why they were excluded. | S84 |
| Study characteristics | 17 | Cite each included study and present its characteristics. | 7-8, 21-22, S26-S27 |
| Risk of bias in studies | 18 | Present assessments of risk of bias for each included study. | 8, S86-S87 |
| Results of individual studies | 19 | For all outcomes, present, for each study: (a) summary statistics for each group (where appropriate) and (b) an effect estimate and its precision (e.g. confidence/credible interval), ideally using structured tables or plots. | 8-11; S91-S100 |
| Results of syntheses | 20a | For each synthesis, briefly summarise the characteristics and risk of bias among contributing studies. | 8-11 |
|  | 20b | Present results of all statistical syntheses conducted. If meta-analysis was done, present for each the summary estimate and its precision (e.g. confidence/credible interval) and measures of statistical heterogeneity. If comparing groups, describe the direction of the effect. | 8-11 |
|  | 20c | Present results of all investigations of possible causes of heterogeneity among study results. | 11 |
|  | 20d | Present results of all sensitivity analyses conducted to assess the robustness of the synthesized results. | 11 |
| Reporting biases | 21 | Present assessments of risk of bias due to missing results (arising from reporting biases) for each synthesis assessed. | 8 |
| Certainty of evidence | 22 | Present assessments of certainty (or confidence) in the body of evidence for each outcome assessed. | 8; S36 |
| **DISCUSSION** | | |  |
| Discussion | 23a | Provide a general interpretation of the results in the context of other evidence. | 11-14 |
|  | 23b | Discuss any limitations of the evidence included in the review. | 14-15 |
|  | 23c | Discuss any limitations of the review processes used. | 14-15 |
|  | 23d | Discuss implications of the results for practice, policy, and future research. | 14 |
| **OTHER INFORMATION** | | |  |
| Registration and protocol | 24a | Provide registration information for the review, including register name and registration number, or state that the review was not registered. | 5 |
|  | 24b | Indicate where the review protocol can be accessed, or state that a protocol was not prepared. | 5; S4 |
|  | 24c | Describe and explain any amendments to information provided at registration or in the protocol. | 5 |
| Support | 25 | Describe sources of financial or non-financial support for the review, and the role of the funders or sponsors in the review. | 1 |
| Competing interests | 26 | Declare any competing interests of review authors. | 1 |
| Availability of data, code and other materials | 27 | Report which of the following are publicly available and where they can be found: template data collection forms; data extracted from included studies; data used for all analyses; analytic code; any other materials used in the review. | 1 |

**Supplementary Table 2. PRISMA checklist for network meta-analyses.**

| **Section/Topic** | **Item #** | **Checklist Item** | **Reported on Page #** |
| --- | --- | --- | --- |
| **TITLE** |  |  |  |
| Title | 1 | Identify the report as a systematic review *incorporating a network meta-analysis (or related form of meta-analysis).* | ***1*** |
|  |  |  | ***2*** |
| **ABSTRACT** |  |  |  |
| Structured summary | 2 | Provide a structured summary including, as applicable:  **Background:** main objectives  **Methods:** data sources; study eligibility criteria, participants, and interventions; study appraisal; and *synthesis methods, such as network meta-analysis.*  **Results:** number of studies and participants identified; summary estimates with corresponding confidence/credible intervals; *treatment rankings may also be discussed. Authors may choose to summarize pairwise comparisons against a chosen treatment included in their analyses for brevity.*  **Discussion/Conclusions:** limitations; conclusions and implications of findings.  **Other:** primary source of funding; systematic review registration number with registry name. |  |
|  |  |  |  |
| **INTRODUCTION** |  |  |  |
| Rationale | 3 | Describe the rationale for the review in the context of what is already known*, including mention of why a network meta-analysis has been conducted.* | ***3-4*** |
| Objectives | 4 | Provide an explicit statement of questions being addressed, with reference to participants, interventions, comparisons, outcomes, and study design (PICOS). | 4 |
|  |  |  |  |
| **METHODS** |  |  |  |
| Protocol and registration | 5 | Indicate whether a review protocol exists and if and where it can be accessed (e.g., Web address); and, if available, provide registration information, including registration number. | 5 |
| Eligibility criteria | 6 | Specify study characteristics (e.g., PICOS, length of follow-up) and report characteristics (e.g., years considered, language, publication status) used as criteria for eligibility, giving rationale. *Clearly describe eligible treatments included in the treatment network, and note whether any have been clustered or merged into the same node (with justification).* | ***5*** |
| Information sources | 7 | Describe all information sources (e.g., databases with dates of coverage, contact with study authors to identify additional studies) in the search and date last searched. | 6; S25 |
| Search | 8 | Present full electronic search strategy for at least one database, including any limits used, such that it could be repeated. | S25 |
| Study selection | 9 | State the process for selecting studies (i.e., screening, eligibility, included in systematic review, and, if applicable, included in the meta-analysis). | 6-7; S25 |
| Data collection process | 10 | Describe method of data extraction from reports (e.g., piloted forms, independently, in duplicate) and any processes for obtaining and confirming data from investigators. | 7; S16 |
| Data items | 11 | List and define all variables for which data were sought (e.g., PICOS, funding sources) and any assumptions and simplifications made. | 5-6 |
| **Geometry of the network** | **S1** | Describe methods used to explore the geometry of the treatment network under study and potential biases related to it. This should include how the evidence base has been graphically summarized for presentation, and what characteristics were compiled and used to describe the evidence base to readers. | ***6; S17*** |
| Risk of bias within individual studies | 12 | Describe methods used for assessing risk of bias of individual studies (including specification of whether this was done at the study or outcome level), and how this information is to be used in any data synthesis. | 6; S86-S87 |
| Summary measures | 13 | State the principal summary measures (e.g., risk ratio, difference in means). *Also describe the use of additional summary measures assessed, such as treatment rankings and surface under the cumulative ranking curve (SUCRA) values, as well as modified approaches used to present summary findings from meta-analyses.* | 6 |
| Planned methods of analysis | 14 | Describe the methods of handling data and combining results of studies for each network meta-analysis. This should include, but not be limited to:   - *Handling of multi-arm trials;* - *Selection of variance structure;* - *Selection of prior distributions in Bayesian analyses; and* - *Assessment of model fit.* | 6-7 |
| **Assessment of Inconsistency** | **S2** | Describe the statistical methods used to evaluate the agreement of direct and indirect evidence in the treatment network(s) studied. Describe efforts taken to address its presence when found. | 7 |
| Risk of bias across studies | 15 | Specify any assessment of risk of bias that may affect the cumulative evidence (e.g., publication bias, selective reporting within studies). | **6** |
| Additional analyses | 16 | Describe methods of additional analyses if done, indicating which were pre-specified. This may include, but not be limited to, the following:   - Sensitivity or subgroup analyses; - Meta-regression analyses; - *Alternative formulations of the treatment network; and* - *Use of alternative prior distributions for Bayesian analyses (if applicable).* | ***7; S17*** |
|  |  |  |  |
| **RESULTS†** |  |  |  |
| Study selection | 17 | Give numbers of studies screened, assessed for eligibility, and included in the review, with reasons for exclusions at each stage, ideally with a flow diagram. | 7; S84 |
| **Presentation of network structure** | **S3** | Provide a network graph of the included studies to enable visualization of the geometry of the treatment network. | ***S85*** |
| **Summary of network geometry** | **S4** | Provide a brief overview of characteristics of the treatment network. This may include commentary on the abundance of trials and randomized patients for the different interventions and pairwise comparisons in the network, gaps of evidence in the treatment network, and potential biases reflected by the network structure. | ***7*** |
| Study characteristics | 18 | For each study, present characteristics for which data were extracted (e.g., study size, PICOS, follow-up period) and provide the citations. | 8, 21-22; S26-S27 |
| Risk of bias within studies | 19 | Present data on risk of bias of each study and, if available, any outcome level assessment. | 7-8; S36, S86-S87 |
| Results of individual studies | 20 | For all outcomes considered (benefits or harms), present, for each study: 1) simple summary data for each intervention group, and 2) effect estimates and confidence intervals. *Modified approaches may be needed to deal with information from larger networks.* | ***8-11; S91-S100*** |
| Synthesis of results | 21 | Present results of each meta-analysis done, including confidence/credible intervals. *In larger networks, authors may focus on comparisons versus a particular comparator (e.g. placebo or standard care), with full findings presented in an appendix. League tables and forest plots may be considered to summarize pairwise comparisons.* If additional summary measures were explored (such as treatment rankings), these should also be presented. | ***8-11; S42*** |
| **Exploration for inconsistency** | **S5** | Describe results from investigations of inconsistency. This may include such information as measures of model fit to compare consistency and inconsistency models, *P* values from statistical tests, or summary of inconsistency estimates from different parts of the treatment network. | ***8-11; S49*** |
| Risk of bias across studies | 22 | Present results of any assessment of risk of bias across studies for the evidence base being studied. | 8; S86-S87 |
| Results of additional analyses | 23 | Give results of additional analyses, if done (e.g., sensitivity or subgroup analyses, meta-regression analyses*, alternative network geometries studied, alternative choice of prior distributions for Bayesian analyses,* and so forth). | ***S49-S82*** |
|  |  |  |  |
| **DISCUSSION** |  |  |  |
| Summary of evidence | 24 | Summarize the main findings, including the strength of evidence for each main outcome; consider their relevance to key groups (e.g., healthcare providers, users, and policy-makers). | 11-14 |
| Limitations | 25 | Discuss limitations at study and outcome level (e.g., risk of bias), and at review level (e.g., incomplete retrieval of identified research, reporting bias). *Comment on the validity of the assumptions, such as transitivity and consistency. Comment on any concerns regarding network geometry (e.g., avoidance of certain comparisons).* | 14-15 |
| Conclusions | 26 | Provide a general interpretation of the results in the context of other evidence, and implications for future research. | 15 |
|  |  |  | 1 |
| **FUNDING** |  |  |  |
| Funding | 27 | Describe sources of funding for the systematic review and other support (e.g., supply of data); role of funders for the systematic review. This should also include information regarding whether funding has been received from manufacturers of treatments in the network and/or whether some of the authors are content experts with professional conflicts of interest that could affect use of treatments in the network. |  |

PICOS = population, intervention, comparators, outcomes, study design.

**Supplementary Table 3.** **Full electronic search strategy through 31^st^ January 2024.**

| **Database** | **Search string** | **N° of results** |
| --- | --- | --- |
| MedLine | ((Myocardial infarction [MeSH Terms] OR acute coronary syndrome[MeSH Terms] OR "Myocardial infarct*"[tiab] OR "acute coronary syndrome*"[tiab]) AND ("antiinflammatory"[tiab]OR "canakinumab"[tiab] OR canakinumab[MeSH Terms] OR "Tocilizumab"[tiab] OR Tocilizumab[MeSH Terms] OR "Pexelizumab"[tiab] OR Pexelizumab[MeSH Terms] OR Cyclosporine[MeSH Terms] OR "Cyclosporine"[tiab] OR Methotrexate[MeSH Terms] OR "Methotrexate"[tiab] OR Everolimus[MeSH Terms] OR "Everolimus"[tiab] OR Colchicine[MeSH Terms] OR "Colchicine"[tiab] OR Anakinra[MeSH Terms] OR "Anakinra"[tiab] OR Interleukin-6 Inhibitors[MeSh Terms] OR Interleukin 1 Receptor Antagonist Protein [MeSH Terms])AND (Randomized controlled trial [MeSH Terms]OR Random*[tiab]) | 1,398 |
| Cochrane Library | ([mh "Myocardial infarction"] OR [mh "acute coronary syndrome"] OR ("Myocardial" NEXT infarct*):ti,ab OR ("acute coronary" NEXT syndrome*):ti,ab) AND ("antiinflammatoryOR “canakinumab”":ti,ab OR [mh canakinumab] OR Tocilizumab:ti,ab OR [mh Tocilizumab] OR Pexelizumab:ti,ab OR [mh Pexelizumab] OR [mh Cyclosporine] OR Cyclosporine:ti,ab OR [mh Methotrexate] OR Methotrexate:ti,ab OR [mh Everolimus] OR Everolimus:ti,ab OR [mh Colchicine] OR Colchicine:ti,ab OR [mh Anakinra] OR Anakinra:ti,ab OR [mh "Interleukin-6 Inhibitors"] OR [mh "Interleukin 1 Receptor Antagonist Protein"]) | 131 |
| Web of Science | (INDEXTERMS("Myocardial infarction") OR INDEXTERMS("acute coronary syndrome") OR TITLE-ABS("Myocardial infarct*") OR TITLE-ABS("acute coronary syndrome*")) AND (TITLE-ABS("antiinflammatoryOR “canakinumab”") OR INDEXTERMS(canakinumab) OR TITLE-ABS(Tocilizumab) OR INDEXTERMS(Tocilizumab) OR TITLE-ABS(Pexelizumab) OR INDEXTERMS(Pexelizumab) OR INDEXTERMS(Cyclosporine) OR TITLE-ABS(Cyclosporine) OR INDEXTERMS(Methotrexate) OR TITLE-ABS(Methotrexate) OR INDEXTERMS(Everolimus) OR TITLE-ABS(Everolimus) OR INDEXTERMS(Colchicine) OR TITLE-ABS(Colchicine) OR INDEXTERMS(Anakinra) OR TITLE-ABS(Anakinra) OR INDEXTERMS("Interleukin-6 Inhibitors") OR INDEXTERMS("Interleukin 1 Receptor Antagonist Protein")) AND (INDEXTERMS("Randomized controlled trial") OR TITLE-ABS("Randomized controlled trial")) | 1,333 |

**Supplementary Table 4. Selected characteristics of included trials.**

| **Study, year** | **Number of countries** | **Beta-blockers (%)** | **ACE-I or ARB (%)** | **Timing of first experimental drug administration** |
| --- | --- | --- | --- | --- |
| **COMPLY, 2003** | 8 | 45 | 43 | 10 minutes after fibrinolysis (median) |
| **COMMA, 2003** | NR | 36 | 31 | 16 minutes before PCI (median) |
| **APEX AMI, 2007** | 17 | 88 | 78 | 15 minutes before PCI (median) |
| **Piot et al., 2008** | NR | NR | NR | Between angiography and PCI |
| **Ghaffari et al., 2012** | 1 | 90 | 93 | Immediately before fibrinolysis |
| **VCU-ART2, 2013** | 1 | NR | NR | After reperfusion |
| **SELECT-ACS, 2013** | 66 | 91 | 90 | Between 1 and 24 hours before PCI |
| **MRC-ILA, 2014** | 1 | NR | NR | Within 48 hours from symptom onset |
| **CIRCUS, 2015** | 3 | NR | NR | 1 hour from hospital arrival (mean) |
| **CYCLE, 2015** | 1 | 81 | 72 | Between angiography and PCI |
| **Deftereos et al., 2015** | 1 | NR | NR | Between angiography and PCI |
| **COLIN, 2016** | 1 | NR | NR | First day of myocardial infarction |
| **Kleveland et al., 2016** | 1 | 77 | NR | 2 days from symptom onset (median) |
| **CANTOS, 2017** | 39 | NR | 80 | More than 30 days from the event |
| **STAT-MI, 2017** | 1 | 39 | 35 | Within 24 hours from admission |
| **TETHYS, 2017** | 1 | 83 | 83 | Between angiography and PCI |
| **CIRT, 2018** | 2 | 79 | 72 | More than 60 days from the event |
| **COLCOT, 2019** | 12 | 89 | NR | 13.5 days from the event (mean) |
| **VCUART3, 2019** | 1 | 84 | 85 | 8 hours from symptom onset (median) |
| **LoDoCo-MI, 2019** | 1 | 93 | 94 | Within 7 days from myocardial infarction |
| **LoDoCo2, 2020** | 2 | 62 | 72 | More than 30 days after the event |
| **Australian COPS, 2020** | 1 | 45 | 87 | During hospitalization |
| **ASSAIL-MI, 2021** | 1 | 1 | 23 | 150 minutes from symptom onset (mean) |
| **Akrami et al., 2021** | 1 | NR | NR | First day of myocardial infarction |
| **CLEVER-ACS, 2022** | 2 | 75 | 78 | Within 5 days from myocardial infarction |
| **PodCAST-PCI, 2022** | 1 | NR | NR | Between angiography and PCI |
| **COVERT-MI, 2023** | 1 | NR | NR | Within hospitalization |
| **CLEAR SINERGY (OASIS 9), 2024** | 14 | NR | 78 | Within hospitalization |

Abbreviations: ACE-I, angiotensin converting enzyme inhibitors; ARBs, angiotensin receptor blockers.

**Supplementary Table 5. Baseline characteristics across treatment in the included trials.**

| **Treatment** | **Age** | **Female (%)** | **Diabetes (%)** | **Hypertension (%)** | **Statins (%)** | **Antiplatelet therapy (%)** | **Beta-blockers (%)** | **ACE-I or ARBs (%)** |
| --- | --- | --- | --- | --- | --- | --- | --- | --- |
| Anakinra | 60.1 | 22.0 | 21.0 | 44.4 | 96.6 | 99.6 | 84.0 | 82.8 |
| Canakinumab | 61.1 | 25.7 | 40.0 | 79.6 | 91.0 | NR | NR | 80.0 |
| Colchicine | 62.7 | 22.5 | 19.6 | 49.9 | 96.4 | 99.3 | 72.8 | 74.6 |
| Cyclosporine | 60.9 | 18.7 | 14.8 | 43.62 | 91.0 | 95.1 | 82.8 | 76.2 |
| Everolimus | 61.9 | 14.7 | 9.3 | 40.7 | 86.7 | 92.7 | 75.0 | 78.0 |
| Inclacumab | 61.1 | 21.1 | 22.7 | NR | 95.1 | 93.2 | 91.0 | 90.0 |
| Methotrexate | 65.7 | 18.9 | 33.5 | 89.8 | 86.0 | 100.0 | 79.1 | 72.2 |
| Pexelizumab | 60.8 | 19.9 | 13.6 | NR | 75.4 | 79.7 | 77.1 | 68.6 |
| Tocilizumab | 61.4 | 14.3 | 15.2 | 20.5 | 47.0 | 99.7 | 29.9 | 24.4 |

Data are presented as weighted means or percentages.
Abbreviations: ACE-I, angiotensin converting enzyme inhibitors; ARBs, angiotensin receptor blockers.

**Supplementary Table 6. Available endpoints across included trials.**

| **Study, year** | **MACE** | **Serious adverse events** | **All-cause death** | **Cardiovascular death** | **Myocardial infarction** | **Heart failure** | **Revascularization** | **Stroke** | **Serious infection or sepsis** | **Cancer development** | **Pneumonia** | **Gastrointestina adverse events** |
| --- | --- | --- | --- | --- | --- | --- | --- | --- | --- | --- | --- | --- |
| **COMPLY, 2003** | 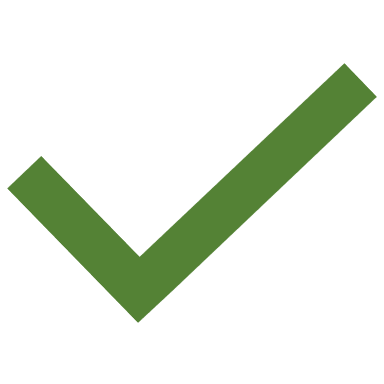 |  | 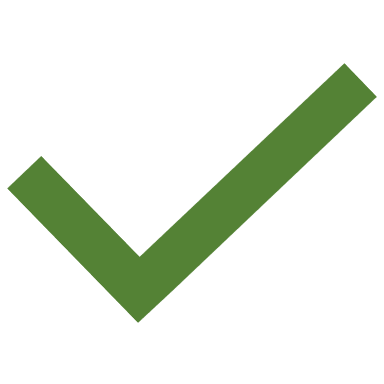 |  | 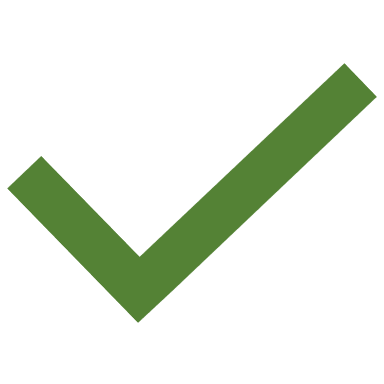 | 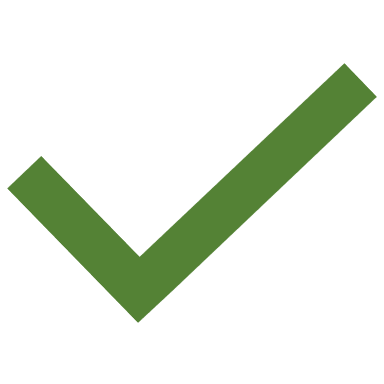 | 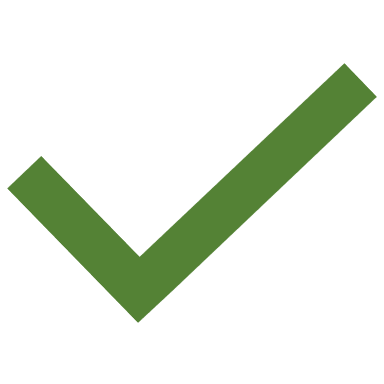 | 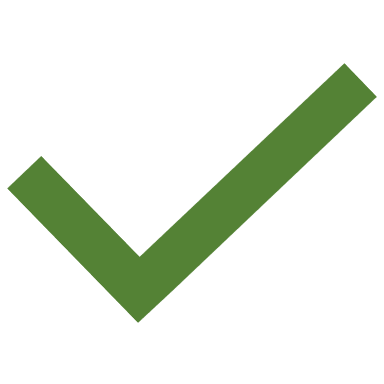 | 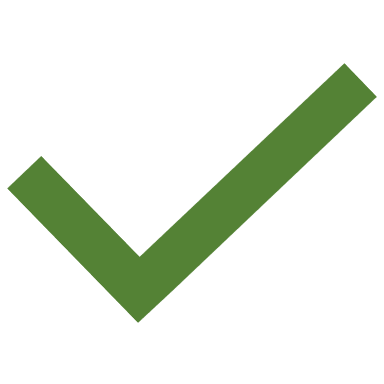 |  |  |  |
| **COMMA, 2003** | 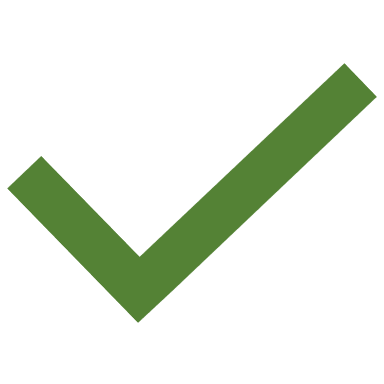 |  | 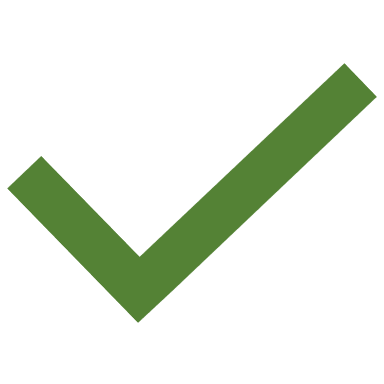 |  | 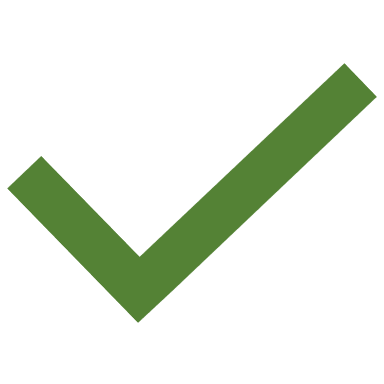 | 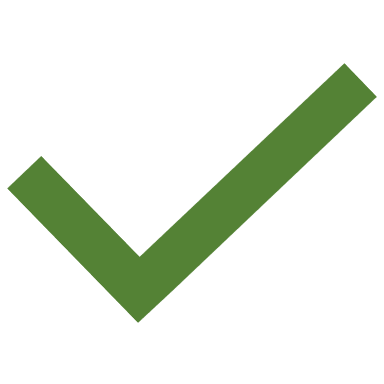 | 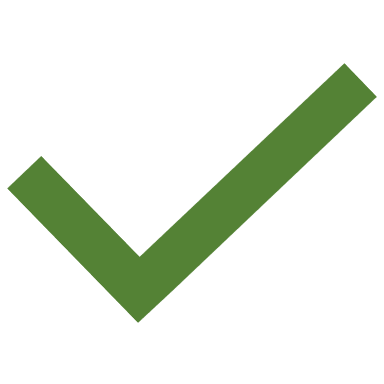 | 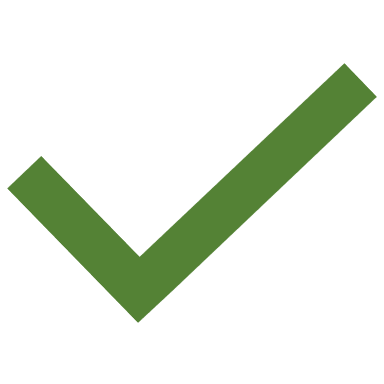 | 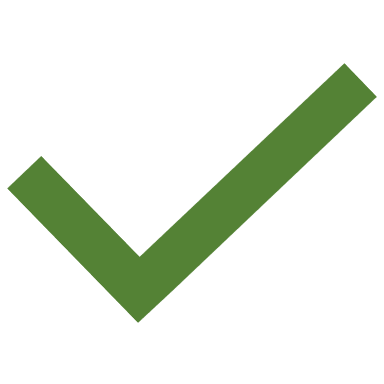 |  |  |  |
| **APEX AMI, 2007** | 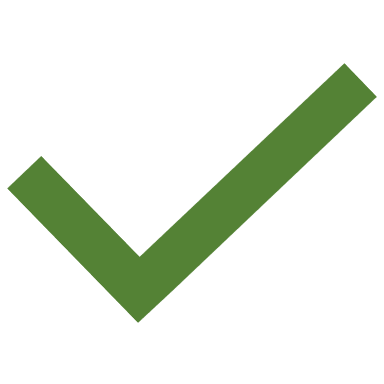 | 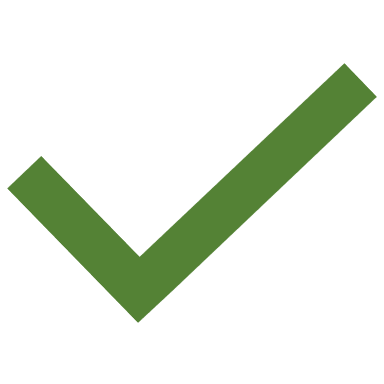 | 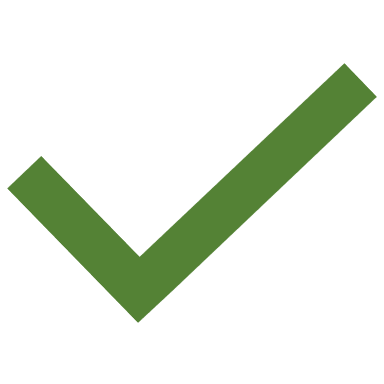 |  | 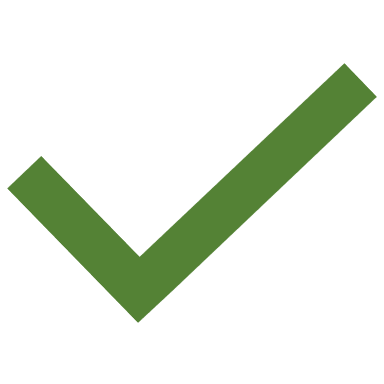 | 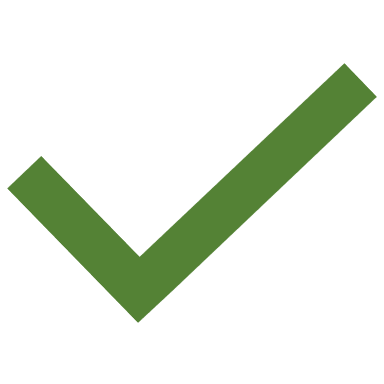 |  | 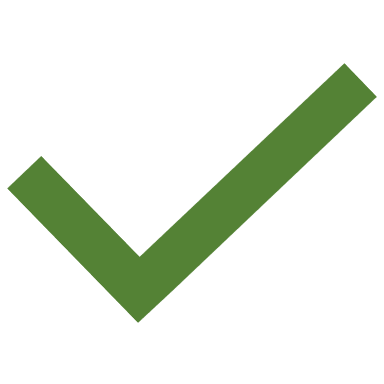 | 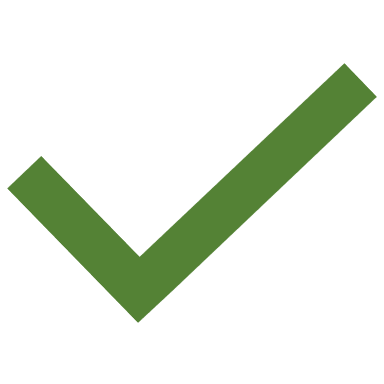 |  |  |  |
| **Piot et al., 2008** |  | 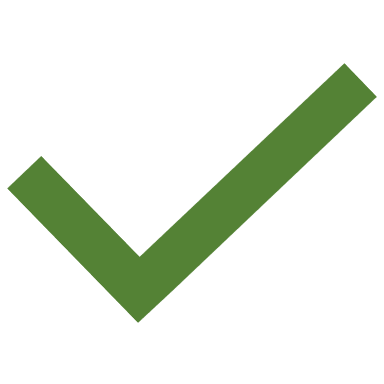 | 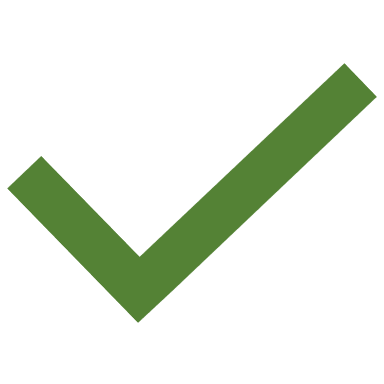 | 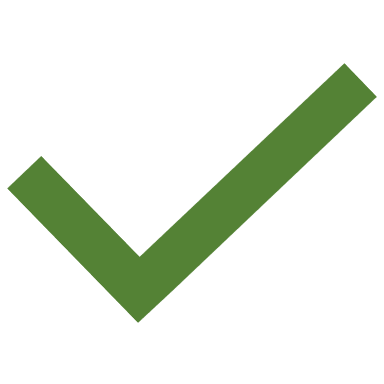 |  | 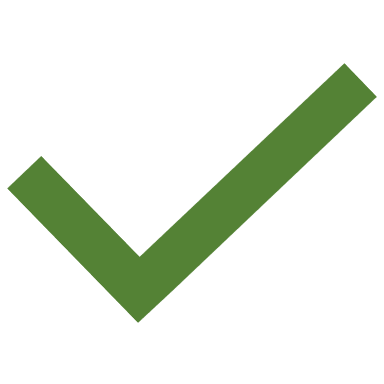 |  |  |  |  |  |  |
| **Ghaffari et al., 2012** |  |  | 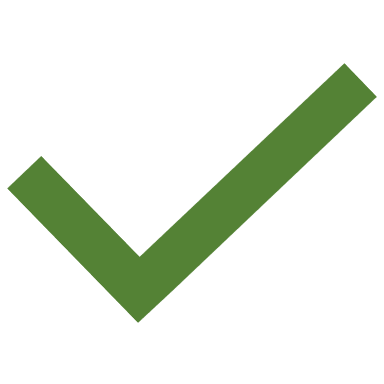 |  |  | 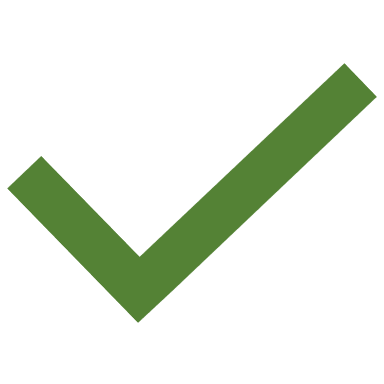 |  |  |  |  |  |  |
| **VCU-ART2, 2013** |  | 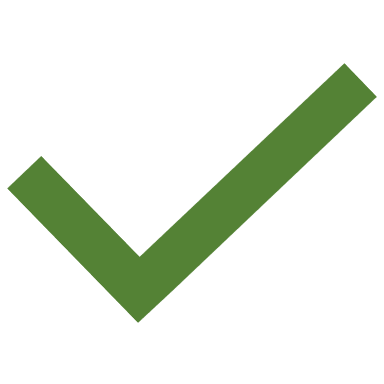 | 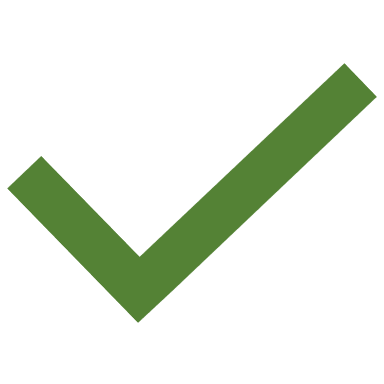 | 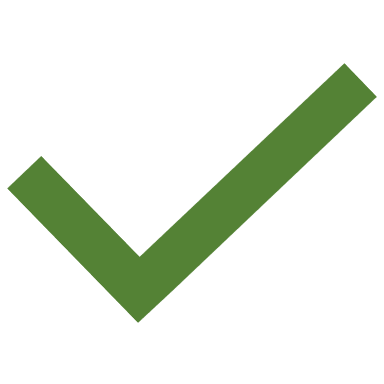 | 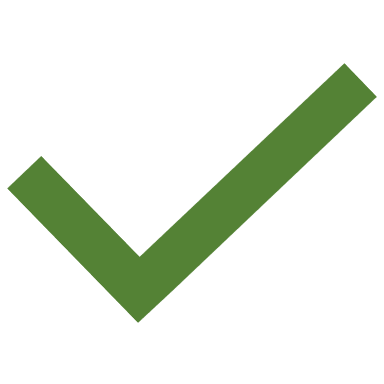 | 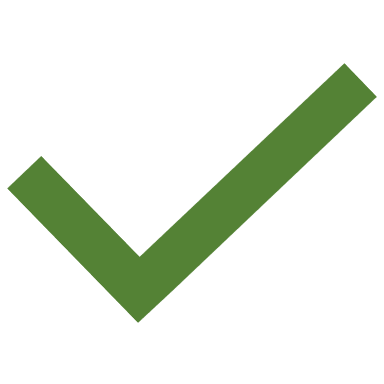 | 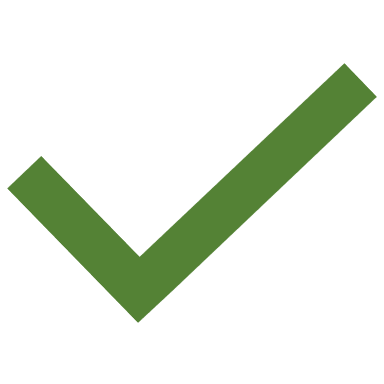 |  | 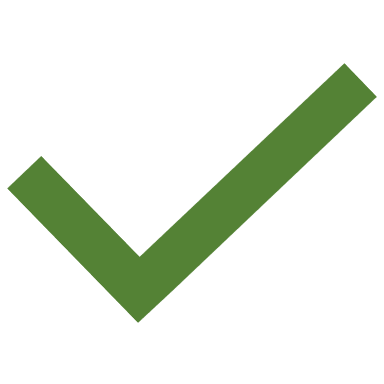 |  | 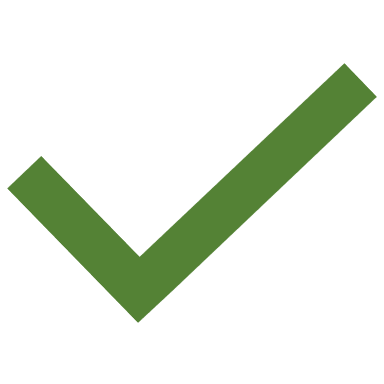 |  |
| **SELECT-ACS, 2013** |  | 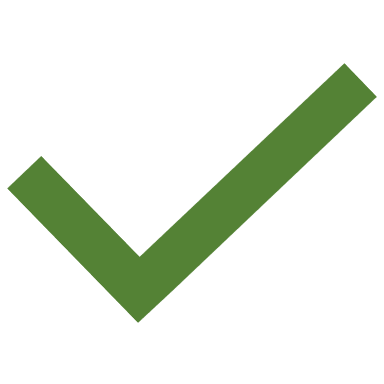 | 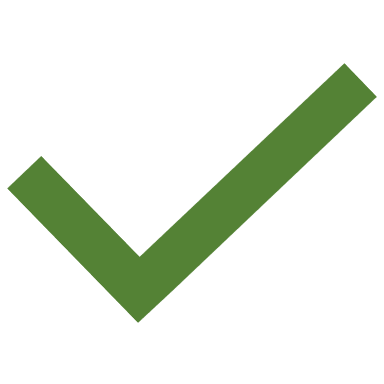 |  | 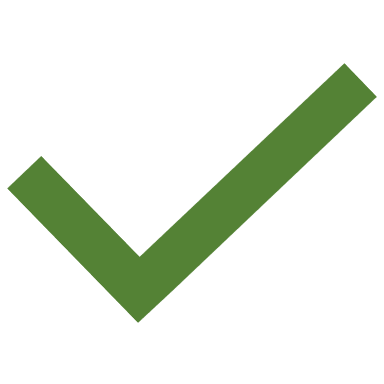 | 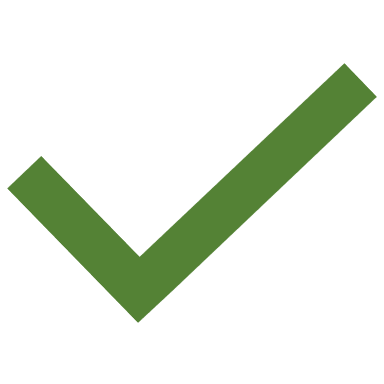 | 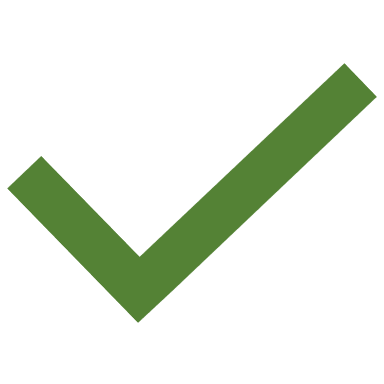 | 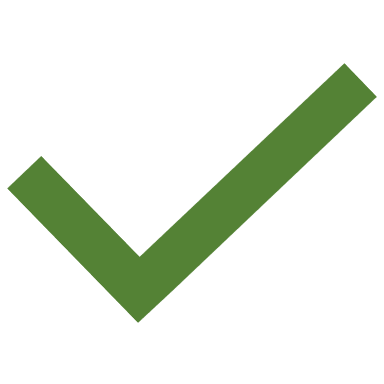 | 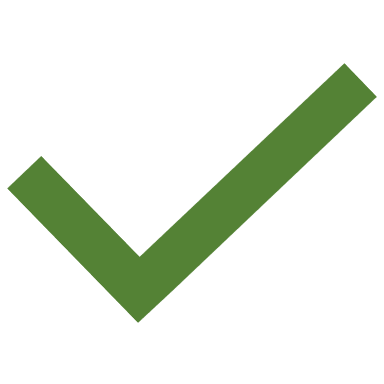 |  |  |  |
| **MRC-ILA, 2014** | 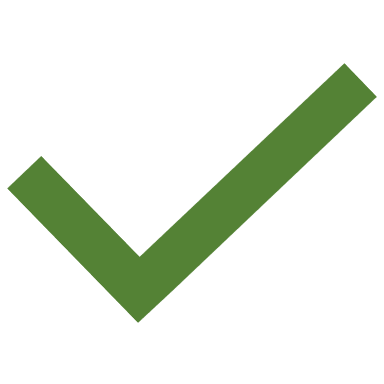 |  | 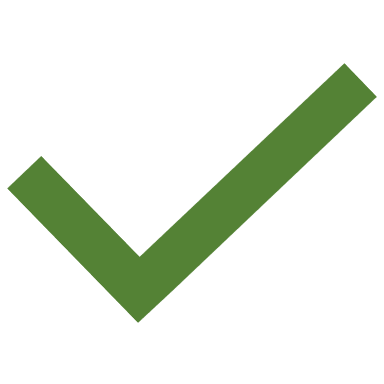 |  | 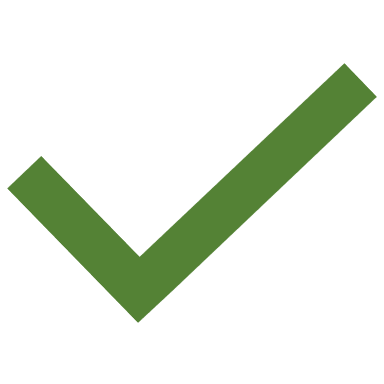 |  | 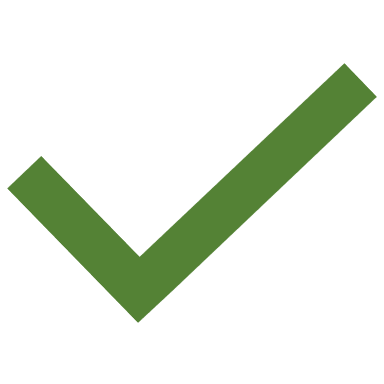 | 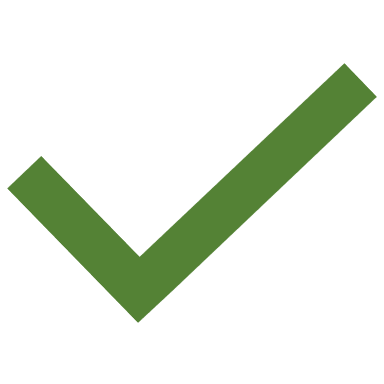 | 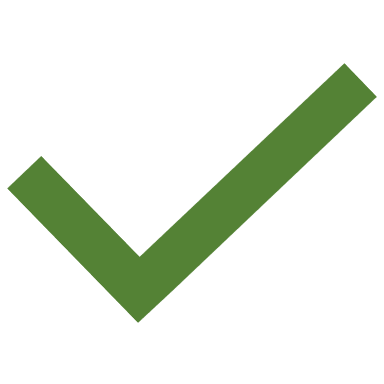 |  |  |  |
| **CIRCUS, 2015** | 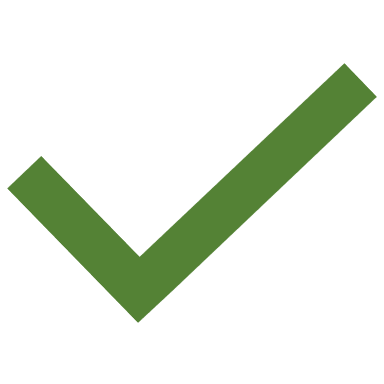 |  | 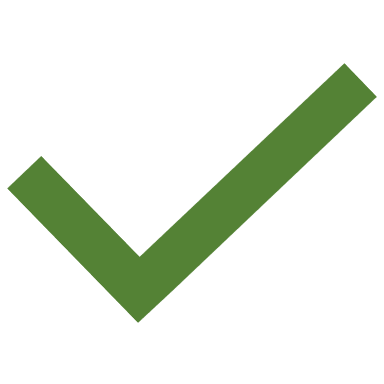 | 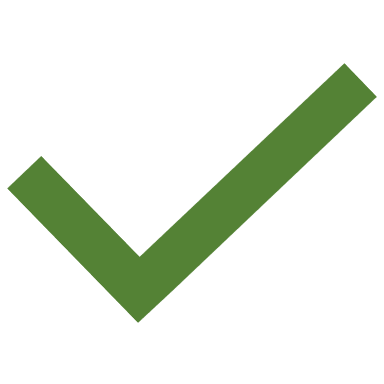 | 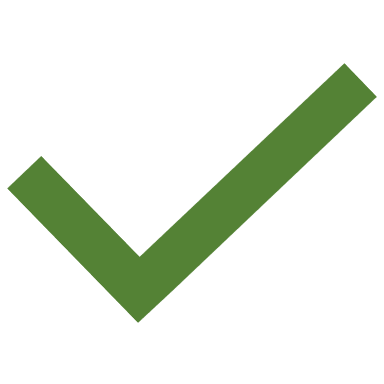 | 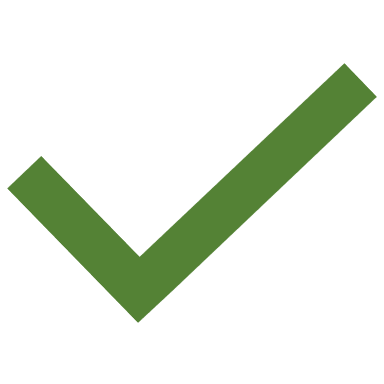 |  | 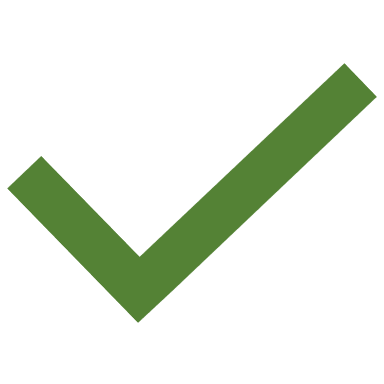 |  |  |  |  |
| **CYCLE, 2015** | 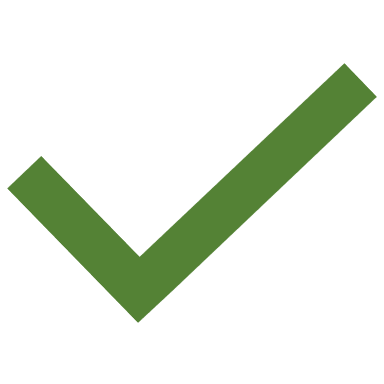 | 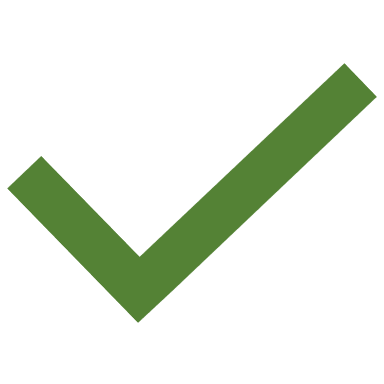 | 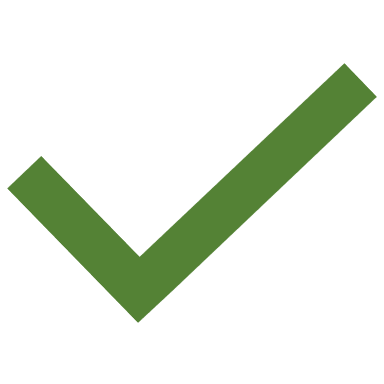 | 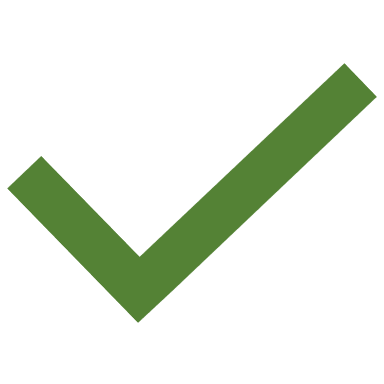 |  | 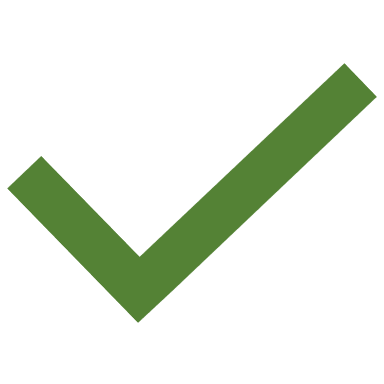 |  |  |  |  |  |  |
| **Deftereos et al., 2015** |  |  | 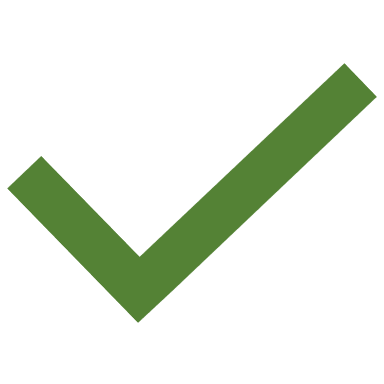 |  |  |  |  |  |  |  |  | 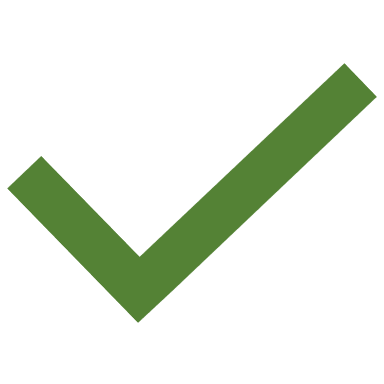 |
| **COLIN, 2016** |  |  |  |  | 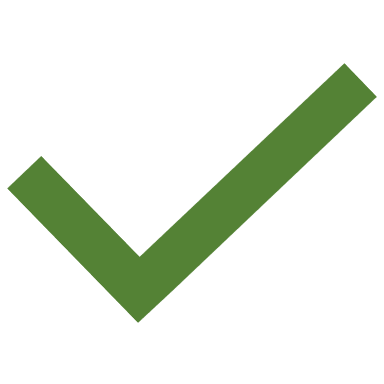 | 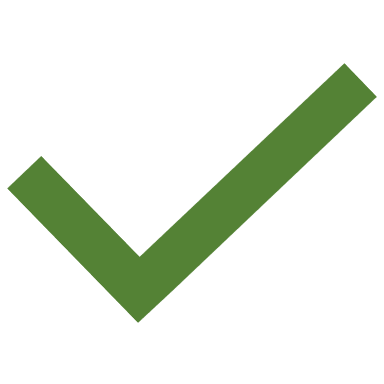 |  |  |  |  |  | 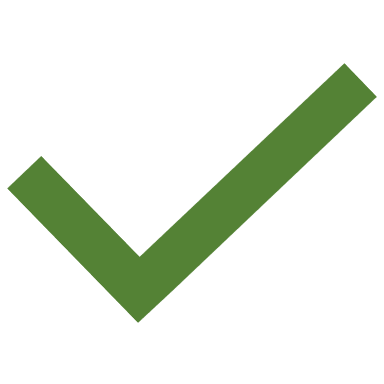 |
| **Kleveland et al., 2016** | 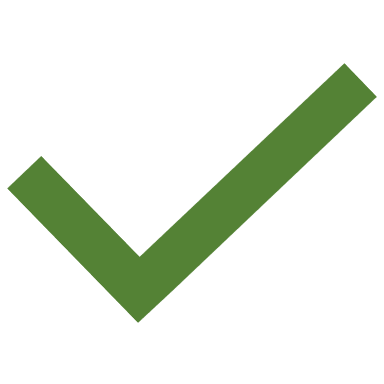 | 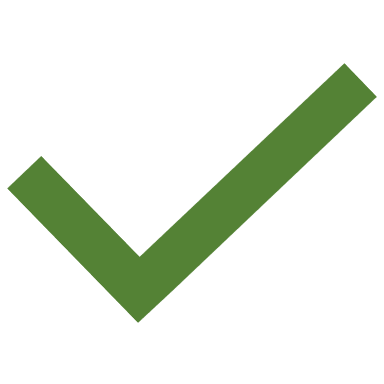 |  | 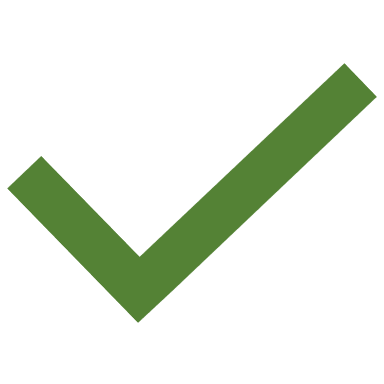 | 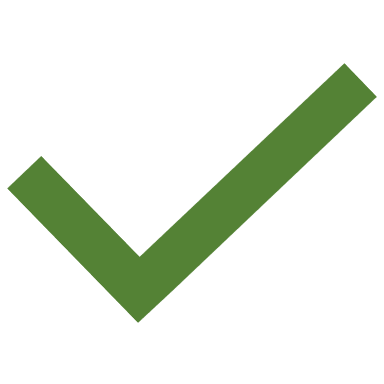 |  |  |  | 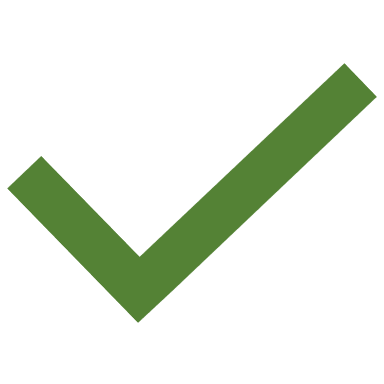 |  |  |  |
| **CANTOS, 2017** | 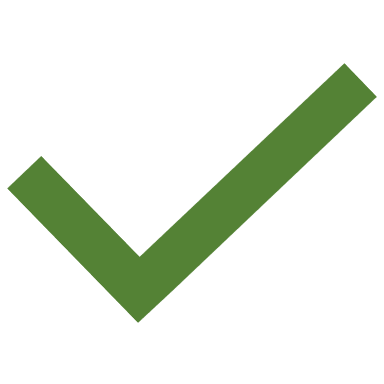 | 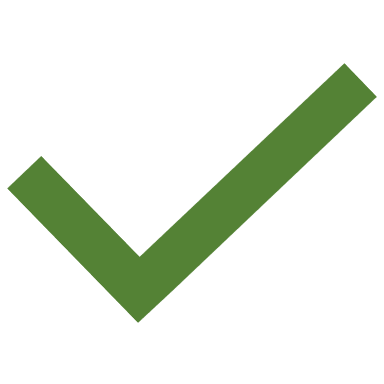 | 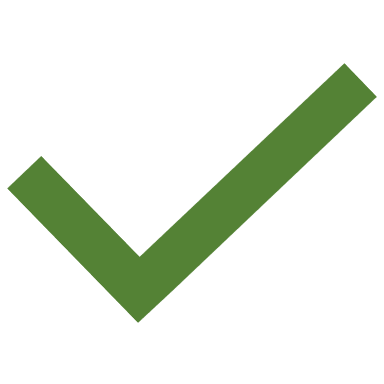 | 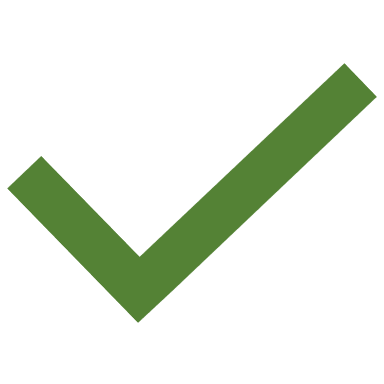 | 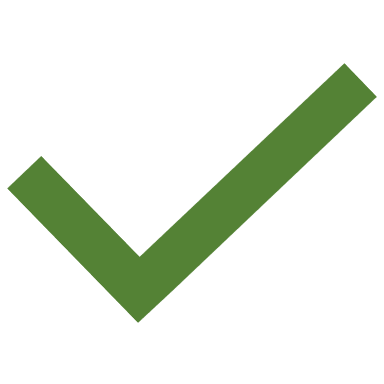 |  | 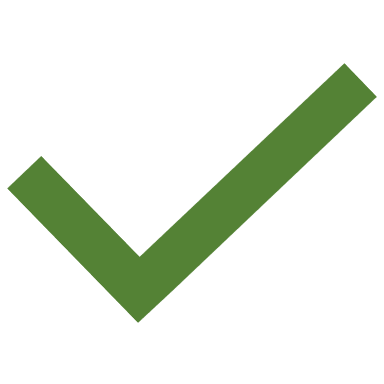 | 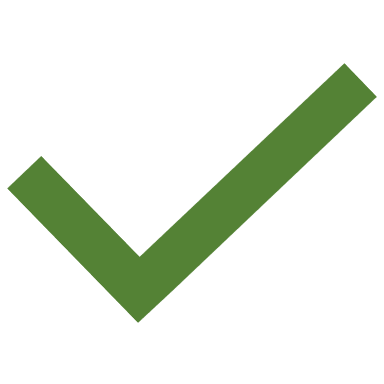 | 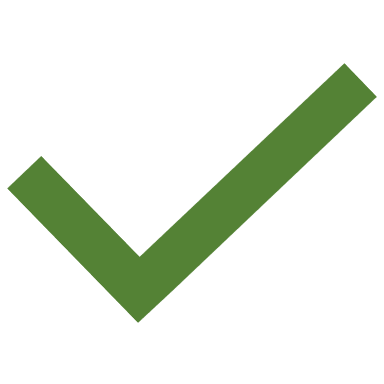 | 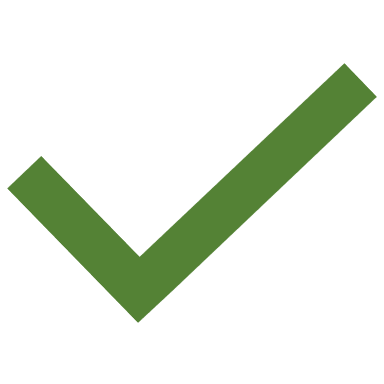 | 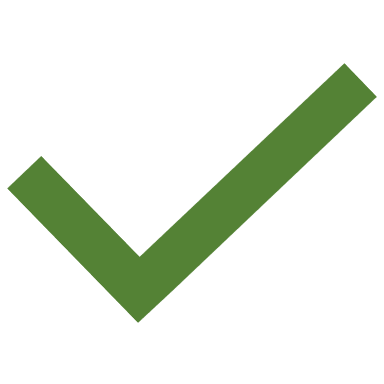 |  |
| **STAT-MI, 2017** | 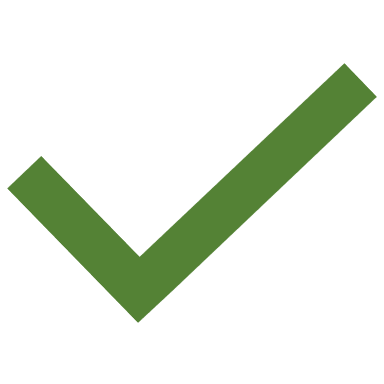 | 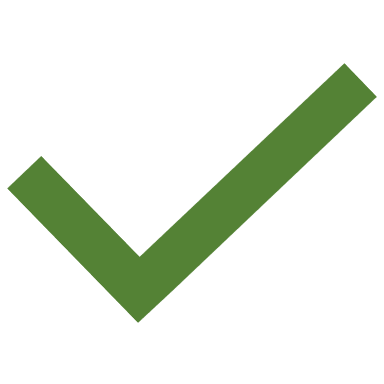 | 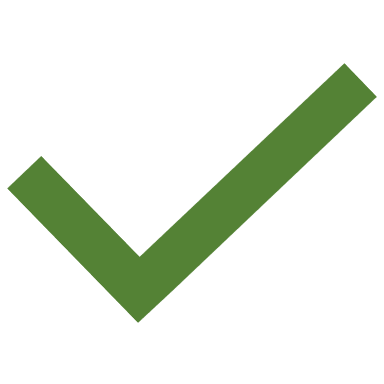 | 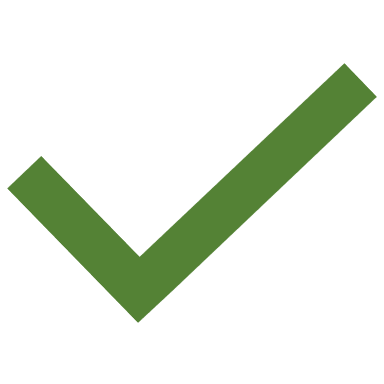 | 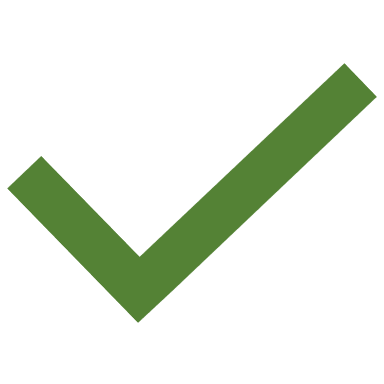 |  |  |  | 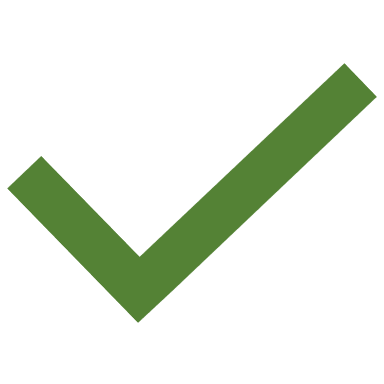 |  |  |  |
| **TETHYS, 2017** |  |  | 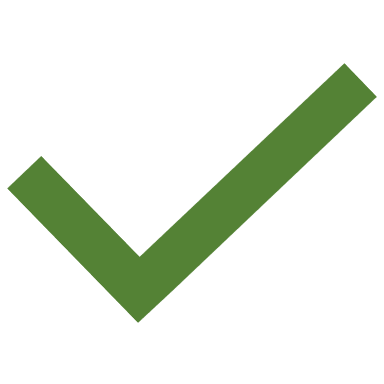 |  | 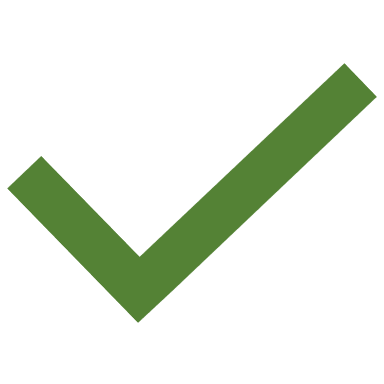 |  |  |  |  |  | 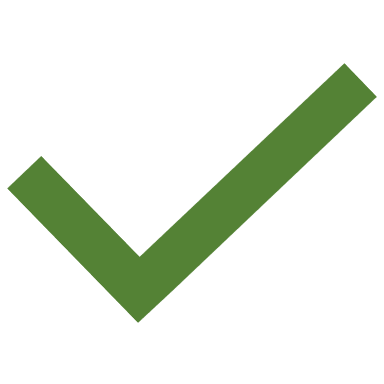 |  |
| **CIRT, 2018** |  |  |  |  |  |  |  |  |  |  |  |  |
| **COLCOT, 2019** |  |  |  |  |  |  |  |  |  |  |  |  |
| **VCUART3, 2019** |  |  |  |  |  |  |  |  |  |  |  |  |
| **LoDoCo-MI, 2019** |  |  |  |  |  |  |  |  |  |  |  |  |
| **LoDoCo2, 2020** |  |  |  |  |  |  |  |  |  |  |  |  |
| **Australian COPS, 2020** |  |  |  |  |  |  |  |  |  |  |  |  |
| **ASSAIL-MI, 2021** |  |  |  |  |  |  |  |  |  |  |  |  |
| **Akrami et al., 2021** |  |  |  |  |  |  |  |  |  |  |  |  |
| **CLEVER-ACS, 2022** |  |  |  |  |  |  |  |  |  |  |  |  |
| **PodCAST-PCI, 2022** |  |  |  |  |  |  |  |  |  |  |  |  |
| **COVERT-MI, 2023** |  |  |  |  |  |  |  |  |  |  |  |  |
| **CLEAR SYNERY (OASIS 9)** |  |  |  |  |  |  |  |  |  |  |  |  |
| **TOTAL** | 20 | 16 | 25 | 15 | 20 | 14 | 11 | 15 | 17 | 5 | 6 | 8 |

Abbreviations: MACE, major adverse cardiovascular events.

**Supplementary Table 7. Available endpoint definitions across included trials.**

| **Study, year** | **MACE** | **Serious adverse events** | **Myocardial infarction** | **Heart failure** | **Revascularization** | **Stroke** |
| --- | --- | --- | --- | --- | --- | --- |
| **COMPLY, 2003** | Death, CHF, cardiogenic shock or stroke. | - | No definition provided. | New or worsening CHF requiring intravenous treatment occuring during index hospitalization or rehospitalization for CHF. | No definition provided. | New focal neurological deficit lasting at least 24 hours with moderate or severe deficit classified as stroke by a physician. |
| **COMMA, 2003** | Death, CHF, cardiogenic shock or stroke. | - | No definition provided. | New or worsening CHF requiring intravenous treatment occuring during index hospitalization or rehospitalization for CHF. | No definition provided. | New focal neurological deficit lasting at least 24 hours with moderate or severe deficit classified as stroke by a physician. |
| **APEX AMI, 2007** | Death, cardiogenic shock or congestive HF. | No definition provided. | Periprocedural: 33% or more CK-MB increase  and accompanying ischemic symptoms.  Recurrent: new Q waves, ≥3x URL CK-MB elevation or ≥2x URL CK-MB and accompanying ischemic symptoms. | New or worsening CHF requiring intravenous treatment occuring during index hospitalization or rehospitalization for CHF. | - | Focal neurological deficit with residual symptoms lasting more than 24 hours. |
| **Piot et al., 2008** | - | Death, HF, acute myocardial infarction, stroke, recurrent  ischemia, the need for repeat revascularization,  renal or hepatic insufficiency, vascular  complications, and bleeding | - | No definition provided | - | - |
| **Ghaffari et al., 2012** | - | - | - | At least two of: new  pulmonary edema by chest X-ray in the absence of a noncardiac  cause, rales over one-third or greater of the lung fields believed to  be due to pulmonary edema, dyspnea with pO2 <80 mm Hg or O2  saturation <90% without known pre-existing lung disease, and  use of loop diuretics. | - | - |
| **VCU-ART2, 2013** | - | No definition provided | No definition provided | ACC/AHA stage C, NYHA III-IV) | No definition provided | - |
| **SELECT-ACS, 2013** | - | All-cause death, nonfatal MI, stroke, hospitalization for ACS, resuscitated cardiac arrest, revascularization, hospitalization fo CHF, renal failure | No definition provided | No definition provided | No definition provided | No definition provided |
| **MRC-ILA, 2014** | Death, stroke and new myocardial infarction | - | No definition provided | - | No definition provided | No definition provided |
| **CIRCUS, 2015** | Death, worsening HF during the initial hospitalization, rehospedalization for HF, or adverse ventricular remodeling. | - | No definition provided | Rehospitalization for CHF where the patient exhibited new or worsening symptoms of HF on presentation, had objective evidence of new or worsening HF and the patient received initiation or intensification of treatment specifically for  HF | - | No definition provided |
| **CYCLE, 2015** | Death, HF or cardiogenic shock | No definition provided | - | Requiring intravenous medications or increased dose of previous therapy and at least two of:  increased dyspnea on exertion, orthopnea, nocturnal dyspnea, increasing peripheral edema,  pulmonary edema,  increasing fatigue/decreasing exercise tolerance,  worsening renal function, elevated jugular venous pressure,  radiological sign of CHF, new evidence of left ventricular systolic dysfunction, new evidence of BNP ≥400 pg/mL, NT-proBNP 1500 pg/ml | - | - |
| **Deftereos et al., 2015** | - | - | - | - | - | - |
| **COLIN, 2016** | - | - | No definition provided | No definition provided | - | - |
| **Kleveland et al., 2016** | Cardiac death, cardiac arrest, myocardial infarction, clinically-driven revascularization, HF. | Cardiac death, cardiac arrest, myocardial infarction, clinically-driven revascularization, HF, pericarditis,serious infection, serious bleeding. | No definition provided | - | - | - |
| **CANTOS, 2017** | Cardiovascular death, nonfatal myocardial infarction or nonfatal stroke. | Deemed important by the investigators. | No definition provided | - | Hospitalization for unstable angina that led to urgent revascularization | No definition provided |
| **STAT-MI, 2017** | All-cause death, recurrent MI, development  of a new arrhythmia, new septal/valve rupture, evidence of  dissection, pericarditis, or tamponade. | No definition provided | No definition provided | - | - | - |
| **TETHYS, 2017** | - | - | No definition provided | - | - | - |
| **CIRT, 2018** | Cardiac death, myocadial infarction or stroke | Adverse events that resulted in death, were life-threatening, led to hospitalization or prolongation of hospitalization,  caused clinically significant incapacity or were deemed to be an important medical event as judged by the investigator. | No efinetion provided | Hospitalization for CHF | Any revascularization | No definition provided |
| **COLCOT, 2019** | Cardiovascular death, cardiac death, myocardial infarction or stroke | Gastrointestinal event, infection, pneumonia, septic shock, hospitalization for HF or cancer development | No definition provided | No defintion given | Urgent hospitalization for angina leading to revascularization | No definition provided |
| **VCUART3, 2019** | All-cause death, myocardial infarction, ischemia-driven revascularization | No definition provided | - | - | - | No definition provided |
| **LoDoCo-MI, 2019** | No definition provided | No definition provided | - | - | - | - |
| **LoDoCo2, 2020** | Cardiovascular death, myocardial infarction or stroke | - | Spontaneous non-periprocedural myocardial infarction | - | Ischemia-driven revascularization | No definition provided |
| **Australian COPS, 2020** | All-cause death, acute coronary syndrome, ischemia-driven revascularization, stroke | Gastrointestinal symptoms, skin rashes, paresthesia, mycalgia, myelosuppression | No definition provided | - | Urgent revascularization | No definition provided |
| **ASSAIL-MI, 2021** | Myocardial infarction, coronary artery bypass graft, chest pain, resuscitated ventricular fibrillation, ventricular tachycardia, ischemic stroke | Infection requiring hospitalization, new malignancy, myocardial infarction, coronary artery bypass graft, chest pain, resuscitated ventricular fibrillation, ventricular tachycardia, ischemic stroke, subarachnoid hemorrhage, worsening renal function, liver-associated events | No definition provided | - | - | No definition provided |
| **Akrami et al., 2021** | Cardiovascular death, recurrent chest pain, resuscitated cardiac arrest, myocardail infarction, stroke | - | No definition provided | Decompensated HF | - | - |
| **CLEVER-ACS, 2022** | - | All-cause death, nonfatal myocardial infarciton, renal failure, bleeding, left ventricular thrombus, infection, diarrhea, oral ulcer, exanthema, atrial fibrillation, other non specified adverse events | No definition provided | - | - | - |
| **PodCAST-PCI, 2022** | No definition provided | - | - | - | - | - |
| **COVERT-MI, 2023** | All-cause death, acute coronary syndorme, heart failure, stroke, sustained ventricular arrythmias, acute kidney injury | - | - | New hospitalization for HF divided into CHF (decision to treat with intravenous diuretics or inotropic or vasodialtor and at least two of: pulmonary edema on chest X-Ray, rales in more than a third of the lung, presence of dyspnea with documented PO2 less than 80 mmHg or oxygen saturation less than 80%, presence of left ventricular ejection fraction less than 50%, incread NT-pro-BNP oor BNP) and worsening of pre-existing HF. | - | Unequivocal signs or symptoms of remaining  neurological deficit, with sudden onset and duration of more than 24 h, confirmed by the  presence of new ischemic cerebral damage upon non invasive imaging (MDCT/ MRI) |
| **CLEAR SYNERGY (OASIS 9)** | Cardiovascular death, myocardial infarction or stroke. | Any adverse event that results in death, is life threatening, requires hospitalization, results in significant disability or a congenital anomaly or is medically important. | According to the fouth universal definition of myocardial infarction. | - | Ischemia-driven revascularization. | acute focal or global* neurological dysfunction caused by brain or retinal vascular injury due to primary  hemorrhage or infarction. Symptoms or signs must persist >24 hours or when acute stroke is present on brain imaging (i.e. if there is an  acute/subacute stroke documented by CT or MRI or at autopsy, the duration of symptoms/signs may be less than 24 hours). |

**Supplementary Table 8.** **Posterior assessment of the results according to GRADE approach.**

| **Subgroup** | **Trials** | **Design** | **RoB** | **Inconsistency** | **Indirectness** | **Imprecision** | **Overall Quality** |
| --- | --- | --- | --- | --- | --- | --- | --- |
| **MACE** | | | | | | | |
| Anakinra | 2 | RCT | Not serious | Serious | Serious | Not serious | Low  ⨁⨁ |
| Canakinumab | 1 | RCT | Not serious | Not serious | Not serious | Not serious | Moderate  ⨁⨁⨁ |
| Colchicine | 8 | RCT | Not serious | Not serious | Not serious | Not serious | High  ⨁⨁⨁⨁ |
| Cyclosporine | 2 | RCT | Not serious | Not serious | Not serious | Not serious | High  ⨁⨁⨁⨁ |
| Everolimus | 0 | - | - | - | - | - | - |
| Inclacumab | 0 | - | - | - | - | - | - |
| Methotrexate | 1 | RCT | Not serious | Not serious | Serious | Not serious | Low  ⨁⨁ |
| Pexelizumab | 3 | RCT | Not serious | Not serious | Not serious | Not serious | High  ⨁⨁⨁⨁ |
| Tocilizumab | 3 | RCT | Serious | Serious | Not serious | Not serious | Low  ⨁⨁ |
| **Overall** | **19** | **RCT** | **Not serious** | **Serious** | **Not serious** | **Not serious** | Moderate  ⨁⨁⨁ |
| **Serious adverse events** | | | | | | | |
| Anakinra | 2 | RCT | Not serious | Not serious | Not serious | Not serious | High  ⨁⨁⨁⨁ |
| Canakinumab | 1 | RCT | Not serious | Not serious | Not serious | Not serious | Moderate  ⨁⨁⨁ |
| Colchicine | 4 | RCT | Not serious | Not serious | Not serious | Not serious | High  ⨁⨁⨁⨁ |
| Cyclosporine | 2 | RCT | Not serious | Not serious | Not serious | Not serious | High  ⨁⨁⨁⨁ |
| Everolimus | 1 | RCT | Not serious | Not serious | Not serious | Not serious | Moderate  ⨁⨁⨁ |
| Inclacumab | 1 | RCT | Not serious | Not serious | Not serious | Not serious | Moderate  ⨁⨁⨁ |
| Methotrexate | 1 | RCT | Not serious | Not serious | Serious | Not serious | Low  ⨁⨁ |
| Pexelizumab | 1 | RCT | Not serious | Not serious | Not serious | Not serious | Moderate  ⨁⨁⨁ |
| Tocilizumab | 3 | RCT | Serious | Not serious | Not serious | Not serious | Moderate  ⨁⨁⨁ |
| **Overall** | **15** | **RCT** | **Not serious** | **Serious** | **Not serious** | **Not serious** | Moderate  ⨁⨁⨁ |
| **All-cause death** | | | | | | | |
| Anakinra | 3 | RCT | Not serious | Not serious | Not serious | Not serious | High  ⨁⨁⨁⨁ |
| Canakinumab | 1 | RCT | Not serious | Not serious | Not serious | Not serious | Moderate  ⨁⨁⨁ |
| Colchicine | 8 | RCT | Not serious | Not serious | Not serious | Not serious | High  ⨁⨁⨁⨁ |
| Cyclosporine | 4 | RCT | Not serious | Serious | Not serious | Not serious | Moderate  ⨁⨁⨁ |
| Everolimus | 1 | RCT | Not serious | Not serious | Not serious | Not serious | Moderate  ⨁⨁⨁ |
| Inclacumab | 1 | RCT | Not serious | Not serious | Not serious | Not serious | Moderate  ⨁⨁⨁ |
| Methotrexate | 2 | RCT | Not serious | Not serious | Not serious | Not serious | High  ⨁⨁⨁⨁ |
| Pexelizumab | 3 | RCT | Not serious | Not serious | Not serious | Not serious | High  ⨁⨁⨁⨁ |
| Tocilizumab | 1 | RCT | Serious | Not serious | Not serious | Not serious | Low  ⨁⨁ |
| **Overall** | **23** | **RCT** | **Not serious** | **Serious** | **Not serious** | **Not serious** | Moderate  ⨁⨁⨁ |
| **Cardiovascular death** | | | | | | | |
| Anakinra | 1 | RCT | Not serious | Not serious | Not serious | Not serious | Moderate  ⨁⨁⨁ |
| Canakinumab | 1 | RCT | Not serious | Not serious | Not serious | Not serious | Moderate  ⨁⨁⨁ |
| Colchicine | 6 | RCT | Not serious | Serious | Not serious | Not serious | Moderate  ⨁⨁⨁ |
| Cyclosporine | 3 | RCT | Not serious | Serious | Not serious | Not serious | Moderate  ⨁⨁⨁ |
| Everolimus | 1 | RCT | Not serious | Not serious | Not serious | Not serious | Moderate  ⨁⨁⨁ |
| Inclacumab | 0 | - | - | - | - | - | - |
| Methotrexate | 1 | RCT | Not serious | Not serious | Serious | Not serious | Low  ⨁⨁ |
| Pexelizumab | 0 | - | - | - | - | - | - |
| Tocilizumab | 2 | RCT | Serious | Not serious | Not serious | Not serious | Moderate  ⨁⨁⨁ |
| **Overall** | **14** | **RCT** | **Not serious** | **Serious** | **Not serious** | **Not serious** | High  ⨁⨁⨁⨁ |
| **Myocardial infarction** | | | | | | | |
| Anakinra | 2 | RCT | Not serious | Not serious | Not serious | Not serious | High  ⨁⨁⨁⨁ |
| Canakinumab | 1 | RCT | Not serious | Not serious | Not serious | Not serious | Moderate  ⨁⨁⨁ |
| Colchicine | 6 | RCT | Not serious | Not serious | Not serious | Not serious | High  ⨁⨁⨁⨁ |
| Cyclosporine | 1 | RCT | Not serious | Not serious | Not serious | Not serious | Moderate  ⨁⨁⨁ |
| Everolimus | 1 | RCT | Not serious | Not serious | Not serious | Not serious | Moderate  ⨁⨁⨁ |
| Inclacumab | 1 | RCT | Not serious | Not serious | Not serious | Not serious | Moderate  ⨁⨁⨁ |
| Methotrexate | 1 | RCT | Not serious | Not serious | Not serious | Not serious | Moderate  ⨁⨁⨁ |
| Pexelizumab | 3 | RCT | Not serious | Serious | Not serious | Not serious | Moderate  ⨁⨁⨁ |
| Tocilizumab | 3 | RCT | Serious | Not serious | Not serious | Not serious | Moderate  ⨁⨁⨁ |
| **Overall** | **19** | **RCT** | **Not serious** | **Serious** | **Not serious** | **Not serious** | Moderate  ⨁⨁⨁ |
| **Heart failure** | | | | | | | |
| Anakinra | 1 | RCT | Not serious | Not serious | Not serious | Not serious | Moderate  ⨁⨁⨁ |
| Canakinumab | 0 | - | - | - | - | - | **-** |
| Colchicine | 4 | RCT | Not serious | Serious | Not serious | Not serious | Moderate  ⨁⨁⨁ |
| Cyclosporine | 4 | RCT | Not serious | Not serious | Not serious | Not serious | High  ⨁⨁⨁⨁ |
| Everolimus | 0 | - | - | - | - | - | **-** |
| Inclacumab | 1 | RCT | Not serious | Not serious | Not serious | Not serious | Moderate  ⨁⨁⨁ |
| Methotrexate | 1 | RCT | Not serious | Not serious | Serious | Not serious | Low  ⨁⨁ |
| Pexelizumab | 3 | RCT | Not serious | Not serious | Not serious | Not serious | High  ⨁⨁⨁⨁ |
| Tocilizumab | 0 | - | - | - | - | - | **-** |
| **Overall** | **14** | **RCT** | **Not serious** | **Serious** | **Not serious** | **Not serious** | Moderate  ⨁⨁⨁ |
| **Revascularization** | | | | | | | |
| Anakinra | 2 | RCT | Not serious | Not serious | Not serious | Not serious | High  ⨁⨁⨁⨁ |
| Canakinumab | 1 | RCT | Not serious | Not serious | Not serious | Not serious | Moderate  ⨁⨁⨁ |
| Colchicine | 4 | RCT | Not serious | Not serious | Not serious | Not serious | High  ⨁⨁⨁⨁ |
| Cyclosporine | 0 | - | - | - | - | - | - |
| Everolimus | 0 | - | - | - | - | - | - |
| Inclacumab | 1 | RCT | Not serious | Not serious | Not serious | Not serious | Moderate  ⨁⨁⨁ |
| Methotrexate | 1 | RCT | Not serious | Not serious | Serious | Not serious | Low  ⨁⨁ |
| Pexelizumab | 2 | RCT | Not serious | Not serious | Not serious | Not serious | High  ⨁⨁⨁⨁ |
| Tocilizumab | 0 | - | - | - | - | - | - |
| **Overall** | **10** | **RCT** | **Not serious** | **Not serious** | **Not serious** | **Not serious** | High  ⨁⨁⨁⨁ |
| **Stroke** | | | | | | | |
| Anakinra | 2 | RCT | Not serious | Not serious | Not serious | Not serious | High  ⨁⨁⨁⨁ |
| Canakinumab | 1 | RCT | Not serious | Not serious | Not serious | Not serious | Moderate  ⨁⨁⨁ |
| Colchicine | 5 | RCT | Not serious | Not serious | Not serious | Not serious | High  ⨁⨁⨁⨁ |
| Cyclosporine | 1 | RCT | Not serious | Not serious | Not serious | Not serious | Moderate  ⨁⨁⨁ |
| Everolimus | 0 | - | - | - | - | - | - |
| Inclacumab | 1 | RCT | Not serious | Not serious | Not serious | Not serious | Moderate  ⨁⨁⨁ |
| Methotrexate | 1 | RCT | Not serious | Not serious | Serious | Not serious | Low  ⨁⨁ |
| Pexelizumab | 3 | RCT | Not serious | Not serious | Not serious | Not serious | High  ⨁⨁⨁⨁ |
| Tocilizumab | 1 | RCT | Not serious | Not serious | Not serious | Not serious | Moderate  ⨁⨁⨁ |
| **Overall** | **14** | **RCT** | **Not serious** | **Not serious** | **Not serious** | **Not serious** | High  ⨁⨁⨁⨁ |
| **Serious infection or sepsis** | | | | | | | |
| Anakinra | 3 | RCT | Not serious | Not serious | Not serious | Not serious | High  ⨁⨁⨁⨁ |
| Canakinumab | 1 | RCT | Not serious | Not serious | Not serious | Not serious | Moderate  ⨁⨁⨁ |
| Colchicine | 4 | RCT | Not serious | Not serious | Not serious | Not serious | High  ⨁⨁⨁⨁ |
| Cyclosporine | 0 | - | - | - | - | - | **-** |
| Everolimus | 1 | RCT | Not serious | Not serious | Not serious | Not serious | Moderate  ⨁⨁⨁ |
| Inclacumab | 1 | RCT | Not serious | Not serious | Not serious | Not serious | Moderate  ⨁⨁⨁ |
| Methotrexate | 1 | RCT | Not serious | Not serious | Serious | Not serious | Low  ⨁⨁ |
| Pexelizumab | 3 | RCT | Not serious | Not serious | Not serious | Not serious | High  ⨁⨁⨁⨁ |
| Tocilizumab | 3 | RCT | Not serious | Not serious | Not serious | Not serious | High  ⨁⨁⨁⨁ |
| **Overall** | **16** | **RCT** | **Not serious** | **Not serious** | **Not serious** | **Not serious** | High  ⨁⨁⨁⨁ |
| **Cancer development** | | | | | | | |
| Anakinra | 0 | - | - | - | - | - | **-** |
| Canakinumab | 1 | RCT | Not serious | Not serious | Not serious | Not serious | Moderate  ⨁⨁⨁ |
| Colchicine | 2 | RCT | Not serious | Not serious | Not serious | Not serious | High  ⨁⨁⨁⨁ |
| Cyclosporine | 0 | - | - | - | - | - | **-** |
| Everolimus | 0 | - | - | - | - | - | **-** |
| Inclacumab | 0 | - | - | - | - | - | **-** |
| Methotrexate | 1 | RCT | Not serious | Not serious | Not serious | Not serious | Moderate  ⨁⨁⨁ |
| Pexelizumab | 0 | - | - | - | - | - | **-** |
| Tocilizumab | 1 | RCT | Not serious | Not serious | Not serious | Not serious | Moderate  ⨁⨁⨁ |
| **Overall** | **5** | **RCT** | **Not serious** | **Not serious** | **Not serious** | **Not serious** | High  ⨁⨁⨁⨁ |
| **Pneumonia** | | | | | | | |
| Anakinra | 1 | RCT | Not serious | Not serious | Not serious | Not serious | Moderate  ⨁⨁⨁ |
| Canakinumab | 1 | RCT | Not serious | Not serious | Not serious | Not serious | Moderate  ⨁⨁⨁ |
| Colchicine | 3 | RCT | Not serious | Serious | Not serious | Not serious | Moderate  ⨁⨁⨁ |
| Cyclosporine | 0 | - | - | - | - | - | **-** |
| Everolimus | 0 | - | - | - | - | - | **-** |
| Inclacumab | 0 | - | - | - | - | - | **-** |
| Methotrexate | 1 | RCT | Not serious | Not serious | Not serious | Not serious | Moderate  ⨁⨁⨁ |
| Pexelizumab | 0 | - | - | - | - | - | **-** |
| Tocilizumab | 0 | - | - | - | - | - | **-** |
| **Overall** | **6** | **RCT** | **Not serious** | **Serious** | **Not serious** | **Not serious** | Moderate  ⨁⨁⨁ |
| **Gastrointestinal adverse events** | | | | | | | |
| Anakinra | 0 | - | - | - | - | - | - |
| Canakinumab | 0 | - | - | - | - | - | - |
| Colchicine | 7 | RCT | Not serious | Not serious | Not serious | Not serious | High  ⨁⨁⨁⨁ |
| Cyclosporine | 0 | - | - | - | - | - | - |
| Everolimus | 1 | RCT | Not serious | Not serious | Not serious | Not serious | Moderate  ⨁⨁⨁ |
| Inclacumab | 0 | - | - | - | - | - | - |
| Methotrexate | 2 | RCT | Not serious | Serious | Serious | Not serious | Low  ⨁⨁ |
| Pexelizumab | 0 | - | - | - | - | - | - |
| Tocilizumab | 0 | - | - | - | - | - | - |
| **Overall** | **9** | **RCT** | **Not serious** | **Not serious** | **Not serious** | **Not serious** | High  ⨁⨁⨁⨁ |

Evidence coming from one single trial was downgraded even in case of absence of serious signs of low quality.
Abbreviations: MACE, major adverse cardiovascular events.

**Supplementary Table 9. Interaction analysis for clinical presentation and timing of administration.**

| **Drug administered** | **Comparisons** | **IRR (95% CI)** | **Heterogeneity** |
| --- | --- | --- | --- |
| **Interaction for clinical presentation** | | | |
| **MACE** | | | |
| Acute MI | 16 | 0.96 (0.88 – 1.05) | τ^2^<0.001; I^2^: 41% |
| Stabilized MI | 4 | **0.88 (0.81 – 0.96)** | τ^2^<0.001; I^2^: 28% |
| **Overall** | **20** | **0.92 (0.86 – 0.98)** | **τ^2^ <0.001; I^2^: 39%** |
| **P for interaction: 0.170** |  |  |  |
| **Serious adverse events** | | | |
| Acute MI | 13 | 0.96 (0.85 – 1.08) | τ^2^: 0.000; I^2^: 0% |
| Stabilized MI | 3 | 0.99 (0.94 – 1.05) | τ^2^: 0.000; I^2^: 0% |
| **Overall** | **16** | **0.99 (0.94 – 1.04)** | **τ^2^: 0.000; I^2^: 0%** |
| **P for interaction: 0.549** |  |  |  |
| **All-cause death** | | | |
| Acute MI | 20 | 1.2 (0.88 – 1.17) | τ^2^: 0.002; I^2^: 0% |
| Stabilized MI | 4 | 1.02 (0.88 – 1.18) | τ^2^: 0.006; I^2^: 8% |
| **Overall** | **24** | **1.01 (0.92 – 1.10)** | **τ^2^: 0.002; I^2^: 0%** |
| **P for interaction: 0.957** |  |  |  |
| **Cardiovascular death** | | | |
| Acute MI | 11 | 1.09 (0.87 – 1.36) | τ^2^: 0.000; I^2^: 0% |
| Stabilized MI | 4 | 0.90 (0.77 – 1.05) | τ^2^: 0.000; I^2^: 0% |
| **Overall** | **15** | **0.96 (0.84 – 1.09)** | **τ^2^: 0.000; I^2^: 0%** |
| **P for interaction: 0.175** |  |  |  |
| **Myocardial infarction** | | | |
| Acute MI | 16 | 1.02 (0.80 – 1.29) | τ^2^: 0.002; I^2^: 15% |
| Stabilized MI | 4 | 0.86 (0.78 – 0.96) | τ^2^<0.001; I^2^: 0% |
| **Overall** | **20** | **0.91 (0.81 – 1.02)** | **τ^2^: 0.007; I^2^: 18%** |
| **P for interaction: 0.214** |  |  |  |
| **Heart failure** | | | |
| Acute MI | 12 | 0.96 (0.83 – 1.12) | τ^2^: 0.000; I^2^: 0% |
| Stabilized MI | 2 | 1.09 (0.69 – 1.74) | τ^2^: 0.051; I^2^: 42% |
| **Overall** | **14** | **0.97 (0.85 – 1.12)** | **τ^2^: 0.000; I^2^: 0%** |
| **P for interaction: 0.610** |  |  |  |
| **Revascularization** | | | |
| Acute MI | 7 | 1.02 (0.87 – 1.19) | τ^2^: 0.000; I^2^: 17% |
| Stabilized MI | 4 | 0.70 (0.60 – 0.82) | τ^2^ <0.001; I^2^: 9% |
| **Overall** | **11** | **0.83 (0.69 – 1.00)** | **τ^2^: 0.045; I^2^: 54%** |
| **P for interaction <0.001** |  |  |  |
| **Stroke** | | | |
| Acute MI | 11 | 1.02 (0.78 – 1.32) | τ^2^: 0.000; I^2^: 0% |
| Stabilized MI | 4 | 0.76 (0.52 – 1.10) | τ^2^: 0.070; I^2^: 54% |
| **Overall** | **15** | **0.91 (0.77 – 1.07)** | **τ^2^ <0.000; I^2^: 5%** |
| **P for interaction: 0.201** |  |  |  |
| **Serious infection or sepsis** | | | |
| Acute MI | 13 | 0.89 (0.74 – 1.07) | τ^2^: 0.000; I^2^: 0% |
| Stabilized MI | 4 | 1.05 (0.95 – 1.17) | τ^2^: 0.001; I^2^: 15% |
| **Overall** | **17** | **1.00 (0.90 – 1.11)** | **τ^2^: 0.003; I^2^: 0%** |
| **P for interaction: 0.118** |  |  |  |
| **Cancer development** | | | |
| Acute MI | 1 | 4.54 (0.22 – 94.50) | - |
| Stabilized MI | 4 | 0.97 (0.87 – 1.09) | τ^2^: 0.000; I^2^: 0% |
| **Overall** | **5** | **0.97 (0.87 – 1.09)** | **τ^2^: 0.000; I^2^: 0%** |
| **P for interaction: 0.603** |  |  |  |
| **Pneumonia** | | | |
| Acute MI | 3 | 0.86 (0.14 – 5.48) | τ^2^: 0.096; I^2^: 1% |
| Stabilized MI | 3 | 1.14 (0.72 – 1.79) | τ^2^: 0.109; I^2^: 62% |
| **Overall** | **6** | **1.11 (0.74 – 1.66)** | **τ^2^: 0.084; I^2^: 32%** |
| **P for interaction: 0.776** |  |  |  |
| **Gastrointestinal adverse events** | | | |
| Acute MI | 7 | 1.19 (0.94 – 1.50) | τ^2^<0.001; I^2^: 52% |
| Stabilized MI | 3 | 1.22 (1.06 – 1.40) | τ^2^: 0.000; I^2^: 0% |
| **Overall** | **10** | **1.21 (1.07 – 1.36)** | **τ^2^ <0.001; I^2^: 31%** |
| **P for interaction: 0.872** |  |  |  |
| **Interaction for timing of administration** | | | |
| **MACE** | | | |
| Early | 5 | 0.69 (0.46 – 1.03) | τ^2^: 0.162; I^2^: 61% |
| Late | 3 | 0.88 (0.76 – 1.02) | τ^2^: 0.009; I^2^: 51% |
| Immediate | 12 | 1.00 (0.90 – 1.11) | τ^2^ <0.001; I^2^: 8% |
| **Overall** | **20** | **0.92 (0.86 – 0.98)** | **τ^2^ <0.001; I^2^: 39%** |
| **P for interaction: 0.113** |  |  |  |
| **Serious adverse events** | | | |
| Early | 6 | 0.94 (0.85 – 1.04) | τ^2^: 0.000; I^2^: 0% |
| Late | 2 | 1.00 (0.94 – 1.06) | τ^2^: 0.000; I^2^: 0% |
| Immediate | 8 | 0.96 (0.85 – 1.09) | τ^2^:0.013; I^2^: 0% |
| **Overall** | **16** | **0.99 (0.94 – 1.04)** | **τ^2^: 0.000; I^2^: 0%** |
| **P for interaction: 0.558** |  |  |  |
| **All-cause death** | | | |
| Early | 8 | 0.95 (0.78 – 1.14) | τ^2^: 0.000; I^2^: 0% |
| Late | 3 | 1.04 (0.87 – 2.28) | τ^2^: 0.012; I^2^: 39% |
| Immediate | 13 | 1.07 (0.90 – 1.28) | τ^2^:0.000; I^2^: 0% |
| **Overall** | **24** | **1.01 (0.92 – 1.10)** | **τ^2^: 0.002; I^2^: 0%** |
| **P for interaction: 0.605** |  |  |  |
| **Cardiovascular death** | | | |
| Early | 7 | 1.02 (0.70 – 2.21) | τ^2^: 0.040; I^2^: 0% |
| Late | 3 | 0.90 (0.77 – 1.06) | τ^2^: 0.000; I^2^: 0% |
| Immediate | 5 | 1.24 (0.70 – 2.21) | τ^2^:0.040; I^2^: 0% |
| **Overall** | **15** | **0.96 (0.84 – 1.09)** | **τ^2^: 0.000; I^2^: 0%** |
| **P for interaction: 0.453** |  |  |  |
| **Myocardial infarction** | | | |
| Early | 7 | 0.91 (0.75 – 1.10) | τ^2^: 0.000; I^2^: 0% |
| Late | 3 | 0.86 (0.76 – 0.96) | τ^2^<0.000; I^2^: 29% |
| Immediate | 10 | 1.10 (0.76 – 1.58) | τ^2^:0.048; I^2^: 33% |
| **Overall** | **20** | **0.91 (0.81 – 1.02)** | **τ^2^: 0.007; I^2^: 18%** |
| **P for interaction: 0.415** |  |  |  |
| **Heart failure** | | | |
| Early | 4 | 0.93 (0.83 – 1.13) | τ^2^: 0.310; I^2^: 0% |
| Late | 1 | 0.91 (0.61 – 1.34) | - |
| Immediate | 9 | 0.97 (0.83 – 1.13) | τ^2^<0.001; I^2^: 0% |
| **Overall** | **14** | **0.97 (0.85 – 1.12)** | **τ^2^: 0.000; I^2^: 0%** |
| **P for interaction: 0.951** |  |  |  |
| **Revascularization** | | | |
| Early | 4 | 0.62 (0.33 – 1.16) | τ^2^ <0.001; I^2^: 0% |
| Late | 3 | 0.73 (0.62 – 0.86) | τ^2^: 0.000; I^2^: 0% |
| Immediate | 4 | 1.09 (0.87 – 1.37) | τ^2^<0.001; I^2^: 0% |
| **Overall** | **11** | **0.83 (0.69 – 1.00)** | **τ^2^: 0.045; I^2^: 54%** |
| **P for interaction: 0.012** |  |  |  |
| **Stroke** | | | |
| Early | 3 | 0.54 (0.19 – 1.53) | τ^2^: 0.604; I^2^: 78% |
| Late | 3 | 0.90 (0.72 – 1.11) | τ^2^: 0.000; I^2^: 0% |
| Immediate | 9 | 0.95 (0.64 – 1.40) | τ^2^:0.018; I^2^: 0% |
| **Overall** | **15** | **0.91 (0.77 – 1.07)** | **τ^2^ <0.000; I^2^: 5%** |
| **P for interaction: 0.606** |  |  |  |
| **Serious infection or sepsis** | | | |
| Early | 5 | 1.06 (0.74 – 1.53) | τ^2^: 0.045; I^2^: 2% |
| Late | 3 | 1.03 (0.91 – 1.16) | τ^2^: 0.003; I^2^: 5% |
| Immediate | 9 | 0.89 (0.70 – 1.14) | τ^2^:0.000; I^2^: 0% |
| **Overall** | **17** | **1.00 (0.90 – 1.11)** | **τ^2^: 0.003; I^2^: 0%** |
| **P for interaction: 0.572** |  |  |  |
| **Cancer development** | | | |
| Early | 1 | 0.94 (0.62 – 1.43) | - |
| Late | 3 | 0.98 (0.86 – 1.10) | τ^2^: 0.000; I^2^: 0% |
| Immediate | 1 | 4.54 (0.22 – 94.51) | - |
| **Overall** | **5** | **0.97 (0.87 – 1.09)** | **τ^2^: 0.000; I^2^: 0%** |
| **P for interaction: 0.603** |  |  |  |
| **Pneumonia** | | | |
| Early | 3 | 2.20 (1.06 – 4.60) | τ^2^<0.001; I^2^: 0% |
| Late | 2 | 1.00 (0.81 – 1.22) | τ^2^: 0.013; I^2^: 5% |
| Immediate | 1 | 0.32 (0.01 – 7.80) | - |
| **Overall** | **6** | **1.11 (0.74 – 1.66)** | **τ^2^: 0.084; I^2^: 32%** |
| **P for interaction: 0.096** |  |  |  |
| **Gastrointestinal adverse events** | | | |
| Early | 7 | 1.22 (0.99 – 1.51) | τ^2^<0.001; I^2^: 51% |
| Late | 2 | 1.21 (1.05 – 1.40) | τ^2^: 0.000; I^2^: 0% |
| Immediate | 1 | 0.95 (0.33 – 2.72) | - |
| **Overall** | **10** | **1.21 (1.07 – 1.36)** | **τ^2^ <0.001; I^2^: 31%** |
| **P for interaction: 0.902** |  |  |  |

Light blue cells highlight significant reduction with treatment, while light red cells highlight significant increase with treatment. Significant p-values for interaction are shown in red.
Abbreviations: CI, confidence interval; IRR, incidence rate ratio; MACE, major adverse cardiovascular events.

**Supplementary Table 10.** **Indirect comparisons of anti-inflammatory drugs.**

| **MACE** | | | | | | | | | |
| --- | --- | --- | --- | --- | --- | --- | --- | --- | --- |
|  | Anakinra | Canakinumab | Colchicine | Cyclosporine | Everolimus | Inclacumab | Methotrexate | Pexelizumab | Tocilizumab |
| Anakinra |  | 1.68  (0.72-3.92) | 1.85  (0.80-4.27) | 1.47  (0.63-3.45) | - | - | 1.46  (0.62-3.48) | 1.50  (0.65-3.50) | 1.34  (0.46-3.84) |
| Canakinumab | 0.60  (0.26-1.39) |  | 1.10  (0.87-1.41) | 0.87  (0.65-1.18) | - | - | 0.87  (0.62-1.22) | 0.90  (0.69-1.17) | 0.80  (0.40-1.59) |
| Colchicine | 0.54  (0.23-1.24) | 0.91  (0.71-1.15) |  | 0.79  (0.61-1.03) | - | - | 0.79  (0.58-1.07) | 0.81  (0.65-1.02) | 0.72  (0.37-1.42) |
| Cyclosporine | 0.68  (0.29-1.60) | 1.14  (0.85-1.54) | 1.26  (0.97-1.65) |  | - | - | 1.00  (0.70-1.42) | 1.02  (0.77-1.37) |  |
| Everolimus | - | - | - | - |  | - | - | - | - |
| Inclacumab | - | - | - | - | - |  | - | - | - |
| Methotrexate | 0.68  (0.29-1.63) | 1.15  (0.82-1.61) | 1.27  (0.93-1.72) | 1.00  (0.71-1.43) | - | - |  | 1.03  (0.74-1.42) | 0.91  (0.45-1.87) |
| Pexelizumab | 0.66  (0.29-1.54) | 1.12  (0.85-1.46) | 1.23  (0.98-1.55) | 0.98  (0.73-1.3) | - | - | 0.97  (0.70-1.35) |  | 0.89  (0.45-1.76) |
| Tocilizumab | 0.75  (0.26-2.15) | 1.26  (0.63-2.51) | 1.39  (0.71-2.73) | 1.10  (0.55-2.21) | - | - | 1.10  (0.54-2.24) | 1.13  (0.57-2.24) |  |
| Heterogeneity: τ^2^=0.007; I^2^: 42%. | | | | | | | | | |
| **Serious adverse events** | | | | | | | | | |
|  | Anakinra | Canakinumab | Colchicine | Cyclosporine | Everolimus | Inclacumab | Methotrexate | Pexelizumab | Tocilizumab |
| Anakinra |  | 1.00  (0.38-2.60) | 1.06  (0.41-2.77) | 1.83  (0.38-8.77) | 0.62  (0.19-2.07) | 0.73  (0.26-2.06) | 0.95  (0.36-2.49) | 1.10  (0.40-3.03) | 0.99  (0.32-3.04) |
| Canakinumab | 1.00  (0.38-2.61) |  | 1.06  (0.94-1.20) | 1.83  (0.52-6.37) | 0.62  (0.30-1.29) | 0.73  (0.48-1.10) | 0.95  (0.83-1.09) | 1.10  (0.78-1.55) | 0.99  (0.54-1.80) |
| Colchicine | 0.94  (0.36-2.46) | 0.94  (0.83-1.06) |  | 1.72  (0.49-6.01) | 0.59  (0.28-1.22) | 0.69  (0.45-1.04) | 0.90  (0.77-1.05) | 1.04  (0.73-1.47) | 0.93  (0.51-1.70) |
| Cyclosporine | 0.55  (0.11-2.63) | 0.55  (0.16-1.90) | 0.58  (0.17-2.03) |  | 0.34  (0.08-1.44) | 0.40  (0.11-1.48) | 0.52  (0.15-1.82) | 0.60  (0.17-2.19) | 0.54  (0.14-2.15) |
| Everolimus | 1.60  (0.48-5.31) | 1.60  (0.77-3.31) | 1.70  (0.82-3.52) | 2.93  (0.69-12.35) |  | 1.17  (0.51-2.67) | 1.53  (0.73-3.17) | 1.76  (0.80-3.91) | 1.58  (0.62-4.03) |
| Inclacumab | 1.37  (0.49-3.87) | 1.37  (0.91-2.07) | 1.45  (0.96-2.21) | 2.50  (0.68-9.28) | 0.86  (0.37-1.96) |  | 1.31  (0.86-1.99) | 1.51  (0.89-2.55) | 1.36  (0.66-2.78) |
| Methotrexate | 1.05  (0.40-2.75) | 1.05  (0.92-1.20) | 1.11  (0.95-1.30) | 1.92  (0.55-6.70) | 0.66  (0.32-1.36) | 0.77  (0.50-1.17) |  | 1.16  (0.81-1.65) | 1.04  (0.57-1.90) |
| Pexelizumab | 0.91  (0.33-2.50) | 0.91  (0.65-1.28) | 0.96(0.68-1.37) | 1.66  (0.46-6.03) | 0.57  (0.26-1.26) | 0.66  (0.39-1.12) | 0.87  (0.61-1.23) |  | 0.90  (0.45-1.77) |
| Tocilizumab | 1.01  (0.33-3.11) | 1.01  (0.56-1.84) | 1.07  (0.59-1.96) | 1.85  (0.46-7.34) | 0.63  (0.25-1.61) | 0.74  (0.36-1.51) | 0.96  (0.53-1.76) | 1.11  (0.56-2.2) |  |
| Heterogeneity: τ^2^<0.001; I^2^: 0%. | | | | | | | | | |
| **All-cause death** | | | | | | | | | |
|  | Anakinra | Canakinumab | Colchicine | Cyclosporine | Everolimus | Inclacumab | Methotrexate | Pexelizumab | Tocilizumab |
| Anakinra |  | 1.16  (0.3-4.5) | 1.05  (0.27-4.06) | 0.94  (0.23-3.81) | 1.09  (0.02-68.72) | 0.17  (0.01-4.07) | 0.92  (0.23-3.65) | 1.08  (0.28-4.21) | 0.75  (0.01-47.25) |
| Canakinumab | 0.86  (0.22-3.33) |  | 0.91  (0.67-1.23) | 0.80  (0.49-1.31) | 0.94  (0.02-47.69) | 0.15  (0.01-2.63) | 0.79  (0.52-1.19) | 0.93  (0.67-1.30) | 0.64  (0.01-32.79) |
| Colchicine | 0.95  (0.25-3.66) | 1.10  (0.82-1.49) |  | 0.89  (0.55-1.43) | 1.03  (0.02-52.56) | 0.16  (0.01-2.90) | 0.87  (0.59-1.30) | 1.03  (0.75-1.41) | 0.71  (0.01-36.13) |
| Cyclosporine | 1.07  (0.26-4.34) | 1.24  (0.76-2.02) | 1.13  (0.7-1.81) |  | 1.16  (0.02-60.28) | 0.18  (0.01-3.35) | 0.98  (0.57-1.70) | 1.16  (0.71-1.90) | 0.80  (0.02-41.44) |
| Everolimus | 0.92  (0.01-57.99) | 1.07  (0.02-54.45) | 0.97  (0.02-49.27) | 0.86  (0.02-44.58) |  | 0.16  (0.00-20.32) | 0.85  (0.02-43.43) | 1.00  (0.02-50.81) | 0.69  (0.00-176.86) |
| Inclacumab | 5.89  (0.25-141.06) | 6.85  (0.38-123.39) | 6.20  (0.34-111.6) | 5.51  (0.30-101.65) | 6.41  (0.05-834.66) |  | 5.42  (0.30-98.73) | 6.38  (0.35-115.19) | 4.41  (0.03-573.83) |
| Methotrexate | 1.09  (0.27-4.31) | 1.26  (0.84-1.91) | 1.15  (0.77-1.70) | 1.02  (0.59-1.76) | 1.18  (0.02-60.79) | 0.18  (0.01-3.36) |  | 1.18  (0.78-1.79) | 0.81  (0.02-41.79) |
| Pexelizumab | 0.92  (0.24-3.58) | 1.07  (0.77-1.49) | 0.97  (0.71-1.33) | 0.86  (0.53-1.42) | 1.00  (0.02-51.19) | 0.16  (0.01-2.83) | 0.85  (0.56-1.29) |  | 0.69  (0.01-35.20) |
| Tocilizumab | 1.34  (0.02-84.34) | 1.55  (0.03-79.19) | 1.41  (0.03-71.66) | 1.25  (0.02-64.84) | 1.45  (0.01-374.18) | 0.23  (0.00-29.56) | 1.23  (0.02-63.18) | 1.45  (0.03-73.91) |  |
| Heterogeneity: τ^2^: 0.009; I^2^: 5%. | | | | | | | | | |
| **Cardiovascular death** | | | | | | | | | |
|  | Anakinra | Canakinumab | Colchicine | Cyclosporine | Everolimus | Inclacumab | Methotrexate | Pexelizumab | Tocilizumab |
| Anakinra |  | 0.38  (0.02-9.43) | 0.34  (0.01-8.29) | 0.29  (0.01-7.35) | 0.33  (0.00-52.56) | - | 0.29  (0.01-7.36) | - | 0.15  (0.00-8.51) |
| Canakinumab | 2.62  (0.11-64.59) |  | 0.88  (0.66-1.16) | 0.75  (0.44-1.29) | 0.87  (0.02-44.16) | - | 0.76  (0.49-1.20) | - | 0.39  (0.03-4.67) |
| Colchicine | 2.98  (0.12-73.76) | 1.14  (0.86-1.51) |  | 0.86  (0.50-1.49) | 0.99  (0.02-50.41) | - | 0.87  (0.55-1.38) | - | 0.44  (0.04-5.33) |
| Cyclosporine | 3.47  (0.14-88.74) | 1.33  (0.78-2.27) | 1.16  (0.67-2.02) |  | 1.16  (0.02-60.29) | - | 1.01  (0.53-1.95) | - | 0.52  (0.04-6.48) |
| Everolimus | 3.00  (0.02-473.06) | 1.15  (0.02-58) | 1.01  (0.02-50.99) | 0.86  (0.02-44.95) |  | - | 0.88  (0.02-45.10) | - | 0.45  (0-46.02) |
| Inclacumab | - | - | - | - | - |  | - | - | - |
| Methotrexate | 3.42  (0.14-86.28) | 1.31  (0.84-2.05) | 1.15  (0.72-1.82) | 0.99  (0.51-1.89) | 1.14  (0.02-58.77) | - |  | - | 0.51  (0.04-6.27) |
| Pexelizumab | - | - | - | - | - | - | - |  | - |
| Tocilizumab | 6.74  (0.12-386.12) | 2.57  (0.21-30.91) | 2.26  (0.19-27.21) | 1.94  (0.15-24.35) | 2.25  (0.02-232.10) | - | 1.97  (0.16-24.28) | - |  |
| Heterogeneity: τ^2^ <0.000; I^2^: 0%. | | | | | | | | | |
| **Myocardial infarction** | | | | | | | | | |
|  | Anakinra | Canakinumab | Colchicine | Cyclosporine | Everolimus | Inclacumab | Methotrexate | Pexelizumab | Tocilizumab |
| Anakinra |  | 4.27  (1.05-17.35) | 4.35(1.07-17.7) | 6.08(1.2-30.74) | 8.66  (0.26-284.16) | 1.35  (0.17-10.5) | 3.64  (0.88-15.03) | 3.07  (0.74-12.77) | 7.71  (1.06-56.22) |
| Canakinumab | 0.23  (0.06-0.95) |  | 1.02  (0.82-1.26) | 1.42  (0.61-3.29) | 2.03  (0.08-49.92) | 0.32  (0.07-1.43) | 0.85  (0.63-1.15) | 0.72  (0.52-0.99) | 1.80  (0.43-7.49) |
| Colchicine | 0.23  (0.06-0.94) | 0.98  (0.79-1.22) |  | 1.40  (0.60-3.24) | 1.99  (0.08-49.07) | 0.31  (0.07-1.41) | 0.84  (0.62-1.13) | 0.71  (0.51-0.98) | 1.77  (0.43-7.37) |
| Cyclosporine | 0.16  (0.03-0.83) | 0.70  (0.30-1.63) | 0.72  (0.31-1.66) |  | 1.42  (0.05-38.84) | 0.22  (0.04-1.24) | 0.60  (0.25-1.42) | 0.51  (0.21-1.21) | 1.27  (0.25-6.53) |
| Everolimus | 0.12  (0.00-3.79) | 0.49  (0.02-12.15) | 0.50  (0.02-12.38) | 0.70  (0.03-19.14) |  | 0.16  (0.00-5.35) | 0.42  (0.02-10.43) | 0.35  (0.01-8.83) | 0.89  (0.03-29.47) |
| Inclacumab | 0.74  (0.10-5.78) | 3.17  (0.70-14.39) | 3.22  (0.71-14.67) | 4.51  (0.81-25.13) | 6.42  (0.19-220.77) |  | 2.70  (0.59-12.45) | 2.28  (0.49-10.57) | 5.72  (0.72-45.18) |
| Methotrexate | 0.27  (0.07-1.14) | 1.17  (0.87-1.58) | 1.19  (0.88-1.62) | 1.67  (0.70-3.97) | 2.38  (0.10-59.01) | 0.37  (0.08-1.71) |  | 0.84  (0.57-1.25) | 2.12  (0.5-8.93) |
| Pexelizumab | 0.33  (0.08-1.35) | 1.39  (1.01-1.92) | 1.41  (1.02-1.97) | 1.98  (0.82-4.75) | 2.82  (0.11-70.07) | 0.44  (0.09-2.03) | 1.18  (0.80-1.75) |  | 2.51  (0.59-10.64) |
| Tocilizumab | 0.13  (0.02-0.95) | 0.55  (0.13-2.30) | 0.56  (0.14-2.35) | 0.79  (0.15-4.06) | 1.12  (0.03-37.19) | 0.17  (0.02-1.38) | 0.47  (0.11-1.99) | 0.40  (0.09-1.69) |  |
| Heterogeneity: τ^2^<0.000; I^2^: 0%. | | | | | | | | | |
| **Heart failure** | | | | | | | | | |
|  | Anakinra | Canakinumab | Colchicine | Cyclosporine | Everolimus | Inclacumab | Methotrexate | Pexelizumab | Tocilizumab |
| Anakinra |  | 3.01  (0.30 – 30.12) | 5.48  (0.55 – 55.02) | 4.79  (0.40 – 56.73) | - | 1.89  (0.04 – 96.88) | 2.99  (0.29 – 31.31) | 2.90  (0.29 – 28.96) | 9.25  (0.18 – 473.69) |
| Canakinumab | 0.33  (0.03 – 3.33) |  | 1.82  (0.48 – 4.12) | 1.59  (0.48 – 5.32) | - | 0.63  (0.02 – 16.92) | 1.00  (0.40 – 2.51) | 0.96  (0.43 – 2.15) | 3.08  (0.11 – 82.71) |
| Colchicine | 0.18  (0.02 – 1.83) | 0.55  (0.24 – 1.24) |  | 0.87  (0.26 – 2.93) | - | 0.35  (0.01 – 9.30) | 0.55  (0.22 – 1.38) | 0.53  (0.24 – 1.19) | 1.69  (0.06 – 45.46) |
| Cyclosporine | 0.21  (0.02 – 2.48) | 0.63  (0.19 – 2.10) | 1.15  (0.26 – 3.85) |  | - | 0.40  (0.17 – 11.99) | 0.63  (0.17 – 2.27) | 0.61  (0.18 – 2.02) | 1.93  (0.06 – 58.60) |
| Everolimus | - | - | - | - |  | - | - | - | - |
| Inclacumab | 0.53  (0.01 – 27.06) | 1.59  (0.06 – 42.71) | 2.90  (0.08 – 77.95) | 2.53  (0.08 – 76.64) | - |  | 1.58  (0.06 – 43.83) | 1.53  (0.06 – 41.07) | 4.89  (0.05 – 478.66) |
| Methotrexate | 0.33  (0.03 – 3.50) | 1.00  (0.40 – 2.53) | 1.83  (0.44 – 4.64) | 1.60  (0.44 – 5.80) | - | 0.63 (0.06 – 17.52) |  | 0.97  (0.39 – 2.43) | 3.10  (0.11 – 85.64) |
| Pexelizumab | 0.35  (0.04 – 3.45) | 1.04  (0.47 – 2.32) | 1.89 (0.50 – 4.25) | 1.65  (0.50 – 5.51) | - | 0.65  (0.41 – 17.54) | 1.03  (0.41 – 2.59) |  | 3.20  (0.12 – 84.53) |
| Tocilizumab | 0.36  (0.00 – 5.53) | 0.33  (0.01 – 8.73) | 0.59  (0.02 – 15.94) | 0.52  (0.02 – 15.68) | - | 0.21  (0.01 – 20.02) | 0.32  (0.01 – 2.21) | 0.31  (0.01 – 8.40) |  |
| Heterogeneity: τ^2^: 0.068; I^2^: 0%. | | | | | | | | | |
| **Revascularization** | | | | | | | | | |
|  | Anakinra | Canakinumab | Colchicine | Cyclosporine | Everolimus | Inclacumab | Methotrexate | Pexelizumab | Tocilizumab |
| Anakinra |  | 1.64  (0.65-4.10) | 1.51  (0.72-3.18) | - | - | 0.81  (0.29-2.23) | 1.28  (0.49-3.38) | 1.09  (0.44-2.66) | - |
| Canakinumab | 0.61  (0.24-1.53) |  | 0.92  (0.43-1.95) | - | - | 0.49  (0.18-1.37) | 0.78  (0.30-2.07) | 0.66  (0.27-1.63) | - |
| Colchicine | 0.66  (0.31-1.40) | 1.09  (0.51-2.30) |  | - | - | 0.54  (0.23-1.27) | 0.85  (0.38-1.91) | 0.72  (0.35-1.48) | - |
| Cyclosporine | - | - | - |  | - | - | - | - | - |
| Everolimus | - | - | - | - |  | - | - | - | - |
| Inclacumab | 1.24  (0.45-3.42) | 2.03  (0.73-5.61) | 1.87  (0.79-4.43) | - | - |  | 1.59  (0.55-4.6) | 1.34  (0.50-3.64) | - |
| Methotrexate | 0.78  (0.30-2.05) | 1.27  (0.48-3.37) | 1.17  (0.52-2.63) | - | - | 0.63  (0.22-1.82) |  | 0.85  (0.33-2.18) | - |
| Pexelizumab | 0.92  (0.38-2.26) | 1.51  (0.61-3.71) | 1.39  (0.68-2.85) | - | - | 0.74  (0.27-2.01) | 1.18  (0.46-3.05) |  | - |
| Tocilizumab | - | - | - | - | - | - | - | - |  |
| Heterogeneity: τ^2^: 0.090; I^2^: 59%. | | | | | | | | | |
| **Stroke** | | | | | | | | | |
|  | Anakinra | Canakinumab | Colchicine | Cyclosporine | Everolimus | Inclacumab | Methotrexate | Pexelizumab | Tocilizumab |
| Anakinra |  | 3.01  (0.26-34.96) | 4.13  (0.39-43.49) | 4.79  (0.35-65.21) | - | 1.89  (0.03-105.9) | 2.99  (0.25-36.24) | 3.00  (0.27-33.07) | 9.25  (0.17-517.77) |
| Canakinumab | 0.33  (0.03-3.87) |  | 1.37  (0.48-3.95) | 1.59  (0.34-7.49) | - | 0.63  (0.02-19.46) | 1.00  (0.26-3.80) | 1.00  (0.31-3.18) | 3.08  (0.10-95.16) |
| Colchicine | 0.24  (0.02-2.55) | 0.73  (0.25-2.09) |  | 1.16  (0.29-4.63) | - | 0.46  (0.02-13.19) | 0.72  (0.23-2.28) | 0.73  (0.29-1.84) | 2.24  (0.08-64.51) |
| Cyclosporine | 0.21  (0.02-2.85) | 0.63  (0.13-2.96) | 0.86  (0.22-3.45) |  | - | 0.40  (0.01-13.72) | 0.63  (0.12-3.14) | 0.63  (0.14-2.71) | 1.93  (0.06-67.1) |
| Everolimus | - | - | - | - |  | - | - | - | - |
| Inclacumab | 0.53  (0.01-29.57) | 1.59  (0.05-49.14) | 2.18  (0.08-62.95) | 2.53  (0.07-87.76) | - |  | 1.58  (0.05-50.37) | 1.58  (0.05-47.2) | 4.89  (0.05-529.93) |
| Methotrexate | 0.33  (0.03-4.04) | 1.00  (0.26-3.84) | 1.38  (0.44-4.35) | 1.60  (0.32-8.02) | - | 0.63  (0.02-20.13) |  | 1.00  (0.29-3.47) | 3.09  (0.10-98.42) |
| Pexelizumab | 0.33  (0.03-3.68) | 1.00  (0.31-3.19) | 1.38  (0.54-3.49) | 1.60  (0.37-6.91) | - | 0.63  (0.02-18.81) | 1.00  (0.29-3.46) |  | 3.09  (0.10-91.96) |
| Tocilizumab | 0.11  (0.00-6.05) | 0.32  (0.01-10.05) | 0.45  (0.02-12.87) | 0.52  (0.01-17.95) | - | 0.20  (0.00-22.17) | 0.32  (0.01-10.3) | 0.32  (0.01-9.65) |  |
| Heterogeneity: τ^2^: 0.068; I^2^: 0%. | | | | | | | | | |
| **Serious infection or sepsis** | | | | | | | | | |
|  | Anakinra | Canakinumab | Colchicine | Cyclosporine | Everolimus | Inclacumab | Methotrexate | Pexelizumab | Tocilizumab |
| Anakinra |  | 0.99  (0.40-2.42) | 1.11  (0.45-2.73) | - | 1.08  (0.21-5.61) | 1.21  (0.43-3.41) | 1.18  (0.47-2.96) | 1.24  (0.48-3.15) | 1.25  (0.31-5.06) |
| Canakinumab | 1.01  (0.41-2.48) |  | 1.12  (0.91-1.38) | - | 1.10  (0.27-4.41) | 1.23  (0.71-2.13) | 1.19  (0.89-1.59) | 1.25  (0.91-1.73) | 1.27  (0.43-3.75) |
| Colchicine | 0.90  (0.37-2.23) | 0.89  (0.72-1.10) |  | - | 0.98  (0.24-3.95) | 1.10  (0.63-1.91) | 1.06  (0.78-1.44) | 1.12  (0.79-1.57) | 1.13  (0.38-3.36) |
| Cyclosporine | - | - | - |  | - | - | - | - | - |
| Everolimus | 0.92  (0.18-4.79) | 0.91  (0.23-3.67) | 1.02  (0.25-4.13) | - |  | 1.12  (0.25-4.95) | 1.09  (0.27-4.46) | 1.14  (0.28-4.71) | 1.16  (0.20-6.70) |
| Inclacumab | 0.82  (0.29-2.32) | 0.81  (0.47-1.41) | 0.91  (0.52-1.60) | - | 0.89  (0.20-3.94) |  | 0.97  (0.54-1.75) | 1.02  (0.55-1.88) | 1.03  (0.31-3.44) |
| Methotrexate | 0.85  (0.34-2.14) | 0.84  (0.63-1.12) | 0.94  (0.69-1.28) | - | 0.92  (0.22-3.76) | 1.03  (0.57-1.86) |  | 1.05  (0.71-1.56) | 1.07  (0.35-3.22) |
| Pexelizumab | 0.81  (0.32-2.06) | 0.80  (0.58-1.10) | 0.90  (0.64-1.26) | - | 0.88  (0.21-3.61) | 0.98  (0.53-1.81) | 0.95  (0.64-1.41) |  | 1.02  (0.33-3.10) |
| Tocilizumab | 0.80  (0.20-3.21) | 0.79  (0.27-2.32) | 0.88  (0.3-2.62) | - | 0.86  (0.15-4.98) | 0.97  (0.29-3.21) | 0.94  (0.31-2.83) | 0.98  (0.32-3.00) |  |
| Heterogeneity: τ^2^<0.001; I^2^: 0%. | | | | | | | | | |
| **Cancer development** | | | | | | | | | |
|  | Anakinra | Canakinumab | Colchicine | Cyclosporine | Everolimus | Inclacumab | Methotrexate | Pexelizumab | Tocilizumab |
| Anakinra |  | - | - | - | - | - | - | - | - |
| Canakinumab | - |  | 0.96  (0.73 – 1.25) | - | - | - | 0.83  (0.60 – 1.14) | - | 0.21  (0.01 – 4.28) |
| Colchicine | - | 1.05  (0.80 – 1.37) |  | - | - | - | 0.87  (0.61 – 1.24) | - | 0.21  (0.01 – 4.49) |
| Cyclosporine | - | - | - |  | - | - | - | - | - |
| Everolimus | - | - | - | - |  | - | - | - | - |
| Inclacumab | - | - | - | - | - |  | - | - | - |
| Methotrexate | - | 1.20  (0.87 – 1.66) | 1.15  (0.81 – 1.63) | - | - | - |  | - | 0.25  (0.01 – 5.20) |
| Pexelizumab | - | - | - | - | - | - | - |  | - |
| Tocilizumab | - | 4.89  (0.23 – 102.17) | 4.67  (0.22 – 98.02) | - | - | - | 4.06  (0.19 – 85.63) | - |  |
| Heterogeneity: τ^2^: 0.000; I^2^: 0%. | | | | | | | | | |
| **Pneumonia** | | | | | | | | | |
|  | Anakinra | Canakinumab | Colchicine | Cyclosporine | Everolimus | Inclacumab | Methotrexate | Pexelizumab | Tocilizumab |
| Anakinra |  | 0.32  (0.01 – 12.24) | 0.23  (0.01 – 7.99) | - | - | - | 1.05  (0.01 – 134.89) | - | - |
| Canakinumab | 3.17  (0.08 – 123.33) |  | 0.72  (0.15 – 3.48) | - | - | - | 3.33  (0.09 – 129.34) | - | - |
| Colchicine | 0.99  (0.39 – 154.55) | 1.39  (0.29 – 6.68) |  | - | - | - | 4.61  (0.13 – 162.09) | - | - |
| Cyclosporine | - | - | - |  | - | - | - | - | - |
| Everolimus | - | - | - | - |  | - | - | - | - |
| Inclacumab | - | - | - | - | - |  | - | - | - |
| Methotrexate | 0.85  (0.32 – 122.63) | 0.30  (0.01 – 11.67) | 0.22  (0.01 – 7.62) | - | - | - |  | - | - |
| Pexelizumab | - | - | - | - | - | - | - |  | - |
| Tocilizumab | - | - | - | - | - | - | - | - |  |
| Heterogeneity: τ^2^: 0.404; I^2^: 69%. | | | | | | | | | |
| **Gastrointestinal adverse events** | | | | | | | | | |
|  | Anakinra | Canakinumab | Colchicine | Cyclosporine | Everolimus | Inclacumab | Methotrexate | Pexelizumab | Tocilizumab |
| Anakinra |  | - | - | - | - | - | - | - | - |
| Canakinumab | - |  | - | - | - | - | - | - | - |
| Colchicine | - | - |  | - | 1.18  (0.17 – 8.48) | - | 0.97  (0.76 – 1.23) | - | - |
| Cyclosporine | - | - | - |  | - | - | - | - | - |
| Everolimus | - | - | 0.84  (0.12 – 6.05) | - |  | - | 0.82  (0.11 – 5.82) | - | - |
| Inclacumab | - | - | - | - | - |  | - | - | - |
| Methotrexate | - | - | 1.04  (0.81 – 1.32) | - | 1.22  (0.17 – 8.77) | - |  | - | - |
| Pexelizumab | - | - | - | - | - | - | - |  | - |
| Tocilizumab | - | - | - | - | - | - | - | - |  |
| Heterogeneity: τ^2^<0.001; I^2^: 46%. | | | | | | | | | |

Values are displayed as incidence rate ratios with respective 95% confidence intervals.
Abbreviations: MACE, major adverse cardiovascular events.

**Supplementary Table 11.** **SUCRA values for prespecified endpoints.**

| **Endpoint** | **Anakinra** | **Canakinumab** | **Colchicine** | **Cyclosporine** | **Everolimus** | **Inclacumab** | **Methotrexate** | **Pexelizumab** | **Placebo** | **Tocilizumab** |
| --- | --- | --- | --- | --- | --- | --- | --- | --- | --- | --- |
| **MACE** | 0.176 | 0.731 | **0.919** | 0.434 | - | - | 0.437 | 0.464 | 0.456 | 0.382 |
| **Serious adverse events** | 0.521 | 0.543 | 0.713 | **0.805** | 0.155 | 0.181 | 0.402 | 0.667 | 0.497 | 0.515 |
| **All-cause death** | 0.539 | **0.709** | 0.545 | 0.428 | 0.546 | 0.144 | 0.400 | 0.588 | 0.611 | 0.490 |
| **Cardiovascular death** | **0.734** | 0.717 | 0.517 | 0.375 | 0.523 | - | 0.365 | - | 0.507 | 0.261 |
| **Myocardial infarction** | 0.008 | 0.655 | 0.675 | 0.791 | 0.694 | 0.141 | 0.440 | 0.287 | 0.432 | **0.800** |
| **Heart failure** | **0.889** | - | 0.443 | 0.511 | - | 0.236 | 0.591 | 0.418 | 0.415 | - |
| **Revascularization** | 0.365 | **0.790** | 0.773 | - | - | 0.190 | 0.590 | 0.426 | 0.367 | - |
| **Stroke** | 0.188 | 0.482 | 0.674 | 0.681 | - | 0.403 | 0.466 | 0.461 | 0.407 | **0.739** |
| **Serious infection or sepsis** | 0.411 | 0.237 | 0.499 | - | 0.464 | 0.610 | 0.598 | **0.661** | 0.437 | 0.584 |
| **Cancer development** | - | **0.797** | 0.642 | - | - | - | 0.351 | - | 0.548 | 0.163 |
| **Pneumonia** | 0.700 | 0.409 | 0.230 | - | - | - | **0.709** | - | 0.453 | - |
| **Gastrointestinal adverse events** | - | - | 0.352 | - | 0.563 | - | 0.268 | - | **0.817** | - |

Values are displayed as SUCRA values after network meta-analysis comparison. Higher values are highlighted by a light-yellow cell.
Abbreviations: MACE, major adverse cardiovascular events; SUCRA, surface under the ranking curve.

**Supplementary Table 12.** **Indirect comparisons on MACE, revascularization and pneumonia after excluding influential trials.**

| **MACE** | | | | | | | | | |
| --- | --- | --- | --- | --- | --- | --- | --- | --- | --- |
|  | Anakinra | Canakinumab | Colchicine | Cyclosporine | Everolimus | Inclacumab | Methotrexate | Pexelizumab | Tocilizumab |
| Anakinra |  | 0.74  (0.22-2.45) | 0.78  (0.24-2.58) | 0.65  (0.19-2.16) | - | - | 0.64  (0.19-2.16) | 0.66  (0.20-2.19) | 0.93  (0.23-3.81) |
| Canakinumab | 1.35  (0.41-4.49) |  | 1.06  (0.88-1.27) | 0.88  (0.69-1.10) | - | - | 0.87  (0.67-1.14) | 0.89  (0.73-1.09) | 1.26  (0.58-2.72) |
| Colchicine | 1.28  (0.39-4.24) | 0.95  (0.79-1.14) |  | 0.83  (0.66-1.04) | - | - | 0.82  (0.64-1.07) | 0.84  (0.69-1.03) | 1.19  (0.55-2.57) |
| Cyclosporine | 1.55  (0.46-5.16) | 1.14  (0.91-1.44) | 1.21  (0.96-1.51) |  | - | - | 1.00  (0.74-1.34) | 1.02  (0.80-1.30) | 1.44  (0.66-3.14) |
| Everolimus | - | - | - | - |  | - | - | - | - |
| Inclacumab | - | - | - | - | - |  | - | - | - |
| Methotrexate | 1.55  (0.46-5.22) | 1.15  (0.88-1.5) | 1.21  (0.93-1.57) | 1.00  (0.76 – 1.32) | - | - |  | 1.02  (0.78-1.35) | 1.45  (0.65-3.19) |
| Pexelizumab | 1.52  (0.46-5.04) | 1.12  (0.91-1.37) | 1.18  (0.97-1.44) | 0.98  (0.79 – 1.22) | - | - | 0.98  (0.74-1.29) |  | 1.41  (0.65-3.06) |
| Tocilizumab | 1.08  (0.26-4.41) | 0.79  (0.37-1.72) | 0.84  (0.39-1.81) | 0.70  (0.32 – 1.51) | - | - | 0.69  (0.31-1.53) | 0.71  (0.33-1.53) |  |
| Heterogeneity: τ^2^:0.002; I^2^: 0%. | | | | | | | | | |
| **Revascularization** | | | | | | | | | |
|  | Anakinra | Canakinumab | Colchicine | Cyclosporine | Everolimus | Inclacumab | Methotrexate | Pexelizumab | Tocilizumab |
| Anakinra |  | 1.64  (0.86 – 3.13) | 2.36  (1.16 – 4.83) | - | - | 0.81  (0.37 – 1.76) | 1.29  (0.63 – 2.63) | 1.06  (0.53 – 2.11) | - |
| Canakinumab | 0.61  (0.32 – 1.17) |  | 1.44  (0.71 – 2.92) | - | - | 0.49  (0.23 – 1.07) | 0.78  (0.39 – 1.59) | 0.65  (0.33 – 1.28) | - |
| Colchicine | 0.42  (0.21 – 0.86) | 0.69  (0.34 – 1.41) |  | - | - | 0.34  (0.15 –0.78) | 0.54  (0.25 – 1.17) | 0.45  (0.21 – 0.95) | - |
| Cyclosporine | - | - | - |  | - | - | - | - | - |
| Everolimus | - | - | - | - |  | - | - | - | - |
| Inclacumab | 1.24  (0.57 – 2.69) | 2.03  (0.94 – 4.38) | 2.92  (1.28 – 6.69) | - | - |  | 1.59  (0.70 – 3.64) | 1.31  (0.58 – 2.94) | - |
| Methotrexate | 0.78  (0.38 – 1.59) | 1.28  (0.63 – 2.59) | 1.84  (0.85 – 3.96) | - | - | 0.63 (0.28 – 1.44) |  | 0.82  (0.39 – 2.57) | - |
| Pexelizumab | 0.95  (0.47 – 1.89) | 1.55  (0.78 – 3.07) | 2.23 (1.06 – 4.72) | - | - | 0.76  (0.34 – 1.71) | 1.22  (0.58 – 2.57) |  | - |
| Tocilizumab | - | - | - | - | - | - | - | - |  |
| Heterogeneity: τ^2^:0.033; I^2^: 0%. | | | | | | | | | |
| **Pneumonia** | | | | | | | | | |
|  | Anakinra | Canakinumab | Colchicine | Cyclosporine | Everolimus | Inclacumab | Methotrexate | Pexelizumab | Tocilizumab |
| Anakinra |  | 0.32  (0.01 – 7.80) | 0.14  (0.01 – 3.65) | - | - | - | 1.05  (0.01 – 96.93) | - | - |
| Canakinumab | 3.17  (0.13 – 78.52) |  | 0.43  (0.20 – 0.95) | - | - | - | 3.33  (0.13 – 82.35) | - | - |
| Colchicine | 7.34  (0.27 – 196.84) | 2.31  (1.05 – 5.09) |  | - | - | - | 7.70  (0.29 – 206.44) | - | - |
| Cyclosporine | - | - | - |  | - | - | - | - | - |
| Everolimus | - | - | - | - |  | - | - | - | - |
| Inclacumab | - | - | - | - | - |  | - | - | - |
| Methotrexate | 0.95  (0.01 – 88.12) | 0.30  (0.01 – 7.43) | 0.13  (0.01 – 3.48) | - | - | - |  | - | - |
| Pexelizumab | - | - | - | - | - | - | - |  | - |
| Tocilizumab | - | - | - | - | - | - | - | - |  |
| Heterogeneity: τ^2^<0.001; I^2^: 0%. | | | | | | | | | |
| **Gastrointestinal adverse events** | | | | | | | | | |
|  | Anakinra | Canakinumab | Colchicine | Cyclosporine | Everolimus | Inclacumab | Methotrexate | Pexelizumab | Tocilizumab |
| Anakinra |  | - | - | - | - | - | - | - | - |
| Canakinumab | - |  | - | - | - | - | - | - | - |
| Colchicine | - | - |  | - | 1.57  (0.16 – 8.29) | - | 0.94  (0.74 – 1.20) | - | - |
| Cyclosporine | - | - | - |  | - | - | - | - | - |
| Everolimus | - | - | 0.86  (0.12 – 6.19) | - |  | - | 0.82  (0.11 – 5.82) | - | - |
| Inclacumab | - | - | - | - | - |  | - | - | - |
| Methotrexate | - | - | 1.06  (0.83 – 1.35) | - | 1.23  (0.17 – 8.77) | - |  | - | - |
| Pexelizumab | - | - | - | - | - | - | - |  | - |
| Tocilizumab | - | - | - | - | - | - | - | - |  |
| Heterogeneity: τ^2^<0.001; I^2^: 1%. | | | | | | | | | |

**Supplementary Table 13.** **Sensitivity analyses for MACE.**

| **Drug administered** | **Comparisons** | **IRR (95% CI)** | **Heterogeneity** |
| --- | --- | --- | --- |
| **Consistent definition** | | | |
| Anakinra | 1 | 3.11 (1.01 – 9.54) | - |
| Canakinumab | 1 | 0.89 (0.80 – 0.99) | - |
| Colchicine | 3 | 0.86 (0.72 – 1.02) | τ^2^:0.009; I^2^: 40% |
| Cyclosporine | 0 | - | - |
| Everolimus | 0 | - | - |
| Inclacumab | 0 | - | - |
| Methotrexate | 0 | - | - |
| Pexelizumab | 0 | - | - |
| Tocilizumab | 0 | - | - |
| **Overall** | **5** | **0.89 (0.82 – 0.99)** | **τ^2^<0.001; I^2^: 51%** |
| **P for interaction: 0.084** |  |  |  |
| **Only acute phase** | | | |
| Anakinra | 1 | 0.66 (0.20 – 4.02) | - |
| Canakinumab | 0 | - | - |
| Colchicine | 6 | 0.84 (0.70 – 1.02) | τ^2^:0.014; I^2^: 45% |
| Cyclosporine | 2 | 1.02 (0.86 – 1.21) | τ^2^:0.000; I^2^: 0% |
| Everolimus | 0 | - | - |
| Inclacumab | 0 | - | - |
| Methotrexate | 0 | - | - |
| Pexelizumab | 3 | 1.00 (0.87 – 1.15) | τ^2^:0.000; I^2^: 0% |
| Tocilizumab | 3 | 1.19 (0.35 – 4.02) | τ^2^:0.782; I^2^: 66% |
| **Overall** | **15** | **0.96 (0.88 – 1.04)** | **τ^2^<0.001; I^2^: 23%** |
| **P for interaction: 0.538** |  |  |  |
| **Only STEMI** | | | |
| Anakinra | 1 | 0.66 (0.20 – 2.15) | - |
| Canakinumab | 0 | - | - |
| Colchicine | 3 | 0.94 (0.80 – 1.10) | τ^2^:0.000; I^2^: 0% |
| Cyclosporine | 2 | 1.02 (0.86 – 1.21) | τ^2^:0.000; I^2^: 0% |
| Everolimus | 0 | - | - |
| Inclacumab | 0 | - | - |
| Methotrexate | 0 | - | - |
| Pexelizumab | 3 | 1.00 (0.87 – 1.15) | τ^2^:0.000; I^2^: 0% |
| Tocilizumab | 1 | 0.82 (0.33 – 2.01) | τ^2^:0.000; I^2^: 0% |
| **Overall** | **10** | **0.98 (0.90 – 1.07)** | **τ^2^:0.000; I^2^: 0%** |
| **P for interaction: 0.889** |  |  |  |
| **Only phase 3 trials** | | | |
| Anakinra | 0 | - | - |
| Canakinumab | 1 | 0.89 (0.80 – 0.99) | - |
| Colchicine | 6 | 0.77 (0.63 – 0.95) | τ^2^: 0.031; I^2^: 58% |
| Cyclosporine | 1 | 1.02 (0.85 – 1.21) | - |
| Everolimus | 0 | - | - |
| Inclacumab | 0 | - | - |
| Methotrexate | 1 | 1.02 (0.82 – 1.26) | - |
| Pexelizumab | 1 | 1.01 (0.86 – 1.19) | - |
| Tocilizumab | 0 | - | - |
| **Overall** | **10** | **0.91 (0.85 – 0.98)** | **τ^2^: 0.003; I^2^: 51%** |
| **P for interaction: 0.707** |  |  |  |
| **Long-term follow-up** | | | |
| Anakinra | 2 | 1.45 (0.86 – 1.21) | τ^2^: 0.012; I^2^: 43% |
| Canakinumab | 1 | 0.89 (0.80 – 0.99) | - |
| Colchicine | 7 | 0.79 (0.66 – 0.93) | τ^2^:0.019; I^2^: 49% |
| Cyclosporine | 2 | 1.02 (0.86 – 1.21) | τ^2^: 0.000; I^2^: 0% |
| Everolimus | 0 | - | - |
| Inclacumab | 0 | - | - |
| Methotrexate | 1 | 1.02 (0.82 – 1.26) | - |
| Pexelizumab | 0 | - | - |
| Tocilizumab | 2 | 0.71 (0.33 – 1.50) | τ^2^: 0.000; I^2^: 0% |
| **Overall** | **14** | **0.90 (0.83 – 0.97)** | **τ^2^: 0.001; I^2^: 38%** |
| **P for interaction: 0.268** |  |  |  |
| **Patients receiving PCI** |  |  |  |
| Anakinra | 0 | - | - |
| Canakinumab | 0 | - | - |
| Colchicine | 7 | 0.77 (0.62 – 0.97) | τ^2^: 0.036; I^2^: 47% |
| Cyclosporine | 2 | 1.02 (0.86 – 1.21) | τ^2^: 0.000; I^2^: 0% |
| Everolimus | 0 | - | - |
| Inclacumab | 0 | - | - |
| Methotrexate | 0 | - | - |
| Pexelizumab | 1 | 1.01 (0.86 – 1.19) | - |
| Tocilizumab | 1 | 0.82 (0.33 – 2.01) | - |
| **Overall** | **11** | **0.94 (0.86 – 1.02)** | **τ^2^ <0.001; I^2^: 32%** |
| **P for interaction: 0.228** |  |  |  |

Light blue cells highlight significant reduction with treatment, while light red cells highlight significant increase with treatment. Significant p-values for interaction are shown in red.
Abbreviations: CI, confidence interval; IRR, incidence rate ratio; MACE, major adverse cardiovascular events.

**Supplementary Table 14.** **Sensitivity analyses for serious adverse events.**

| **Drug administered** | **Comparisons** | **IRR (95% CI)** | **Heterogeneity** |
| --- | --- | --- | --- |
| **Only acute phase** | | | |
| Anakinra | 2 | 0.99 (0.38 – 2.57) | τ^2^:0.000; I^2^: 0% |
| Canakinumab | 0 | - | - |
| Colchicine | 3 | 0.93 (0.84 – 1.04) | τ^2^:0.000; I^2^: 0% |
| Cyclosporine | 2 | 0.63 (0.12 – 3.21) | τ^2^:0.419; I^2^: 21% |
| Everolimus | 1 | 1.58 (0.77 – 3.26) | - |
| Inclacumab | 1 | 1.36 (0.91 – 2.03) | - |
| Methotrexate | 0 | - | - |
| Pexelizumab | 1 | 0.90 (0.64 – 1.26) | - |
| Tocilizumab | 3 | 1.00 (0.55 – 1.81) | τ^2^:0.000; I^2^: 0% |
| **Overall** | **13** | **0.98 (0.87 – 1.06)** | **τ^2^: 0.000; I^2^: 0%** |
| **P for interaction: 0.501** |  |  |  |
| **Only STEMI** | | | |
| Anakinra | 1 | 0.98 (0.38 – 2.87) | - |
| Canakinumab | 0 | - | - |
| Colchicine | 1 | 0.90 (0.76 – 1.08) | τ^2^:0.000; I^2^: 0% |
| Cyclosporine | 2 | 0.63 (0.12 – 3.21) | τ^2^:0.419; I^2^: 21% |
| Everolimus | 1 | 1.58 (0.77 – 3.26) | - |
| Inclaclumab | 1 | 1.36 (0.91 – 2.03) | - |
| Methotrexate | 0 | - | - |
| Pexelizumab | 1 | 0.90 (0.64 – 1.26) | - |
| Tocilizumab | 1 | 1.49 (0.58 – 2.26) | - |
| **Overall** | **7** | **1.00 (0.84 – 1.20)** | **τ^2^:0.011; I^2^: 0%** |
| **P for interaction: 0.450** |  |  |  |
| **Only phase 3 trials** | | | |
| Anakinra | 0 | - | - |
| Canakinumab | 1 | 0.99 (0.92 – 1.06) | - |
| Colchicine | 3 | 0.93 (0.83 – 1.03) | τ^2^: 0.000; I^2^: 0% |
| Cyclosporine | 0 | - | - |
| Everolimus | 0 | - | - |
| Inclaclumab | 0 | - | - |
| Methotrexate | 1 | 1.04 (0.92 – 1.17) | - |
| Pexelizumab | 1 | 0.90 (0.64 – 1.26) | - |
| Tocilizumab | 0 | - | - |
| **Overall** | **6** | **0.98 (0.93 – 1.03)** | **τ^2^: 0.000; I^2^: 0%** |
| **P for interaction: 0.541** |  |  |  |
| **Long-term follow-up** | | | |
| Anakinra | 1 | 0.98 (0.33 – 2.94) | - |
| Canakinumab | 1 | 0.99 (0.92 – 1.06) | - |
| Colchicine | 3 | 0.95 (0.84 – 1.03) | τ^2^:0.000; I^2^: 0% |
| Cyclosporine | 1 | 2.94 (0.12 – 72.22) | - |
| Everolimus | 0 | - | - |
| Inclacumab | 0 | - | - |
| Methotrexate | 1 | 1.04 (0.92 – 1.17) | - |
| Pexelizumab | 0 | - | - |
| Tocilizumab | 2 | 1.02 (0.55 – 1.88) | τ^2^: 0.000; I^2^: 0% |
| **Overall** | **9** | **0.98 (0.94 – 1.04)** | **τ^2^: 0.000; I^2^: 0%** |
| **P for interaction: 0.801** |  |  |  |
| **Patients receiving PCI** | | | |
| Anakinra | 1 | 1.00 (0.14 – 7.10) | - |
| Canakinumab | 0 | - | - |
| Colchicine | 4 | 0.93 (0.84 – 1.03) | τ^2^: 0.000; I^2^: 0% |
| Cyclosporine | 2 | 0.63 (0.12 – 13.21) | τ^2^: 0.419; I^2^: 21% |
| Everolimus | 1 | 1.58 (0.77 – 3.26) | - |
| Inclacumab | 1 | 1.36 (0.91 – 2.03) | - |
| Methotrexate | 0 | - | - |
| Pexelizumab | 1 | 0.90 (0.64 – 1.26) | - |
| Tocilizumab | 1 | 1.15 (0.58 – 2.26) | - |
| **Overall** | **11** | **0.96 (0.87 – 1.05)** | **τ^2^: 0.000; I^2^: 0%** |
| **P for interaction: 0.463** |  |  |  |

Light blue cells highlight significant reduction with treatment, while light red cells highlight significant increase with treatment. Significant p-values for interaction are shown in red.
Abbreviations: CI, confidence interval; IRR, incidence rate ratio.

**Supplementary Table 15. Sensitivity analyses for all-cause death.**

| **Drug administered** | **Comparisons** | **IRR (95% CI)** | **Heterogeneity** |
| --- | --- | --- | --- |
| **Only acute phase** | | | |
| Anakinra | 2 | 0.25 (0.03 – 2.37) | τ^2^:0.000; I^2^: 0% |
| Canakinumab | 0 | - | - |
| Colchicine | 6 | 0.94 (0.78 – 1.13) | τ^2^:0.000; I^2^: 0% |
| Cyclosporine | 4 | 1.34 (0.74 – 2.44) | τ^2^:0.138; I^2^: 26% |
| Everolimus | 1 | 1.00 (0.02 – 50.40) | - |
| Inclacumab | 1 | 6.41 (0.36 – 113.76) | - |
| Methotrexate | 1 | 1.95 (0.36 – 10.65) | - |
| Pexelizumab | 3 | 0.94 (0.64 – 1.38) | τ^2^:0.066; I^2^: 56% |
| Tocilizumab | 1 | 1.46 (0.03 – 73.31) | - |
| **Overall** | **19** | **1.00 (0.88 – 1.53)** | **τ^2^: 0.000; I^2^: 0%** |
| **P for interaction: 0.704** |  |  |  |
| **Only STEMI** | | | |
| Anakinra | 2 | 0.25 (0.03 – 2.37) | τ^2^:0.000; I^2^: 0% |
| Canakinumab | 0 | - | - |
| Colchicine | 3 | 0.91 (0.74 – 1.13) | τ^2^:0.000; I^2^: 0% |
| Cyclosporine | 4 | 1.34 (0.73 – 2.44) | τ^2^:0.138; I^2^: 26% |
| Everolimus | 1 | 1.00 (0.02 – 50.40) | - |
| Inclaclumab | 1 | 6.41 (0.36 – 113.76) | - |
| Methotrexate | 1 | 1.95 (0.31 – 10.65) | - |
| Pexelizumab | 3 | 0.93 (0.64 – 1.38) | τ^2^:0.66; I^2^: 56% |
| Tocilizumab | 0 | - | - |
| **Overall** | **15** | **0.99 (0.87 – 1.38)** | **τ^2^<0.001; I^2^: 0%** |
| **P for interaction: 0.574** |  |  |  |
| **Only phase 3 trials** | | | |
| Anakinra | 0 | - | - |
| Canakinumab | 1 | 0.94 (0.83 – 1.06) | - |
| Colchicine | 5 | 1.03 (0.84 – 1.27) | τ^2^: 0.010; I^2^: 41% |
| Cyclosporine | 1 | 1.08 (0.63 – 1.84) | - |
| Everolimus | 0 | - | - |
| Inclaclumab | 0 | - | - |
| Methotrexate | 1 | 1.16 (0.86 – 1.55) | - |
| Pexelizumab | 1 | 1.09 (0.86 – 1.39) | - |
| Tocilizumab | 0 | - | - |
| **Overall** | **9** | **1.01 (0.91 – 1.11)** | τ^2^: 0.002; I^2^: 15% |
| **P for interaction: 0.600** |  |  |  |
| **Long-term follow-up** | | | |
| Anakinra | 2 | 0.97 (0.09 – 2.55) | τ^2^:1.631; I^2^: 50% |
| Canakinumab | 1 | 0.94 (0.83 – 1.06) | - |
| Colchicine | 6 | 1.03 (0.85 – 1.26) | τ^2^: 0.009; I^2^: 26% |
| Cyclosporine | 3 | 1.36 (0.72 – 2.55) | τ^2^:0.157; I^2^: 50% |
| Everolimus | 0 | - | - |
| Inclacumab | 0 | - | - |
| Methotrexate | 1 | 1.16 (0.86 – 1.55) | - |
| Pexelizumab | 0 | - | - |
| Tocilizumab | 0 | - | - |
| **Overall** | **13** | **1.01 (0.90 – 1.12)** | **τ^2^: 0.003; I^2^: 7%** |
| **P for interaction: 0.625** |  |  |  |
| **Patients receiving PCI** | | | |
| Anakinra | 1 | 0.33 (0.01 – 8.18) | - |
| Canakinumab | 0 | - | - |
| Colchicine | 7 | 0.95 (0.79 – 1.15) | τ^2^: 0.000; I^2^: 0% |
| Cyclosporine | 3 | 1.59 (0.66 – 3.84) | τ^2^: 0.286; I^2^: 42% |
| Everolimus | 1 | 1.00 (0.02 – 50.40) | - |
| Inclacumab | 1 | 6.41 (0.36 – 113.76) | - |
| Methotrexate | 1 | 1.95 (0.36 – 10.65) | - |
| Pexelizumab | 1 | 1.09 (0.86 – 1.39) | - |
| Tocilizumab | **0** | - | - |
| **Overall** | **15** | **1.03 (0.89 – 1.19)** | **τ^2^: 0.001; I^2^: 0%** |
| **P for interaction: 0.664** |  |  |  |

Light blue cells highlight significant reduction with treatment, while light red cells highlight significant increase with treatment. Significant p-values for interaction are shown in red.
Abbreviations: CI, confidence interval; IRR, incidence rate ratio.

**Supplementary Table 16. Sensitivity analyses for cardiovascular death.**

| **Drug administered** | **Comparisons** | **IRR (95% CI)** | **Heterogeneity** |
| --- | --- | --- | --- |
| **Only acute phase** | | | |
| Anakinra | 1 | 0.33 (0.01 – 8.18) | - |
| Canakinumab | 0 | - | - |
| Colchicine | 4 | 1.02 (0.81 – 1.29) | τ^2^:0.000; I^2^: 0% |
| Cyclosporine | 3 | 1.21 (0.65 – 2.25) | τ^2^:0.053; I^2^: 0% |
| Everolimus | 1 | 1.00 (0.02 – 50.40) | - |
| Inclacumab | 0 | - | - |
| Methotrexate | 0 | - | - |
| Pexelizumab | 0 | - | - |
| Tocilizumab | 2 | 2.25 (0.19 – 26.80) | τ^2^: 0.000; I^2^: 0% |
| **Overall** | **11** | **1.04 (0.84 – 1.29)** | **τ^2^: 0.000; I^2^: 0%** |
| **P for interaction: 0.891** |  |  |  |
| **Only STEMI** | | | |
| Anakinra | 1 | 0.33 (0.01 – 8.18) | - |
| Canakinumab | 0 | - | - |
| Colchicine | 1 | 1.04 (0.80 – 1.34) | τ^2^:0.000; I^2^: 0% |
| Cyclosporine | 3 | 1.21 (0.65 – 2.25) | τ^2^:0.052; I^2^: 0% |
| Everolimus | 1 | 1.00 (0.02 – 50.40) | - |
| Inclaclumab | 0 | - | - |
| Methotrexate | 0 | - | - |
| Pexelizumab | 0 | - | - |
| Tocilizumab | 0 | - | - |
| **Overall** | **6** | **1.05 (0.84 – 1.33)** | **τ^2^:0.000; I^2^: 0%** |
| **P for interaction: 0.872** |  |  |  |
| **Only phase 3 trials** | | | |
| Anakinra | 0 | - | - |
| Canakinumab | 1 | 0.87 (0.73 – 1.05) | - |
| Colchicine | 5 | 0.99 (0.80 – 1.24) | τ^2^: 0.000; I^2^: 0% |
| Cyclosporine | 1 | 1.00 (0.57 – 1.77) | - |
| Everolimus | 0 | - | - |
| Inclaclumab | 0 | - | - |
| Methotrexate | 1 | 1.14 (0.76 – 1.72) | - |
| Pexelizumab | 0 | - | - |
| Tocilizumab | 0 | - | - |
| **Overall** | **8** | **0.95 (0.83 – 1.07)** | **τ^2^: 0.000; I^2^: 0%** |
| **P for interaction: 0.614** |  |  |  |
| **Long-term follow-up** | | | |
| Anakinra | 0 | - | - |
| Canakinumab | 1 | 0.87 (0.73 – 1.05) | - |
| Colchicine | 5 | 0.99 (0.80 – 1.24) | τ^2^:0.000; I^2^: 0% |
| Cyclosporine | 2 | 1.25 (0.62 – 2.49) | τ^2^:0.089; I^2^: 29% |
| Everolimus | 0 | - | - |
| Inclacumab | 0 | - | - |
| Methotrexate | 1 | 1.14 (0.76 – 1.72) | - |
| Pexelizumab | 0 | - | - |
| Tocilizumab | 1 | 3.00 (0.12 – 73.64) | - |
| **Overall** | **10** | **0.96 (0.84 – 1.09)** | **τ^2^: 0.000; I^2^: 0%** |
| **P for interaction: 0.580** |  |  |  |
| **Patients receiving PCI** | | | |
| Anakinra | 1 | 0.33 (0.01 – 8.18) | - |
| Canakinumab | 0 | - | - |
| Colchicine | 5 | 1.03 (0.82 – 1.30) | τ^2^: 0.000; I^2^: 0% |
| Cyclosporine | 3 | 1.21 (0.66 – 2.25) | τ^2^: 0.053; I^2^: 0% |
| Everolimus | 1 | 1.00 (0.02 – 50.40) | - |
| Inclacumab | 0 | - | - |
| Methotrexate | 0 | - | - |
| Pexelizumab | 0 | - | - |
| Tocilizumab | 0 | - | - |
| **Overall** | **10** | **1.05 (0.85 – 1.29)** | **τ^2^: 0.000; I^2^: 0%** |
| **P for interaction: 0.868** |  |  |  |

Light blue cells highlight significant reduction with treatment, while light red cells highlight significant increase with treatment. Significant p-values for interaction are shown in red.
Abbreviations: CI, confidence interval; IRR, incidence rate ratio.

**Supplementary Table 17. Sensitivity analyses for myocardial infarction.**

| **Drug administered** | **Comparisons** | **IRR (95% CI)** | **Heterogeneity** |
| --- | --- | --- | --- |
| **Only acute phase** | | | |
| Anakinra | 1 | 3.00 (0.12 – 73.64) | - |
| Canakinumab | 0 | - | - |
| Colchicine | 4 | 0.91 (0.75 – 1.10) | τ^2^:0.000; I^2^: 0% |
| Cyclosporine | 1 | 0.60 (0.26 – 1.38) | - |
| Everolimus | 1 | 0.42 (0.02 – 10.37) | - |
| Inclacumab | 1 | 2.71 (0.60 – 12.23) | - |
| Methotrexate | 1 | 4.88 (0.23 – 101.61) | - |
| Pexelizumab | 3 | 1.19 (0.89 – 1.59) | τ^2^:0.000; I^2^: 0% |
| Tocilizumab | 3 | 0.46 (0.67 – 3.11) | τ^2^: 1.275; I^2^: 44% |
| **Overall** | **15** | **0.97 (0.82 – 1.16)** | **τ^2^: 0.006; I^2^: 7.2%** |
| **P for interaction: 0.346** |  |  |  |
| **Only STEMI** | | | |
| Anakinra | 1 | 3.00 (0.12 – 73.64) | - |
| Canakinumab | 0 | - | - |
| Colchicine | 2 | 0.91 (0.70 – 1.19) | τ^2^: 0.020; I^2^: 9% |
| Cyclosporine | 1 | 0.60 (0.26 – 1.38) | - |
| Everolimus | 1 | 0.42 (0.02 – 10.37) | - |
| Inclaclumab | 1 | 2.71 (0.60 – 12.23) | - |
| Methotrexate | 1 | 4.88 (0.23 – 101.61) | - |
| Pexelizumab | 3 | 1.19 (0.89 – 1.59) | τ^2^:0.000; I^2^: 0% |
| Tocilizumab | 1 | - | - |
| **Overall** | **11** | **1.00 (0.79 – 1.29)** | **τ^2^:0.020; I^2^: 9%** |
| **P for interaction: 0.247** |  |  |  |
| **Only phase 3 trials** | | | |
| Anakinra | 0 | - | - |
| Canakinumab | 1 | 0.86 (0.74 – 0.99) | - |
| Colchicine | 5 | 0.84 (0.72 – 0.99) | τ^2^<0.001; I^2^: 0% |
| Cyclosporine | 1 | 0.60 (0.26 – 1.38) | - |
| Everolimus | 0 | - | - |
| Inclaclumab | 0 | - | - |
| Methotrexate | 1 | 0.99 (0.77 – 1.29) | - |
| Pexelizumab | 1 | 1.27 (0.93 – 1.75) | - |
| Tocilizumab | 0 | - | - |
| **Overall** | **9** | **0.90 (0.80 – 1.02)** | **τ^2^: 0.007; I^2^: 24%** |
| **P for interaction: 0.128** |  |  |  |
| **Long-term follow-up** | | | |
| Anakinra | 1 | 3.83 (0.81 – 18.03) | - |
| Canakinumab | 1 | 0.86 (0.74 – 0.99) | - |
| Colchicine | 5 | 0.84 (0.72 – 0.99) | τ^2^<0.001; I^2^: 0% |
| Cyclosporine | 1 | 0.60 (0.26 – 1.38) | - |
| Everolimus | 0 | - | - |
| Inclacumab | 0 | - | - |
| Methotrexate | 1 | 0.99 (0.77 – 1.29) | - |
| Pexelizumab | 0 | - | - |
| Tocilizumab | 2 | 0.18 (0.03 – 1.04) | τ^2^:0.000; I^2^: 0% |
| **Overall** | **11** | **0.87 (0.79 – 0.96)** | **τ^2^ <0.001; I^2^: 18%** |
| **P for interaction: 0.128** |  |  |  |
| **Patients receiving PCI** | | | |
| Anakinra | 1 | 3.00 (0.12 – 73.64) | - |
| Canakinumab | 0 | - | - |
| Colchicine | 5 | 0.91 (0.75 – 1.10) | τ^2^: 0.000; I^2^: 0% |
| Cyclosporine | 1 | 0.60 (0.26 – 1.38) | - |
| Everolimus | 1 | 0.42 (0.02 – 10.37) | - |
| Inclacumab | 1 | 2.71 (0.60 – 12.23) | - |
| Methotrexate | 1 | 4.88 (0.23 – 101.61) | - |
| Pexelizumab | 1 | 1.27 (0.93 – 1.75) | - |
| Tocilizumab | 1 | 0.10 (0.01 – 1.87) | - |
| **Overall** | **12** | **0.99 (0.82 – 1.19)** | **τ^2^: 0.009; I^2^: 10%** |
| **P for interaction: 0.185** |  |  |  |

Light blue cells highlight significant reduction with treatment, while light red cells highlight significant increase with treatment. Significant p-values for interaction are shown in red.
Abbreviations: CI, confidence interval; IRR, incidence rate ratio.

**Supplementary Table 18. Sensitivity analyses for heart failure.**

| **Drug administered** | **Comparisons** | **IRR (95% CI)** | **Heterogeneity** |
| --- | --- | --- | --- |
| **Only acute phase** | | | |
| Anakinra | 1 | 0.25 (0.03 – 2.24) | - |
| Canakinumab | 0 | - | - |
| Colchicine | 4 | 0.92 (0.44 – 1.92) | τ^2^:0.198; I^2^: 22% |
| Cyclosporine | 4 | 0.96 (0.75 – 1.22) | τ^2^<0.001; I^2^: 0% |
| Everolimus | 0 | - | - |
| Inclacumab | 1 | 2.46 (0.12 – 51.34) | - |
| Methotrexate | 0 | - | - |
| Pexelizumab | 3 | 1.01 (0.83 – 1.24) | τ^2^:0.000; I^2^: 0% |
| Tocilizumab | 0 | - | - |
| **Overall** | **13** | **0.98 (0.85 – 1.14)** | **τ^2^: 0.000; I^2^: 0%** |
| **P for interaction: 0.735** |  |  |  |
| **Only STEMI** | | | |
| Anakinra | 1 | 0.25 (0.03 – 2.24) | - |
| Canakinumab | 0 | - | - |
| Colchicine | 2 | 0.62 (0.31 – 1.26) | τ^2^:0.000; I^2^: 0% |
| Cyclosporine | 4 | 0.96 (0.75 – 1.22) | τ^2^<0.001; I^2^: 0% |
| Everolimus | 0 | - | - |
| Inclaclumab | 1 | 2.47 (0.12 – 51.34) | - |
| Methotrexate | 0 | - | - |
| Pexelizumab | 3 | 1.01 (0.83 – 1.24) | τ^2^:0.000; I^2^: 0% |
| Tocilizumab | 0 | - | - |
| **Overall** | **10** | **0.96 (0.83 – 1.12)** | **τ^2^:0.000; I^2^: 0%** |
| **P for interaction: 0.470** |  |  |  |
| **Only phase 3 trials** | | | |
| Anakinra | 0 | - | - |
| Canakinumab | 0 | - | - |
| Colchicine | 2 | 1.41 (0.77 – 2.58) | τ^2^:0.000; I^2^: 0% |
| Cyclosporine | 1 | 1.00 (0.75 – 1.34) | - |
| Everolimus | 0 | - | - |
| Inclaclumab | 0 | - | - |
| Methotrexate | 1 | 0.90 (0.61 – 1.34) | - |
| Pexelizumab | 1 | 0.99 (0.78 – 1.25) | - |
| Tocilizumab | 0 | - | - |
| **Overall** | **5** | **1.00 (0.85 – 1.18)** | **τ^2^: 0.000; I^2^: 0%** |
| **P for interaction: 0.590** |  |  |  |
| **Long-term follow-up** | | | |
| Anakinra | 0 | - | - |
| Canakinumab | 0 | - | - |
| Colchicine | 3 | 0.91 (0.40 – 2.06) | τ^2^:0.249; I^2^: 48% |
| Cyclosporine | 3 | 0.98 (0.77 – 1.25) | τ^2^:0.000; I^2^: 0% |
| Everolimus | 0 | - | - |
| Inclacumab | 0 | - | - |
| Methotrexate | 1 | 0.91 (0.61 – 1.34) | - |
| Pexelizumab | 0 | - | - |
| Tocilizumab | 0 | - | - |
| **Overall** | **7** | **0.96 (0.80 – 1.16)** | **τ^2^:0.000; I^2^: 0%** |
| **P for interaction: 0.943** |  |  |  |
| **Patients receiving PCI** | | | |
| Anakinra | 1 | 0.25 (0.03 – 2.24) | - |
| Canakinumab | 0 | - | - |
| Colchicine | 4 | 0.92 (0.44 – 1.92) | τ^2^: 0.198; I^2^: 22% |
| Cyclosporine | 3 | 0.95 (0.74 – 1.24) | τ^2^<0.001; I^2^: 33% |
| Everolimus | 0 | - | - |
| Inclacumab | 1 | 2.47 (0.12 – 51.34) | - |
| Methotrexate | 0 | - | - |
| Pexelizumab | 1 | 0.99 (0.78 – 1.25) | - |
| Tocilizumab | 0 | - | - |
| **Overall** | **10** | **0.97 (0.82 – 1.14)** | **τ^2^: 0.000; I^2^: 0%** |
| **P for interaction: 0.185** |  |  |  |

Light blue cells highlight significant reduction with treatment, while light red cells highlight significant increase with treatment. Significant p-values for interaction are shown in red.
Abbreviations: CI, confidence interval; IRR, incidence rate ratio.

**Supplementary Table 19. Sensitivity analyses for revascularization.**

| **Drug administered** | **Comparisons** | **IRR (95% CI)** | **Heterogeneity** |
| --- | --- | --- | --- |
| **Only acute phase** | | | |
| Anakinra | 1 | 1.00 (0.06 – 15.99) | - |
| Canakinumab | 0 | - | - |
| Colchicine | 2 | 0.73 (0.38 – 1.41) | τ^2^:0.193; I^2^: 84% |
| Cyclosporine | 0 | - | - |
| Everolimus | 0 | - | - |
| Inclacumab | 1 | 1.31 (0.78 – 2.18) | - |
| Methotrexate | 0 | - | - |
| Pexelizumab | 2 | 0.98 (0.55 – 1.75) | τ^2^:0.065; I^2^: 35% |
| Tocilizumab | 0 | - | - |
| **Overall** | **6** | **0.91 (0.64 – 1.27)** | **τ^2^: 0.091; I^2^: 50%** |
| **P for interaction: 0.596** |  |  |  |
| **Only STEMI** | | | |
| Anakinra | 1 | 1.00 (0.06 – 1.75) | - |
| Canakinumab | 0 | - | - |
| Colchicine | 1 | 0.99 (0.80 – 1.23) | - |
| Cyclosporine | 0 | - | - |
| Everolimus | 0 | - | - |
| Inclaclumab | 1 | 1.31 (0.78 – 2.19) | - |
| Methotrexate | 0 | - | - |
| Pexelizumab | 2 | 0.98 (0.55 – 1.75) | τ^2^:0.065; I^2^: 35% |
| Tocilizumab | 0 | - | - |
| **Overall** | **5** | **1.03 (0.86 – 1.24)** | **τ^2^:0.000; I^2^: 0%** |
| **P for interaction: 0.807** |  |  |  |
| **Only phase 3 trials** | | | |
| Anakinra | 0 | - | - |
| Canakinumab | 1 | 0.64 (0.49 – 0.86) | - |
| Colchicine | 4 | 0.69 (0.47 – 1.02) | τ^2^:0.104; I^2^: 72% |
| Cyclosporine | 0 | - | - |
| Everolimus | 0 | - | - |
| Inclaclumab | 0 | - | - |
| Methotrexate | 1 | 0.82 (0.54 – 1.24) | - |
| Pexelizumab | 0 | - | - |
| Tocilizumab | 0 | - | - |
| **Overall** | **6** | **0.73 (0.59 – 0.91)** | **τ^2^ :0.037; I^2^: 61%** |
| **P for interaction: 0.636** |  |  |  |
| **Long-term follow-up** | | | |
| Anakinra | 1 | 1.06 (0.78 – 1.44) | - |
| Canakinumab | 1 | - | - |
| Colchicine | 4 | 0.69 (0.47 – 1.02) | τ^2^:0.104; I^2^: 72% |
| Cyclosporine | 0 | - | - |
| Everolimus | 0 | - | - |
| Inclacumab | 0 | - | - |
| Methotrexate | 1 | 0.82 (0.54 – 1.24) | - |
| Pexelizumab | 0 | - | - |
| Tocilizumab | 0 | - | - |
| **Overall** | **7** | **0.77 (0.63 – 0.95)** | **τ^2^:0.041; I^2^: 63%** |
| **P for interaction: 0.119** |  |  |  |
| **Patients receiving PCI** | | | |
| Anakinra | 1 | 1.00 (0.06 – 15.99) | - |
| Canakinumab | 0 | - | - |
| Colchicine | 2 | 0.60 (0.30 – 1.19) | τ^2^:0.269; I^2^: 80% |
| Cyclosporine | 0 | - | - |
| Everolimus | 0 | - | - |
| Inclacumab | 1 | 1.31 (0.78 – 2.19) | - |
| Methotrexate | 0 | - | - |
| Pexelizumab | 0 | - | - |
| Tocilizumab | 0 | - | - |
| **Overall** | **5** | **0.60 (0.30 – 1.19)** | **τ^2^: 0.225; I^2^: 68%** |
| **P for interaction: 0.206** |  |  |  |

Light blue cells highlight significant reduction with treatment, while light red cells highlight significant increase with treatment. Significant p-values for interaction are shown in red.
Abbreviations: CI, confidence interval; IRR, incidence rate ratio.

**Supplementary Table 20. Sensitivity analyses for stroke.**

| **Drug administered** | **Comparisons** | **IRR (95% CI)** | **Heterogeneity** |
| --- | --- | --- | --- |
| **Only acute phase** | | | |
| Anakinra | 1 | 2.73 (0.13 – 56.96) | - |
| Canakinumab | 0 | - | - |
| Colchicine | 3 | 0.72 (0.25 – 2.06) | τ^2^:0.584; I^2^: 74% |
| Cyclosporine | 1 | 0.59 (0.23 – 1.49) | - |
| Everolimus | 0 | - | - |
| Inclacumab | 1 | 1.48 (0.06 – 36.30) | - |
| Methotrexate | 0 | - | - |
| Pexelizumab | 3 | 0.97 (0.57 – 1.67) | τ^2^:0.055; I^2^: 0% |
| Tocilizumab | 1 | 0.30 (0.01 – 7.43) | - |
| **Overall** | **10** | **0.82 (0.54 – 1.26)** | **τ^2^: 0.141; I^2^: 23%** |
| **P for interaction: 0.844** |  |  |  |
| **Only STEMI** | | | |
| Anakinra | 1 | 2.73 (0.13 – 56.96) | - |
| Canakinumab | 0 | - | - |
| Colchicine | 2 | 1.17 (0.79 – 1.75) | τ^2^:0.000; I^2^: 0%- |
| Cyclosporine | 1 | 0.59 (0.23 – 1.49) | - |
| Everolimus | 0 | - | - |
| Inclaclumab | 1 | 1.48 (0.06 – 36.30) | - |
| Methotrexate | 0 | - | - |
| Pexelizumab | 3 | 0.97 (0.57 – 1.67) | τ^2^:0.055; I^2^: 0% |
| Tocilizumab | 1 | - | - |
| **Overall** | **9** | **1.04 (0.80 – 1.36)** | **τ^2^:0.000; I^2^: 0%** |
| **P for interaction: 0.718** |  |  |  |
| **Only phase 3 trials** | | | |
| Anakinra | 0 | - | - |
| Canakinumab | 1 | 0.93 (0.72 – 1.20) | - |
| Colchicine | 4 | 0.60 (0.30 – 1.22) | τ^2^:0.325; I^2^: 69% |
| Cyclosporine | 1 | 0.59 (0.23 – 1.49) | - |
| Everolimus | 0 | - | - |
| Inclaclumab | 0 | - | - |
| Methotrexate | 1 | 0.94 (0.72 – 1.20) | - |
| Pexelizumab | 1 | 1.16 (0.73 – 1.83) | - |
| Tocilizumab | 0 | - | - |
| **Overall** | **8** | **0.91 (0.77 – 1.08)** | **τ^2^<0.001; I^2^: 41%** |
| **P for interaction: 0.515** |  |  |  |
| **Long-term follow-up** | | | |
| Anakinra | 2 | 2.80 (0.31 – 1.49) | τ^2^:0.000; I^2^: 0% |
| Canakinumab | 1 | - | - |
| Colchicine | 5 | 0.66 (0.36 – 1.23) | τ^2^:0.263; I^2^: 59% |
| Cyclosporine | 1 | - | - |
| Everolimus | 0 | - | - |
| Inclacumab | 0 | - | - |
| Methotrexate | 1 | 0.94 (0.56 – 1.57) | - |
| Pexelizumab | 0 | - | - |
| Tocilizumab | 1 | - | - |
| **Overall** | **11** | **0.91 (0.77 – 1.07)** | **τ^2^:0.000; I^2^: 5%** |
| **P for interaction: 0.838** |  |  |  |
| **Patients receiving PCI** | | | |
| Anakinra | 0 | - | - |
| Canakinumab | 0 | - | - |
| Colchicine | 4 | 0.63 (0.27 – 1.52) | τ^2^:0.463; I^2^: 67% |
| Cyclosporine | 1 | - | - |
| Everolimus | 0 | - | - |
| Inclacumab | 1 | 1.48 (0.06 – 36.30) | - |
| Methotrexate | 0 | - | - |
| Pexelizumab | 1 | 1.16 (0.73 – 1.83) | - |
| Tocilizumab | 1 | 0.30 (0.01 – 7.43) | - |
| **Overall** | **8** | **0.76 (0.45 – 1.26)** | **τ^2^: 0.215; I^2^: 39%** |
| **P for interaction: 0.540** |  |  |  |

Light blue cells highlight significant reduction with treatment, while light red cells highlight significant increase with treatment. Significant p-values for interaction are shown in red.
Abbreviations: CI, confidence interval; IRR, incidence rate ratio.

**Supplementary Table 21. Sensitivity analyses for serious infection or sepsis.**

| **Drug administered** | **Comparisons** | **IRR (95% CI)** | **Heterogeneity** |
| --- | --- | --- | --- |
| **Only acute phase** | | | |
| Anakinra | 2 | 0.99 (0.38 – 2.57) | τ^2^:0.000; I^2^: 0% |
| Canakinumab | 0 | - | - |
| Colchicine | 2 | 1.05 (0.68 – 1.63) | τ^2^:0.067; I^2^: 67% |
| Cyclosporine | 0 | - | - |
| Everolimus | 1 | 1.00 (0.25 – 4.00) | - |
| Inclacumab | 1 | 0.89 (0.52 – 1.52) | - |
| Methotrexate | 0 | - | - |
| Pexelizumab | 3 | 0.88 (0.65 – 1.18) | τ^2^:0.000; I^2^: 0% |
| Tocilizumab | 3 | 0.86 (0.29 – 2.53) | τ^2^:0.000; I^2^: 0% |
| **Overall** | **12** | **0.94 (0.80 – 1.11)** | **τ^2^: 0.000; I^2^: 0%** |
| **P for interaction: 0.991** |  |  |  |
| **Only STEMI** | | | |
| Anakinra | 2 | 0.99 (0.38 – 2.57) | τ^2^:0.000; I^2^: 0% |
| Canakinumab | 0 | - | - |
| Colchicine | 1 | 0.86 (0.65 – 1.15) | - |
| Cyclosporine | 0 | - | - |
| Everolimus | 1 | 1.00 (0.25 – 4.00) | - |
| Inclaclumab | 1 | 0.89 (0.52 – 1.52) | - |
| Methotrexate | 0 | - | - |
| Pexelizumab | 3 | 0.88 (0.65 – 1.18) | τ^2^:0.000; I^2^: 0% |
| Tocilizumab | 1 | - | - |
| **Overall** | **9** | **0.88 (0.73 – 1.06)** | **τ^2^:0.000; I^2^: 0%** |
| **P for interaction: 0.997** |  |  |  |
| **Only phase 3 trials** | | | |
| Anakinra | 0 | - | - |
| Canakinumab | 1 | 1.10 (0.97 – 1.25) | - |
| Colchicine | 4 | 0.98 (0.82 – 1.18) | τ^2^:0.004; I^2^: 28% |
| Cyclosporine | 0 | - | - |
| Everolimus | 0 | - | - |
| Inclaclumab | 0 | - | - |
| Methotrexate | 1 | 0.92 (0.71 – 1.19) | - |
| Pexelizumab | 1 | 0.90 (0.64 – 1.26) | - |
| Tocilizumab | 0 | - | - |
| **Overall** | **7** | **1.01 (0.91 – 1.12)** | **τ^2^: 0.003; I^2^: 12%** |
| **P for interaction: 0.466** |  |  |  |
| **Long-term follow-up** | | | |
| Anakinra | 2 | 1.10 (0.41 – 2.98) | τ^2^:0.000; I^2^: 0% |
| Canakinumab | 1 | 1.10 (0.97 – 1.25) | - |
| Colchicine | 4 | 0.98 (0.82 – 1.18) | τ^2^:0.004; I^2^: 28% |
| Cyclosporine | 0 | - | - |
| Everolimus | 0 | - | - |
| Inclacumab | 0 | - | - |
| Methotrexate | 1 | 0.92 (0.71 – 1.19) | - |
| Pexelizumab | 0 | - | - |
| Tocilizumab | 2 | 0.83 (0.27 – 2.53) | - |
| **Overall** | **10** | **1.02 (0.91 – 1.14)** | **τ^2^:0.028; I^2^: 0%** |
| **P for interaction: 0.722** |  |  |  |
| **Patients receiving PCI** | | | |
| Anakinra | 1 | 1.00 (0.14 – 7.10) | - |
| Canakinumab | 0 | - | - |
| Colchicine | 3 | 1.09 (0.70 – 1.68) | τ^2^:0.070; I^2^: 51% |
| Cyclosporine | 0 | - | - |
| Everolimus | 1 | 1.00 (0.25 – 4.00) | - |
| Inclacumab | 1 | 0.89 (0.52 – 1.52) | - |
| Methotrexate | 0 | - | - |
| Pexelizumab | 1 | 0.90 (0.64 – 1.26) | - |
| Tocilizumab | 1 | 1.36 (0.23 – 8.15) | - |
| **Overall** | **8** | **0.96 (0.81 – 1.15)** | **τ^2^: 0.000; I^2^: 0%** |
| **P for interaction: 0.984** |  |  |  |

Light blue cells highlight significant reduction with treatment, while light red cells highlight significant increase with treatment. Significant p-values for interaction are shown in red.
Abbreviations: CI, confidence interval; IRR, incidence rate ratio.

**Supplementary Table 22. Sensitivity analyses for cancer development.**

| **Drug administered** | **Comparisons** | **IRR (95% CI)** | **Heterogeneity** |
| --- | --- | --- | --- |
| **Only acute phase** | | | |
| Anakinra | 0 | - | - |
| Canakinumab | 0 | - | - |
| Colchicine | 1 | 0.94 (0.62 – 1.43) | - |
| Cyclosporine | 0 | - | - |
| Everolimus | 0 | - | - |
| Inclacumab | 0 | - | - |
| Methotrexate | 0 | - | - |
| Pexelizumab | 0 | - | - |
| Tocilizumab | 1 | 4.54 (0.22 – 94.50) | - |
| **Overall** | **2** | **0.98 (0.61 – 1.57)** | **τ^2^: 0.015; I^2^: 1%** |
| **P for interaction: 0.315** |  |  |  |
| **Only STEMI** | | | |
| Anakinra | 0 | - | - |
| Canakinumab | 0 | - | - |
| Colchicine | 0 | - | - |
| Cyclosporine | 0 | - | - |
| Everolimus | 0 | - | - |
| Inclaclumab | 0 | - | - |
| Methotrexate | 0 | - | - |
| Pexelizumab | 0 | - | - |
| Tocilizumab | 1 | 4.54 (0.22 – 94.50) | - |
| **Overall** | **1** | **4.54 (0.22 – 94.50)** | - |
| **P for interaction:** - |  |  |  |
| **Only phase 3 trials** | | | |
| Anakinra | 0 | - | - |
| Canakinumab | 1 | 0.93 (0.77 – 1.09) | - |
| Colchicine | 2 | 0.97 (0.78 – 1.21) | τ^2^:0.000; I^2^: 0% |
| Cyclosporine | 0 | - | - |
| Everolimus | 0 | - | - |
| Inclaclumab | 0 | - | - |
| Methotrexate | 1 | 1.12 (0.85 – 1.47) | - |
| Pexelizumab | 0 | - | - |
| Tocilizumab | 0 | - | - |
| **Overall** | **4** | **0.97 (0.87 – 1.09)** | **τ^2^: 0.000; I^2^: 0%** |
| **P for interaction: 0.526** |  |  |  |
| **Long-term follow-up** | | | |
| Anakinra | 0 | - | - |
| Canakinumab | 1 | 0.93 (0.79 – 1.09) | - |
| Colchicine | 2 | 0.97 (0.78 – 1.21) | τ^2^:0.000; I^2^: 0% |
| Cyclosporine | 0 | - | - |
| Everolimus | 0 | - | - |
| Inclacumab | 0 | - | - |
| Methotrexate | 1 | 1.12 (0.85 – 1.47) | - |
| Pexelizumab | 0 | - | - |
| Tocilizumab | 1 | 4.54 (0.22 – 94.50) | - |
| **Overall** | **5** | **0.97 (0.87 – 1.09)** | **τ^2^: 0.000; I^2^: 0%** |
| **P for interaction: 0.360** |  |  |  |
| **Patients receiving PCI** | | | |
| Anakinra | 0 | - | - |
| Canakinumab | 0 | - | - |
| Colchicine | 1 | 0.94 (0.62 – 1.43) | - |
| Cyclosporine | 0 | - | - |
| Everolimus | 0 | - | - |
| Inclacumab | 0 | - | - |
| Methotrexate | 0 | - | - |
| Pexelizumab | 0 | - | - |
| Tocilizumab | 1 | 4.54 (0.22 – 94.50) | - |
| **Overall** | **2** | **0.98 (0.61 – 1.57)** | **τ^2^: 0.015; I^2^: 1%** |
| **P for interaction: 0.315** |  |  |  |

Light blue cells highlight significant reduction with treatment, while light red cells highlight significant increase with treatment. Significant p-values for interaction are shown in red.
Abbreviations: CI, confidence interval; IRR, incidence rate ratio.

**Supplementary Table 23. Sensitivity analyses for pneumonia.**

| **Drug administered** | **Comparisons** | **IRR (95% CI)** | **Heterogeneity** |
| --- | --- | --- | --- |
| **Only acute phase** | | | |
| Anakinra | 1 | 0.33 (0.01 – 8.18) | - |
| Canakinumab | 0 | - | - |
| Colchicine | 1 | 2.33 (1.07 – 5.10) | - |
| Cyclosporine | 0 | - | - |
| Everolimus | 0 | - | - |
| Inclacumab | 0 | - | - |
| Methotrexate | 1 | 0.32 (0.01 – 7.80) | - |
| Pexelizumab | 0 | - | - |
| Tocilizumab | 0 | - | - |
| **Overall** | **3** | **1.19 (0.27 – 5.29)** | **τ^2^: 0.722; I^2^: 23%** |
| **P for interaction: 0.272** |  |  |  |
| **Only STEMI** | | | |
| Anakinra | 1 | 0.33 (0.01 – 8.18) | - |
| Canakinumab | 0 | - | - |
| Colchicine | 0 | - | - |
| Cyclosporine | 0 | - | - |
| Everolimus | 0 | - | - |
| Inclaclumab | 0 | - | - |
| Methotrexate | 1 | 0.32 (0.01 – 7.80) | - |
| Pexelizumab | 0 | - | - |
| Tocilizumab | 0 | - | - |
| **Overall** | **2** | **0.33 (0.03 – 3.13)** | **τ^2^:0.000; I^2^: 0%** |
| **P for interaction: 0.984** |  |  |  |
| **Only phase 3 trials** | | | |
| Anakinra | 0 | - | - |
| Canakinumab | 1 | 1.06 (0.85 – 1.32) | - |
| Colchicine | 3 | 1.47 (0.58 – 3.74) | τ^2^:0.404; I^2^: 69% |
| Cyclosporine | 0 | - | - |
| Everolimus | 0 | - | - |
| Inclaclumab | 0 | - | - |
| Methotrexate | 0 | - | - |
| Pexelizumab | 0 | - | - |
| Tocilizumab | 0 | - | - |
| **Overall** | **4** | **1.18 (0.74 – 1.89)** | **τ^2^: 0.122; I^2^: 53%** |
| **P for interaction: 0.506** |  |  |  |
| **Long-term follow-up** | | | |
| Anakinra | 0 | - | - |
| Canakinumab | 1 | 1.06 (0.85 – 1.32) | - |
| Colchicine | 3 | 1.47 (0.58 – 3.74) | τ^2^:0.404; I^2^: 69% |
| Cyclosporine | 0 | - | - |
| Everolimus | 0 | - | - |
| Inclacumab | 0 | - | - |
| Methotrexate | 0 | - | - |
| Pexelizumab | 0 | - | - |
| Tocilizumab | 0 | - | - |
| **Overall** | **4** | **1.18 (0.74 – 1.89)** | **τ^2^: 0.122; I^2^: 53%** |
| **P for interaction: 0.506** |  |  |  |
| **Patients receiving PCI** | | | |
| Anakinra | 1 | 0.33 (0.01 – 8.18) | - |
| Canakinumab | 0 | - | - |
| Colchicine | 2 | 2.45 (1.15 – 5.21) | τ^2^: 0.000; I^2^: 0% |
| Cyclosporine | 0 | - | - |
| Everolimus | 0 | - | - |
| Inclacumab | 0 | - | - |
| Methotrexate | 1 | 0.32 (0.01 – 7.80) | - |
| Pexelizumab | 0 | - | - |
| Tocilizumab | 0 | - | - |
| **Overall** | **4** | **2.00 (0.98 – 4.09)** | **τ^2^: 0.000; I^2^: 0%** |
| **P for interaction: 0.253** |  |  |  |

Light blue cells highlight significant reduction with treatment, while light red cells highlight significant increase with treatment. Significant p-values for interaction are shown in red.
Abbreviations: CI, confidence interval; IRR, incidence rate ratio.

**Supplementary Table 24. Sensitivity analyses for gastrointestinal adverse events.**

| **Drug administered** | **Comparisons** | **IRR (95% CI)** | **Heterogeneity** |
| --- | --- | --- | --- |
| **Only acute phase** | | | |
| Anakinra | 0 | - | - |
| Canakinumab | 0 | - | - |
| Colchicine | 5 | 2.32 (0.92 – 5.85) | τ^2^: 0.728; I^2^: 65% |
| Cyclosporine | 0 | - | - |
| Everolimus | 1 | 1.00 (0.14 – 7.10) | - |
| Inclacumab | 0 | - | - |
| Methotrexate | 1 | 0.95 (0.33 – 2.72) | - |
| Pexelizumab | 0 | - | - |
| Tocilizumab | 0 | - | - |
| **Overall** | **7** | **1.32 (0.99 – 1.75)** | **τ^2^<0.001; I^2^: 49%** |
| **P for interaction: 0.418** |  |  |  |
| **Only STEMI** | | | |
| Anakinra | 0 | - | - |
| Canakinumab | 0 | - | - |
| Colchicine | 3 | 5.30 (0.65 – 43.13) | τ^2^:2.559; I^2^: 81% |
| Cyclosporine | 0 | - | - |
| Everolimus | 1 | 1.00 (0.14 – 7.10) | - |
| Inclaclumab | 0 | - | - |
| Methotrexate | 1 | 0.95 (0.33 – 2.72) | - |
| Pexelizumab | 0 | - | - |
| Tocilizumab | 0 | - | - |
| **Overall** | **5** | **2.28 (0.68 – 7.62)** | **τ^2^:1.219; I^2^: 64%** |
| **P for interaction: 0.345** |  |  |  |
| **Only phase 3 trials** | | | |
| Anakinra | 0 | - | - |
| Canakinumab | 0 | - | - |
| Colchicine | 4 | 1.12 (0.93 – 1.35) | τ^2^:0.000; I^2^: 0% |
| Cyclosporine | 0 | - | - |
| Everolimus | 0 | - | - |
| Inclaclumab | 0 | - | - |
| Methotrexate | 1 | 1.23 (1.06 – 1.44) | - |
| Pexelizumab | 0 | - | - |
| Tocilizumab | 0 | - | - |
| **Overall** | **5** | **1.19 (1.05 – 1.34)** | **τ^2^: 0.000; I^2^: 0%** |
| **P for interaction: 0.433** |  |  |  |
| **Long-term follow-up** | | | |
| Anakinra | 0 | - | - |
| Canakinumab | 0 | - | - |
| Colchicine | 4 | 1.12 (0.93 – 1.36) | τ^2^:0.000; I^2^: 0% |
| Cyclosporine | 0 | - | - |
| Everolimus | 0 | - | - |
| Inclaclumab | 0 | - | - |
| Methotrexate | 1 | 1.23 (1.06 – 1.44) | - |
| Pexelizumab | 0 | - | - |
| Tocilizumab | 0 | - | - |
| **Overall** | **5** | **1.19 (1.05 – 1.34)** | **τ^2^: 0.000; I^2^: 0%** |
| **P for interaction: 0.433** |  |  |  |
| **Patients receiving PCI** | | | |
| Anakinra | 0 | - | - |
| Canakinumab | 0 | - | - |
| Colchicine | 6 | 1.22 (0.99 – 1.51) | τ^2^<0.001; I^2^: 59% |
| Cyclosporine | 0 | - | - |
| Everolimus | 1 | 1.00 (0.14 – 7.10) | - |
| Inclacumab | 0 | - | - |
| Methotrexate | 1 | 0.95 (0.33 – 2.72) | - |
| Pexelizumab | 0 | - | - |
| Tocilizumab | 0 | - | - |
| **Overall** | **8** | **1.21 (0.99 – 1.49)** | **τ^2^<0.001; I^2^: 44%** |
| **P for interaction: 0.884** |  |  |  |

Light blue cells highlight significant reduction with treatment, while light red cells highlight significant increase with treatment. Significant p-values for interaction are shown in red.
Abbreviations: CI, confidence interval; IRR, incidence rate ratio.

**Supplementary Table 25. Leave-one-out analysis.**

| **Study omitted** | **IRR (95% CI)** | **P-value** | **τ^2^** | **I^2^** |
| --- | --- | --- | --- | --- |
| **MACE** |  |  |  |  |
| **Pooled estimate** | **0.92 (0.86 – 0.98)** | **0.010** | **<0.001** | **39%** |
| **COMPLY, 2003** | 0.92 (0.86 – 0.98) | 0.010 | 0.001 | 42% |
| **COMMA, 2003** | 0.92 (0.86 – 0.98) | 0.013 | 0.001 | 43% |
| **APEX AMI, 2007** | 0.91 (0.85 – 0.97) | 0.008 | 0.003 | 40% |
| **Piot et al., 2008** | 0.92 (0.86 – 0.98) | 0.010 | 0.001 | 39% |
| **Ghaffari et al., 2012** | 0.92 (0.86 – 0.98) | 0.010 | 0.003 | 39% |
| **VCU-ART2, 2013** | 0.92 (0.86 – 0.98) | 0.010 | 0.003 | 39% |
| **SELECT-ACS, 2013** | 0.92 (0.86 – 0.98) | 0.010 | 0.003 | 39% |
| **MRC-ILA, 2014** | 0.92 (0.86 – 0.98) | 0.007 | 0.001 | 33% |
| **CIRCUS, 2015** | 0.91 (0.85 – 0.97) | 0.005 | 0.001 | 40% |
| **CYCLE, 2015** | 0.92 (0.86 – 0.98) | 0.010 | 0.001 | 42% |
| **Deftereos et al., 2015** | 0.92 (0.86 – 0.98) | 0.010 | 0.001 | 39% |
| **COLIN, 2016** | 0.92 (0.86 – 0.98) | 0.010 | 0.001 | 39% |
| **Kleveland et al., 2016** | 0.92 (0.87 – 0.98) | 0.010 | 0.001 | 42% |
| **CANTOS, 2017** | 0.93 (0.85 – 1.01) | 0.070 | 0.003 | 42% |
| **STAT-MI, 2017** | 0.92 (0.86 – 0.98) | 0.007 | 0.003 | 31% |
| **TETHYS, 2017** | 0.92 (0.86 – 0.98) | 0.010 | 0.001 | 39% |
| **CIRT, 2018** | 0.91 (0.85 – 0.98) | 0.007 | 0.001 | 41% |
| **COLCOT, 2019** | 0.92 (0.86 – 0.99) | 0.024 | 0.002 | 42% |
| **VCUART3, 2019** | 0.92 (0.86 – 0.98) | 0.011 | 0.001 | 42% |
| **LoDoCo-MI, 2019** | 0.92 (0.86 – 0.98) | 0.010 | 0.001 | 41% |
| **LoDoCo2, 2020** | 0.93 (0.88 – 0.99) | 0.030 | <0.001 | 35% |
| **Australian COPS, 2020** | 0.93 (0.87 – 0.99) | 0.016 | <0.001 | 37% |
| **ASSAIL-MI, 2021** | 0.92 (0.86 – 0.98) | 0.011 | 0.001 | 43% |
| **Akrami et al., 2021** | 0.93 (0.87 – 0.99) | 0.015 | <0.001 | 25% |
| **CLEVER-ACS, 2022** | 0.92 (0.86 – 0.98) | 0.010 | 0.001 | 39% |
| **PodCAST-PCI, 2022** | 0.92 (0.86 – 0.98) | 0.012 | 0.001 | 42% |
| **COVERT-MI, 2023** | 0.92 (0.86 – 0.98) | 0.015 | 0.001 | 42% |
| **CLEAR SYERGY (OASIS 9), 2024** | 0.91 (0.85 – 0.98) | 0.018 | 0.003 | 42% |
| **Serious adverse events** |  |  |  |  |
| **Pooled estimate** | **0.99 (0.94 – 1.04)** | **0.623** | **0.000** | **0%** |
| **COMPLY, 2003** | 0.99 (0.94 – 1.04) | 0.623 | 0.000 | 0% |
| **COMMA, 2003** | 0.99 (0.94 – 1.04) | 0.623 | 0.000 | 0% |
| **APEX AMI, 2007** | 0.99 (0.94 – 1.04) | 0.688 | 0.000 | 0% |
| **Piot et al., 2008** | 0.99 (0.94 – 1.04) | 0.658 | 0.000 | 0% |
| **Ghaffari et al., 2012** | 0.99 (0.94 – 1.04) | 0.623 | 0.000 | 0% |
| **VCU-ART2, 2013** | 0.99 (0.94 – 1.04) | 0.623 | 0.000 | 0% |
| **SELECT-ACS, 2013** | 0.99 (0.94 – 1.03) | 0.497 | 0.000 | 0% |
| **MRC-ILA, 2014** | 0.99 (0.94 – 1.04) | 0.623 | 0.000 | 0% |
| **CIRCUS, 2015** | 0.99 (0.94 – 1.04) | 0.623 | 0.000 | 0% |
| **CYCLE, 2015** | 0.99 (0.94 – 1.04) | 0.616 | 0.000 | 0% |
| **Deftereos et al., 2015** | 0.99 (0.94 – 1.04) | 0.623 | 0.000 | 0% |
| **COLIN, 2016** | 0.99 (0.94 – 1.04) | 0.623 | 0.000 | 0% |
| **Kleveland et al., 2016** | 0.99 (0.94 – 1.04) | 0.640 | 0.000 | 0% |
| **CANTOS, 2017** | 0.99 (0.92 – 1.06) | 0.690 | 0.000 | 0% |
| **STAT-MI, 2017** | 0.99 (0.94 – 1.04) | 0.623 | 0.000 | 0% |
| **TETHYS, 2017** | 0.99 (0.94 – 1.05) | 0.623 | 0.000 | 0% |
| **CIRT, 2018** | 0.98 (0.92 – 1.03) | 0.402 | 0.000 | 0% |
| **COLCOT, 2019** | 0.99 (0.94 – 1.05) | 0.782 | 0.000 | 0% |
| **VCUART3, 2019** | 0.99 (0.94 – 1.04) | 0.623 | 0.000 | 0% |
| **LoDoCo-MI, 2019** | 0.99 (0.94 – 1.04) | 0.640 | 0.000 | 0% |
| **LoDoCo2, 2020** | 0.99 (0.94 – 1.04) | 0.623 | 0.000 | 0% |
| **Australian COPS, 2020** | 0.99 (0.94 – 1.04) | 0.685 | 0.000 | 0% |
| **ASSAIL-MI, 2021** | 0.99 (0.94 – 1.04) | 0.601 | 0.000 | 0% |
| **Akrami et al., 2021** | 0.99 (0.94 – 1.04) | 0.623 | 0.000 | 0% |
| **CLEVER-ACS, 2022** | 0.99 (0.94 – 1.04) | 0.562 | 0.000 | 0% |
| **PodCAST-PCI, 2022** | 0.99 (0.94 – 1.04) | 0.623 | 0.000 | 0% |
| **COVERT-MI, 2023** | 0.99 (0.94 – 1.04) | 0.862 | 0.000 | 0% |
| **CLEAR SYERGY (OASIS 9), 2024** | 1.00 (0.95 – 1.05) | 0.862 | 0.000 | 0% |
| **All-cause death** |  |  |  |  |
| **Pooled estimate** | **1.01 (0.92 – 1.10)** | **0.871** | **0.002** | **0%** |
| **COMPLY, 2003** | 1.00 (0.91 – 1.10) | 0.933 | 0.002 | 0% |
| **COMMA, 2003** | 1.03 (0.93 – 1.13) | 0.610 | 0.003 | 0% |
| **APEX AMI, 2007** | 0.99 (0.90 – 1.09) | 0.857 | 0.001 | 0% |
| **Piot et al., 2008** | 1.01 (0.92 – 1.10) | 0.869 | 0.002 | 0% |
| **Ghaffari et al., 2012** | 1.01 (0.92 – 1.11) | 0.834 | 0.002 | 0% |
| **VCU-ART2, 2013** | 1.01 (0.92 – 1.11) | 0.847 | 0.002 | 0% |
| **SELECT-ACS, 2013** | 1.01 (0.92 – 1.10) | 0.921 | 0.002 | 0% |
| **MRC-ILA, 2014** | 1.00 (0.92 – 1.10) | 0.943 | 0.001 | 0% |
| **CIRCUS, 2015** | 1.01 (0.92 – 1.11) | 0.891 | 0.002 | 0% |
| **CYCLE, 2015** | 1.00 (0.91 – 1.09) | 0.967 | 0.001 | 0% |
| **Deftereos et al., 2015** | 1.01 (0.92 – 1.11) | 0.869 | 0.002 | 0% |
| **COLIN, 2016** | 1.01 (0.92 – 1.10) | 0.871 | 0.002 | 0% |
| **Kleveland et al., 2016** | 1.01 (0.92 – 1.10) | 0.871 | 0.002 | 0% |
| **CANTOS, 2017** | 1.05 (0.94 – 1.17) | 0.386 | 0.000 | 0% |
| **STAT-MI, 2017** | 1.01 (0.92 – 1.10) | 0.876 | 0.002 | 0% |
| **TETHYS, 2017** | 1.05 (0.92 – 1.10) | 0.920 | 0.002 | 0% |
| **CIRT, 2018** | 0.99 (0.90 – 1.08) | 0.742 | 0.000 | 0% |
| **COLCOT, 2019** | 1.01 (0.92 – 1.12) | 0.785 | 0.003 | 0% |
| **VCUART3, 2019** | 1.01 (0.92 – 1.11) | 0.834 | 0.002 | 0% |
| **LoDoCo-MI, 2019** | 1.01 (0.92 – 1.10) | 0.870 | 0.002 | 0% |
| **LoDoCo2, 2020** | 0.99 (0.91 – 1.07) | 0.745 | 0.000 | 0% |
| **Australian COPS, 2020** | 1.00 (0.92 – 1.09) | 0.984 | 0.001 | 0% |
| **ASSAIL-MI, 2021** | 1.01 (0.92 – 1.10) | 0.871 | 0.001 | 0% |
| **Akrami et al., 2021** | 1.00 (0.92 – 1.10) | 0.928 | 0.002 | 0% |
| **CLEVER-ACS, 2022** | 1.01 (0.92 – 1.10) | 0.871 | 0.002 | 0% |
| **PodCAST-PCI, 2022** | 1.01 (0.92 – 1.10) | 0.871 | 0.002 | 0% |
| **COVERT-MI, 2023** | 1.01 (0.92 – 1.10) | 0.480 | 0.004 | 0% |
| **CLEAR SYERGY (OASIS 9), 2024** | 1.04 (0.93 – 1.16) | 0.480 | 0.004 | 0% |
| **Cardiovascular death** |  |  |  |  |
| **Pooled estimate** | **0.96 (0.84 – 1.09)** | **0.477** | **0.000** | **0%** |
| **COMPLY, 2003** | 0.96 (0.84 – 1.09) | 0.477 | 0.000 | 0% |
| **COMMA, 2003** | 0.96 (0.84 – 1.09) | 0.477 | 0.000 | 0% |
| **APEX AMI, 2007** | 0.96 (0.84 – 1.09) | 0.477 | 0.000 | 0% |
| **Piot et al., 2008** | 0.96 (0.84 – 1.09) | 0.479 | 0.000 | 0% |
| **Ghaffari et al., 2012** | 0.96 (0.84 – 1.09) | 0.477 | 0.000 | 0% |
| **VCU-ART2, 2013** | 0.96 (0.84 – 1.09) | 0.506 | 0.000 | 0% |
| **SELECT-ACS, 2013** | 0.96 (0.84 – 1.09) | 0.477 | 0.000 | 0% |
| **MRC-ILA, 2014** | 0.96 (0.84 – 1.09) | 0.477 | 0.000 | 0% |
| **CIRCUS, 2015** | 0.96 (0.84 – 1.09) | 0.508 | 0.000 | 0% |
| **CYCLE, 2015** | 0.95 (0.83 – 1.08) | 0.391 | 0.000 | 0% |
| **Deftereos et al., 2015** | 0.96 (0.84 – 1.09) | 0.477 | 0.000 | 0% |
| **COLIN, 2016** | 0.96 (0.84 – 1.09) | 0.477 | 0.000 | 0% |
| **Kleveland et al., 2016** | 0.95 (0.84 – 1.08) | 0.461 | 0.000 | 0% |
| **CANTOS, 2017** | 1.04 (0.87 – 1.24) | 0.659 | 0.000 | 0% |
| **STAT-MI, 2017** | 0.96 (0.84 – 1.08) | 0.473 | 0.000 | 0% |
| **TETHYS, 2017** | 0.96 (0.84 – 1.09) | 0.477 | 0.000 | 0% |
| **CIRT, 2018** | 0.94 (0.82 – 1.07) | 0.340 | 0.000 | 0% |
| **COLCOT, 2019** | 0.97 (0.84 – 1.11) | 0.645 | 0.000 | 0% |
| **VCUART3, 2019** | 0.96 (0.84 – 1.09) | 0.477 | 0.000 | 0% |
| **LoDoCo-MI, 2019** | 0.96 (0.84 – 1.09) | 0.377 | 0.000 | 0% |
| **LoDoCo2, 2020** | 0.97 (0.84 – 1.12) | 0.683 | 0.002 | 0% |
| **Australian COPS, 2020** | 0.95 (0.84 – 1.08) | 0.444 | 0.000 | 0% |
| **ASSAIL-MI, 2021** | 0.96 (0.84 – 1.09) | 0.477 | 0.000 | 0% |
| **Akrami et al., 2021** | 0.95 (0.84 – 1.08) | 0.436 | 0.000 | 0% |
| **CLEVER-ACS, 2022** | 0.96 (0.84 – 1.09) | 0.477 | 0.000 | 0% |
| **PodCAST-PCI, 2022** | 0.96 (0.84 – 1.09) | 0.477 | 0.000 | 0% |
| **COVERT-MI, 2023** | 0.96 (0.84 – 1.09) | 0.477 | 0.000 | 0% |
| **CLEAR SYERGY (OASIS 9), 2024** | 0.93 (0.80 – 1.08) | 0.331 | 0.000 | 0% |
| **Myocardial infarction** |  |  |  |  |
| **Pooled estimate** | **0.91 (0.81 – 1.02)** | **0.118** | **0.007** | **18%** |
| **COMPLY, 2003** | 0.91 (0.81 – 1.03) | 0.132 | 0.007 | 22% |
| **COMMA, 2003** | 0.91 (0.81 – 1.03) | 0.132 | 0.007 | 21% |
| **APEX AMI, 2007** | 0.87 (0.79 – 0.96) | 0.006 | 0.000 | 0% |
| **Piot et al., 2008** | 0.91 (0.81 – 1.02) | 0.118 | 0.007 | 18% |
| **Ghaffari et al., 2012** | 0.91 (0.81 – 1.02) | 0.118 | 0.007 | 18% |
| **VCU-ART2, 2013** | 0.91 (0.81 – 1.02) | 0.111 | 0.007 | 20% |
| **SELECT-ACS, 2013** | 0.91 (0.81 – 1.02) | 0.089 | 0.006 | 14% |
| **MRC-ILA, 2014** | 0.90 (0.81 – 1.01) | 0.080 | 0.006 | 9% |
| **CIRCUS, 2015** | 0.92 (0.82 – 1.04) | 0.172 | 0.008 | 19% |
| **CYCLE, 2015** | 0.91 (0.81 – 1.02) | 0.118 | 0.007 | 18% |
| **Deftereos et al., 2015** | 0.91 (0.81 – 1.03) | 0.118 | 0.007 | 18% |
| **COLIN, 2016** | 0.91 (0.81 – 1.03) | 0.126 | 0.007 | 21% |
| **Kleveland et al., 2016** | 0.92 (0.81 – 1.03) | 0.138 | 0.007 | 17% |
| **CANTOS, 2017** | 0.94 (0.80 – 1.09) | 0.396 | 0.015 | 19% |
| **STAT-MI, 2017** | 0.91 (0.81 – 1.02) | 0.106 | 0.007 | 19% |
| **TETHYS, 2017** | 0.91 (0.81 – 1.02) | 0.106 | 0.007 | 19% |
| **CIRT, 2018** | 0.90 (0.79 – 1.04) | 0.141 | 0.011 | 20% |
| **COLCOT, 2019** | 0.92 (0.80 – 1.06) | 0.228 | 0.014 | 22% |
| **VCUART3, 2019** | 0.91 (0.81 – 1.02) | 0.118 | 0.007 | 18% |
| **LoDoCo-MI, 2019** | 0.91 (0.81 – 1.02) | 0.118 | 0.007 | 18% |
| **LoDoCo2, 2020** | 0.94 (0.84 – 1.05) | 0.283 | 0.004 | 11% |
| **Australian COPS, 2020** | 0.91 (0.81 – 1.02) | 0.119 | 0.007 | 22% |
| **ASSAIL-MI, 2021** | 0.92 (0.81 – 1.03) | 0.135 | 0.007 | 14% |
| **Akrami et al., 2021** | 0.91 (0.81 – 1.03) | 0.132 | 0.007 | 17% |
| **CLEVER-ACS, 2022** | 0.91 (0.81 – 1.03) | 0.124 | 0.007 | 21% |
| **PodCAST-PCI, 2022** | 0.91 (0.81 – 1.03) | 0.118 | 0.007 | 18% |
| **COVERT-MI, 2023** | 0.91 (0.81 – 1.03) | 0.118 | 0.007 | 18% |
| **CLEAR SYERGY (OASIS 9), 2024** | 0.92 (0.79 – 1.06) | 0.229 | 0.014 | 22% |
| **Heart failure** |  |  |  |  |
| **Pooled estimate** | **0.97 (0.85 – 1.12)** | **0.713** | **0.000** | **0%** |
| **COMPLY, 2003** | 0.97 (0.84 – 1.12) | 0.699 | 0.000 | 0% |
| **COMMA, 2003** | 0.96 (0.84 – 1.11) | 0.608 | 0.000 | 0% |
| **APEX AMI, 2007** | 0.97 (0.82 – 1.15) | 0.708 | 0.000 | 0% |
| **Piot et al., 2008** | 0.98 (0.86 – 1.13) | 0.797 | 0.000 | 0% |
| **Ghaffari et al., 2012** | 0.98 (0.85 – 1.12) | 0.723 | 0.000 | 0% |
| **VCU-ART2, 2013** | 0.98 (0.85 – 1.12) | 0.771 | 0.000 | 0% |
| **SELECT-ACS, 2013** | 0.97 (0.85 – 1.12) | 0.693 | 0.000 | 0% |
| **MRC-ILA, 2014** | 0.97 (0.85 – 1.12) | 0.713 | 0.000 | 0% |
| **CIRCUS, 2015** | 0.97 (0.83 – 1.13) | 0.67 | 0.000 | 0% |
| **CYCLE, 2015** | 0.98 (0.85 – 1.13) | 0.771 | 0.000 | 0% |
| **Deftereos et al., 2015** | 0.97 (0.85 – 1.12) | 0.713 | 0.000 | 0% |
| **COLIN, 2016** | 0.97 (0.85 – 1.12) | 0.714 | 0.000 | 0% |
| **Kleveland et al., 2016** | 0.97 (0.85 – 1.12) | 0.713 | 0.000 | 0% |
| **CANTOS, 2017** | 0.97 (0.85 – 1.12) | 0.713 | 0.000 | 0% |
| **STAT-MI, 2017** | 0.97 (0.85 – 1.12) | 0.713 | 0.000 | 0% |
| **TETHYS, 2017** | 0.97 (0.85 – 1.12) | 0.713 | 0.000 | 0% |
| **CIRT, 2018** | 0.98 (0.85 – 1.14) | 0.834 | 0.000 | 0% |
| **COLCOT, 2019** | 0.95 (0.83 – 1.10) | 0.508 | 0.000 | 0% |
| **VCUART3, 2019** | 0.97 (0.85 – 1.12) | 0.713 | 0.000 | 0% |
| **LoDoCo-MI, 2019** | 0.97 (0.85 – 1.12) | 0.713 | 0.000 | 0% |
| **LoDoCo2, 2020** | 0.97 (0.85 – 1.12) | 0.713 | 0.000 | 0% |
| **Australian COPS, 2020** | 0.97 (0.85 – 1.12) | 0.713 | 0.000 | 0% |
| **ASSAIL-MI, 2021** | 0.97 (0.85 – 1.12) | 0.713 | 0.000 | 0% |
| **Akrami et al., 2021** | 0.98 (0.85 – 1.12) | 0.733 | 0.000 | 0% |
| **CLEVER-ACS, 2022** | 0.97 (0.85 – 1.12) | 0.713 | 0.000 | 0% |
| **PodCAST-PCI, 2022** | 0.97 (0.85 – 1.12) | 0.713 | 0.000 | 0% |
| **COVERT-MI, 2023** | 0.99 (0.86 – 1.14) | 0.909 | 0.000 | 0% |
| **CLEAR SYERGY (OASIS 9), 2024** | 0.97 (0.85 – 1.12) | 0.713 | 0.000 | 0% |
| **Revascularization** |  |  |  |  |
| **Pooled estimate** | **0.83 (0.69 – 1.00)** | **0.052** | **0.045** | **54%** |
| **COMPLY, 2003** | 0.81 (0.66 – 0.98) | 0.027 | 0.043 | 54% |
| **COMMA, 2003** | 0.84 (0.69 – 1.02) | 0.079 | 0.049 | 58% |
| **APEX AMI, 2007** | 0.83 (0.69 – 1.00) | 0.052 | 0.045 | 54% |
| **Piot et al., 2008** | 0.83 (0.69 – 1.00) | 0.052 | 0.045 | 54% |
| **Ghaffari et al., 2012** | 0.83 (0.69 – 1.00) | 0.052 | 0.045 | 54% |
| **VCU-ART2, 2013** | 0.83 (0.69 – 1.00) | 0.053 | 0.045 | 58% |
| **SELECT-ACS, 2013** | 0.80 (0.67 – 0.97) | 0.020 | 0.039 | 52% |
| **MRC-ILA, 2014** | 0.80 (0.65 – 0.98) | 0.033 | 0.047 | 53% |
| **CIRCUS, 2015** | 0.83 (0.69 – 1.00) | 0.052 | 0.045 | 54% |
| **CYCLE, 2015** | 0.83 (0.69 – 1.00) | 0.052 | 0.045 | 54% |
| **Deftereos et al., 2015** | 0.83 (0.69 – 1.00) | 0.052 | 0.045 | 54% |
| **COLIN, 2016** | 0.83 (0.69 – 1.00) | 0.052 | 0.045 | 54% |
| **Kleveland et al., 2016** | 0.83 (0.69 – 1.00) | 0.052 | 0.045 | 54% |
| **CANTOS, 2017** | 0.87 (0.72 – 1.05) | 0.148 | 0.037 | 48% |
| **STAT-MI, 2017** | 0.83 (0.69 – 1.00) | 0.052 | 0.045 | 54% |
| **TETHYS, 2017** | 0.83 (0.69 – 1.00) | 0.052 | 0.045 | 54% |
| **CIRT, 2018** | 0.83 (0.67 – 1.03) | 0.088 | 0.059 | 58% |
| **COLCOT, 2019** | 0.87 (0.74 – 1.04) | 0.118 | 0.028 | 46% |
| **VCUART3, 2019** | 0.83 (0.69 – 1.00) | 0.052 | 0.045 | 54% |
| **LoDoCo-MI, 2019** | 0.83 (0.69 – 1.00) | 0.052 | 0.045 | 54% |
| **LoDoCo2, 2020** | 0.84 (0.67 – 1.05) | 0.127 | 0.062 | 56% |
| **Australian COPS, 2020** | 0.85 (0.71 – 1.02) | 0.084 | 0.040 | 50% |
| **ASSAIL-MI, 2021** | 0.83 (0.69 – 1.00) | 0.052 | 0.045 | 54% |
| **Akrami et al., 2021** | 0.83 (0.69 – 1.00) | 0.052 | 0.045 | 54% |
| **CLEVER-ACS, 2022** | 0.83 (0.69 – 1.00) | 0.052 | 0.045 | 54% |
| **PodCAST-PCI, 2022** | 0.83 (0.69 – 1.00) | 0.052 | 0.045 | 54% |
| **COVERT-MI, 2023** | 0.83 (0.69 – 1.00) | 0.052 | 0.045 | 54% |
| **CLEAR SYERGY (OASIS 9), 2024** | 0.80 (0.65 – 1.00) | 0.046 | 0.054 |  |
| **Stroke** |  |  |  |  |
| **Pooled estimate** | **0.91 (0.77 – 1.07)** | **0.258** | **0.000** | **5%** |
| **COMPLY, 2003** | 0.92 (0.78 – 1.09) | 0.346 | 0.000 | 5% |
| **COMMA, 2003** | 0.91 (0.77 – 1.07) | 0.246 | 0.000 | 11% |
| **APEX AMI, 2007** | 0.84 (0.68 – 1.05) | 0.134 | 0.021 | 4% |
| **Piot et al., 2008** | 0.91 (0.77 – 1.07) | 0.258 | 0.000 | 5% |
| **Ghaffari et al., 2012** | 0.91 (0.77 – 1.07) | 0.258 | 0.000 | 5% |
| **VCU-ART2, 2013** | 0.91 (0.77 – 1.07) | 0.258 | 0.000 | 5% |
| **SELECT-ACS, 2013** | 0.91 (0.77 – 1.07) | 0.252 | 0.000 | 11% |
| **MRC-ILA, 2014** | 0.91 (0.80 – 1.07) | 0.244 | 0.000 | 9% |
| **CIRCUS, 2015** | 0.92 (0.78 – 1.09) | 0.343 | 0.000 | 6% |
| **CYCLE, 2015** | 0.91 (0.77 – 1.07) | 0.258 | 0.000 | 5% |
| **Deftereos et al., 2015** | 0.91 (0.77 – 1.07) | 0.258 | 0.000 | 5% |
| **COLIN, 2016** | 0.91 (0.77 – 1.07) | 0.258 | 0.000 | 5% |
| **Kleveland et al., 2016** | 0.91 (0.77 – 1.07) | 0.258 | 0.000 | 5% |
| **CANTOS, 2017** | 0.83 (0.62 – 1.11) | 0.205 | 0.062 | 11% |
| **STAT-MI, 2017** | 0.91 (0.77 – 1.07) | 0.258 | 0.000 | 5% |
| **TETHYS, 2017** | 0.91 (0.77 – 1.07) | 0.258 | 0.000 | 5% |
| **CIRT, 2018** | 0.86 (0.67 – 1.10) | 0.218 | 0.040 | 12% |
| **COLCOT, 2019** | 0.94 (0.80 – 1.11) | 0.483 | 0.000 | 0% |
| **VCUART3, 2019** | 0.91 (0.77 – 1.07) | 0.243 | 0.000 | 9% |
| **LoDoCo-MI, 2019** | 0.91 (0.77 – 1.07) | 0.258 | 0.000 | 5% |
| **LoDoCo2, 2020** | 0.93 (0.79 – 1.10) | 0.405 | 0.000 | 5% |
| **Australian COPS, 2020** | 0.92 (0.78 – 1.08) | 0.318 | 0.000 | 2% |
| **ASSAIL-MI, 2021** | 0.91 (0.77 – 1.08) | 0.273 | 0.000 | 9% |
| **Akrami et al., 2021** | 0.91 (0.77 – 1.07) | 0.258 | 0.000 | 5% |
| **CLEVER-ACS, 2022** | 0.91 (0.77 – 1.07) | 0.258 | 0.000 | 5% |
| **PodCAST-PCI, 2022** | 0.91 (0.77 – 1.07) | 0.258 | 0.000 | 5% |
| **COVERT-MI, 2023** | 0.91 (0.77 – 1.07) | 0.244 | 0.000 | 11% |
| **CLEAR SYERGY (OASIS 9), 2024** | 0.86 (0.72 – 1.04) | 0.119 | 0.002 | 0% |
| **Serious infection or sepsis** | | | | |
| **Pooled estimate** | **0.99 (0.90 – 1.11)** | **0.989** | **0.003** | **0%** |
| **COMPLY, 2003** | 1.01 (0.91 – 1.11) | 0.905 | 0.003 | 0% |
| **COMMA, 2003** | 1.00 (0.90 – 1.11) | 0.978 | 0.003 | 0% |
| **APEX AMI, 2007** | 1.01 (0.91 – 1.12) | 0.869 | 0.003 | 0% |
| **Piot et al., 2008** | 0.99 (0.90 – 1.11) | 0.989 | 0.003 | 0% |
| **Ghaffari et al., 2012** | 0.99 (0.90 – 1.11) | 0.989 | 0.003 | 0% |
| **VCU-ART2, 2013** | 1.00 (0.90 – 1.11) | 0.987 | 0.003 | 0% |
| **SELECT-ACS, 2013** | 1.00 (0.91 – 1.11) | 0.955 | 0.003 | 0% |
| **MRC-ILA, 2014** | 1.00 (0.90 – 1.10) | 0.965 | 0.003 | 0% |
| **CIRCUS, 2015** | 0.99 (0.90 – 1.11) | 0.989 | 0.003 | 0% |
| **CYCLE, 2015** | 0.99 (0.90 – 1.11) | 0.989 | 0.003 | 0% |
| **Deftereos et al., 2015** | 0.99 (0.90 – 1.11) | 0.989 | 0.003 | 0% |
| **COLIN, 2016** | 0.99 (0.90 – 1.11) | 0.989 | 0.003 | 0% |
| **Kleveland et al., 2016** | 1.00 (0.91 – 1.11) | 0.964 | 0.003 | 0% |
| **CANTOS, 2017** | 0.94 (0.84 – 1.06) | 0.339 | 0.000 | 0% |
| **STAT-MI, 2017** | 1.00 (0.90 – 1.11) | 0.983 | 0.003 | 0% |
| **TETHYS, 2017** | 0.99 (0.90 – 1.11) | 0.989 | 0.003 | 0% |
| **CIRT, 2018** | 1.01 (0.91 – 1.13) | 0.835 | 0.003 | 0% |
| **COLCOT, 2019** | 0.98 (0.88 – 1.09) | 0.688 | 0.004 | 0% |
| **VCUART3, 2019** | 1.00 (0.90 – 1.11) | 0.985 | 0.003 | 0% |
| **LoDoCo-MI, 2019** | 1.00 (0.90 – 1.11) | 0.989 | 0.003 | 0% |
| **LoDoCo2, 2020** | 1.00 (0.90 – 1.13) | 0.946 | 0.004 | 0% |
| **Australian COPS, 2020** | 1.00 (0.90 – 1.10) | 0.954 | 0.003 | 0% |
| **ASSAIL-MI, 2021** | 1.00 (0.90 – 1.10) | 0.967 | 0.003 | 0% |
| **Akrami et al., 2021** | 0.99 (0.90 – 1.11) | 0.989 | 0.003 | 0% |
| **CLEVER-ACS, 2022** | 1.00 (0.90 – 1.11) | 0.985 | 0.003 | 0% |
| **PodCAST-PCI, 2022** | 1.00 (0.90 – 1.11) | 0.689 | 0.001 | 0% |
| **COVERT-MI, 2023** | 1.00 (0.90 – 1.11) | 0.689 | 0.001 | 0% |
| **CLEAR SYERGY (OASIS 9), 2024** | 1.02 (0.93 – 1.13) | 0.656 | 0.001 | 0% |
| **Cancer development** | | | | |
| **Pooled estimate** | **0.97 (0.87 – 1.09)** | **0.665** | **0.000** | **0%** |
| **COMPLY, 2003** | 0.97 (0.87 – 1.09) | 0.665 | 0.000 | 0% |
| **COMMA, 2003** | 0.97 (0.87 – 1.09) | 0.665 | 0.000 | 0% |
| **APEX AMI, 2007** | 0.97 (0.87 – 1.09) | 0.665 | 0.000 | 0% |
| **Piot et al., 2008** | 0.97 (0.87 – 1.09) | 0.665 | 0.000 | 0% |
| **Ghaffari et al., 2012** | 0.97 (0.87 – 1.09) | 0.665 | 0.000 | 0% |
| **VCU-ART2, 2013** | 0.97 (0.87 – 1.09) | 0.665 | 0.000 | 0% |
| **SELECT-ACS, 2013** | 0.97 (0.87 – 1.09) | 0.665 | 0.000 | 0% |
| **MRC-ILA, 2014** | 0.97 (0.87 – 1.09) | 0.665 | 0.000 | 0% |
| **CIRCUS, 2015** | 0.97 (0.87 – 1.09) | 0.665 | 0.000 | 0% |
| **CYCLE, 2015** | 0.97 (0.87 – 1.09) | 0.665 | 0.000 | 0% |
| **Deftereos et al., 2015** | 0.97 (0.87 – 1.09) | 0.665 | 0.000 | 0% |
| **COLIN, 2016** | 0.97 (0.87 – 1.09) | 0.665 | 0.000 | 0% |
| **Kleveland et al., 2016** | 0.97 (0.87 – 1.09) | 0.665 | 0.000 | 0% |
| **CANTOS, 2017** | 1.03 (0.87 – 1.22) | 0.741 | 0.000 | 0% |
| **STAT-MI, 2017** | 0.97 (0.87 – 1.09) | 0.665 | 0.000 | 0% |
| **TETHYS, 2017** | 0.97 (0.87 – 1.09) | 0.665 | 0.000 | 0% |
| **CIRT, 2018** | 0.95 (0.83 – 1.08) | 0.400 | 0.000 | 0% |
| **COLCOT, 2019** | 0.98 (0.87 – 1.10) | 0.713 | 0.000 | 0% |
| **VCUART3, 2019** | 0.97 (0.87 – 1.09) | 0.665 | 0.000 | 0% |
| **LoDoCo-MI, 2019** | 0.97 (0.87 – 1.09) | 0.665 | 0.000 | 0% |
| **LoDoCo2, 2020** | 0.97 (0.85 – 1.11) | 0.676 | 0.000 | 0% |
| **Australian COPS, 2020** | 0.97 (0.87 – 1.09) | 0.665 | 0.000 | 0% |
| **ASSAIL-MI, 2021** | 0.97 (0.87 – 1.09) | 0.638 | 0.000 | 0% |
| **Akrami et al., 2021** | 0.97 (0.87 – 1.09) | 0.665 | 0.000 | 0% |
| **CLEVER-ACS, 2022** | 0.97 (0.87 – 1.09) | 0.665 | 0.000 | 0% |
| **PodCAST-PCI, 2022** | 0.97 (0.87 – 1.09) | 0.665 | 0.000 | 0% |
| **COVERT-MI, 2023** | 0.97 (0.87 – 1.09) | 0.665 | 0.000 | 0% |
| **CLEAR SYERGY (OASIS 9), 2024** | 0.97 (0.87 – 1.09) | 0.665 | 0.000 | 0% |
| **Pneumonia** | | | | |
| **Pooled estimate** | **1.11 (0.74 – 1.66)** | **0.613** | **0.084** | **32%** |
| **COMPLY, 2003** | 1.11 (0.74 – 1.66) | 0.613 | 0.084 | 32% |
| **COMMA, 2003** | 1.11 (0.74 – 1.66) | 0.613 | 0.084 | 32% |
| **APEX AMI, 2007** | 1.11 (0.74 – 1.66) | 0.613 | 0.084 | 32% |
| **Piot et al., 2008** | 1.11 (0.74 – 1.66) | 0.613 | 0.084 | 32% |
| **Ghaffari et al., 2012** | 1.11 (0.74 – 1.66) | 0.613 | 0.084 | 32% |
| **VCU-ART2, 2013** | 1.14 (0.74 – 1.76) | 0.549 | 0.100 | 42% |
| **SELECT-ACS, 2013** | 1.11 (0.74 – 1.66) | 0.613 | 0.084 | 32% |
| **MRC-ILA, 2014** | 1.11 (0.74 – 1.66) | 0.613 | 0.084 | 32% |
| **CIRCUS, 2015** | 1.11 (0.74 – 1.66) | 0.613 | 0.084 | 32% |
| **CYCLE, 2015** | 1.11 (0.74 – 1.66) | 0.613 | 0.084 | 32% |
| **Deftereos et al., 2015** | 1.11 (0.74 – 1.66) | 0.613 | 0.084 | 32% |
| **COLIN, 2016** | 1.11 (0.74 – 1.66) | 0.613 | 0.084 | 32% |
| **Kleveland et al., 2016** | 1.11 (0.74 – 1.66) | 0.613 | 0.084 | 32% |
| **CANTOS, 2017** | 1.21 (0.55 – 2.65) | 0.639 | 0.297 | 46% |
| **STAT-MI, 2017** | 1.11 (0.74 – 1.66) | 0.613 | 0.084 | 32% |
| **TETHYS, 2017** | 1.14 (0.74 – 1.76) | 0.546 | 0.100 | 42% |
| **CIRT, 2018** | 1.11 (0.74 – 1.66) | 0.613 | 0.084 | 32% |
| **COLCOT, 2019** | 0.99 (0.81 – 1.21) | 0.954 | 0.001 | 0% |
| **VCUART3, 2019** | 1.11 (0.74 – 1.66) | 0.613 | 0.084 | 32% |
| **LoDoCo-MI, 2019** | 1.11 (0.74 – 1.66) | 0.613 | 0.084 | 32% |
| **LoDoCo2, 2020** | 1.32 (0.72 – 2.45) | 0.371 | 0.158 | 30% |
| **Australian COPS, 2020** | 1.08 (0.73 – 1.60) | 0.707 | 0.077 | 37% |
| **ASSAIL-MI, 2021** | 1.11 (0.74 – 1.66) | 0.613 | 0.084 | 32% |
| **Akrami et al., 2021** | 1.11 (0.74 – 1.66) | 0.613 | 0.084 | 32% |
| **CLEVER-ACS, 2022** | 1.11 (0.74 – 1.66) | 0.613 | 0.084 | 32% |
| **PodCAST-PCI, 2022** | 1.11 (0.74 – 1.66) | 0.613 | 0.084 | 32% |
| **COVERT-MI, 2023** | 1.11 (0.74 – 1.66) | 0.613 | 0.084 | 32% |
| **CLEAR SYERGY (OASIS 9), 2024** | 1.11 (0.74 – 1.66) | 0.613 | 0.084 | 32% |
| **Gastrointestinal adverse events** | | | | |
| **Pooled estimate** | **1.21 (1.07 – 1.36)** | **0.002** | **<0.001** | **31%** |
| **COMPLY, 2003** | 1.21 (1.07 – 1.36) | 0.002 | <0.001 | 31% |
| **COMMA, 2003** | 1.21 (1.07 – 1.36) | 0.002 | <0.001 | 31% |
| **APEX AMI, 2007** | 1.21 (1.07 – 1.36) | 0.002 | <0.001 | 31% |
| **Piot et al., 2008** | 1.21 (1.07 – 1.36) | 0.002 | <0.001 | 31% |
| **Ghaffari et al., 2012** | 1.21 (1.07 – 1.36) | 0.002 | <0.001 | 31% |
| **VCU-ART2, 2013** | 1.21 (1.07 – 1.36) | 0.002 | <0.001 | 31% |
| **SELECT-ACS, 2013** | 1.21 (1.07 – 1.36) | 0.002 | <0.001 | 31% |
| **MRC-ILA, 2014** | 1.21 (1.07 – 1.36) | 0.002 | <0.001 | 31% |
| **CIRCUS, 2015** | 1.21 (1.07 – 1.36) | 0.002 | <0.001 | 31% |
| **CYCLE, 2015** | 1.21 (1.07 – 1.36) | 0.002 | <0.001 | 31% |
| **Deftereos et al., 2015** | 1.20 (1.06 – 1.35) | 0.003 | 0.000 | 0% |
| **COLIN, 2016** | 1.20 (1.07 – 1.35) | 0.002 | <0.001 | 14% |
| **Kleveland et al., 2016** | 1.21 (1.07 – 1.36) | 0.002 | <0.001 | 31% |
| **CANTOS, 2017** | 1.21 (1.07 – 1.36) | 0.002 | <0.001 | 31% |
| **STAT-MI, 2017** | 1.21 (1.07 – 1.36) | 0.002 | <0.001 | 31% |
| **TETHYS, 2017** | 1.21 (1.08 – 1.37) | 0.002 | <0.001 | 38% |
| **CIRT, 2018** | 1.17 (0.98 – 1.41) | 0.082 | <0.001 | 38% |
| **COLCOT, 2019** | 1.20 (1.06 – 1.36) | 0.003 | <0.001 | 38% |
| **VCUART3, 2019** | 1.21 (1.07 – 1.36) | 0.002 | <0.001 | 31% |
| **LoDoCo-MI, 2019** | 1.20 (1.06 – 1.35) | 0.003 | <0.001 | 33% |
| **LoDoCo2, 2020** | 1.23 (1.08 – 1.39) | 0.001 | <0.001 | 36% |
| **Australian COPS, 2020** | 1.23 (1.08 – 1.40) | 0.002 | <0.001 | 37% |
| **ASSAIL-MI, 2021** | 1.21 (1.07 – 1.36) | 0.002 | <0.001 | 37% |
| **Akrami et al., 2021** | 1.21 (1.07 – 1.36) | 0.002 | <0.001 | 37% |
| **CLEVER-ACS, 2022** | 1.21 (1.07 – 1.36) | 0.002 | <0.001 | 31% |
| **PodCAST-PCI, 2022** | 1.21 (1.07 – 1.36) | 0.002 | <0.001 | 31% |
| **COVERT-MI, 2023** | 1.21 (1.07 – 1.36) | 0.002 | <0.001 | 31% |
| **CLEAR SYERGY (OASIS 9), 2024** | 1.21 (1.07 – 1.36) | 0.002 | <0.001 | 37% |

The table displays the recalculated IRRs for the pooled analysis after removing one trial at a time, in order to find major influences on the overall estimates and contribution to the overall heterogeneity. Pooled estimate is highlighted on top in light yellow cells. For each trial omitted, significant results are highlighted by a light-blue or light-red color in case the treatment reduced or increased the event rate, respectively.
Abbreviations: CI, confidence interval; IRR, incidence rate ratio; MACE, major adverse cardiovascular events.

**Supplementary Table 26. Meta-regression analysis.**

| **Subgroup** | **Estimate (95% CI)** | **p-value** | **R^2^** | **Residual I^2^** |
| --- | --- | --- | --- | --- |
| **MACE** | | | | |
| Year of publication | -0.01 (-0.02 to 0.02) | 0.115 | 100% | 0% |
| Age | 0.02 (-0.02 to 0.05) | 0.353 | 0% | 24% |
| Female | 0.00 (-0.02 to 0.01) | 0.727 | 0% | 10% |
| Diabetes | 0.00 (-0.01 to 0.01) | 0.906 | 0% | 11% |
| Hypertension | 0.00 (0.00 to 0.00) | 0.763 | 0% | 15% |
| Statins | 0.00 (-0.01 to 0.00) | 0.508 | 0% | 6% |
| Beta-blockers | 0.00 (0.00 to 0.01) | 0.541 | 7% | 17% |
| ACE-I or ARBs | 0.00 (-0.01 to 0.00) | 0.407 | 0% | 10% |
| Antiplatelet therapy | 0.00 (-0.01 to 0.00) | 0.519 | 0% | 27% |
| **Serious adverse events** | | | | |
| Year of publication | 0.00 (-0.02 to 0.02) | 0.656 | 0% | 0% |
| Age | 0.02 (-0.01 to 0.04) | 0.249 | 0% | 0% |
| Female | 0.00 (-0.02 to 0.01) | 0.814 | 0% | 0% |
| Diabetes | 0.00 (-0.01 to 0.01) | 0.543 | 0% | 0% |
| Hypertension | 0.02 (0.00 to 0.01) | 0.244 | 0% | 0% |
| Statins | 0.00 (-0.01 to 0.00) | 0.273 | 0% | 0% |
| Beta-blockers | 0.00 (-0.01 to 0.01) | 0.945 | 0% | 0% |
| ACE-I or ARBs | 0.00 (-0.01 to 0.01) | 0.649 | 0% | 0% |
| Antiplatelet therapy | -0.03 (-0.07 to 0.02) | 0.228 | 0% | 0% |
| **All-cause death** | | | | |
| Year of publication | 0.00 (-0.02 to 0.02) | 0.856 | 0% | 5% |
| Age | 0.04 (-0.01 to 0.08) | 0.103 | 100% | 0% |
| Female | 0.00 (-0.02 to 0.02) | 0.896 | 0% | 5% |
| Diabetes | 0.00 (-0.01 to 0.01) | 0.439 | 28% | 2% |
| Hypertension | 0.00 (-0.01 to 0.01) | 0.824 | 0% | 7% |
| Statins | 0.00 (0.00 to 0.01) | 0.562 | 0% | 17% |
| Beta-blockers | -0.02 (-0.05 to 0.01) | 0.131 | 0% | 0% |
| ACE-I or ARBs | 0.00 (-0.06 to 0.01) | 0.468 | 0% | 18% |
| Antiplatelet therapy | 0.00 (0.00 to 0.01) | 0.413 | 0% | 7% |
| **Cardiovascular death** | | | | |
| Year of publication | 0.00 (-0.03 to 0.06) | 0.512 | 0% | 0% |
| Age | 0.01 (-0.06 to 0.08) | 0.781 | 0% | 1% |
| Female | -0.02 (-0.05 to 0.02) | 0.373 | 0% | 0% |
| Diabetes | -0.01 (-0.02 to 0.01) | 0.308 | 0% | 0% |
| Hypertension | 0.00 (-0.01 to 0.01) | 0.527 | 0% | 0% |
| Statins | 0.00 (-0.04 to 0.04) | 0.892 | 0% | 7% |
| Beta-blockers | 0.00 (-0.03 to 0.03) | 0.992 | 0% | 0% |
| ACE-I or ARBs | -0.02 (-0.06 to 0.02) | 0.377 | 100% | 0% |
| Antiplatelet therapy | 0.00 (-0.08 to 0.09) | 0.917 | 0% | 0% |
| **Myocardial infarction** | | | | |
| Year of publication | -0.02 (-0.04 to -0.00) | 0.088 | 75% | 3% |
| Age | -0.01 (-0.06 to 0.05) | 0.770 | 0% | 16% |
| Female | 0.03 (-0.01 to 0.07) | 0.171 | 0% | 24% |
| Diabetes | 0.00 (-0.01 to 0.01) | 0.939 | 0% | 18% |
| Hypertension | 0.00 (0.00 to 0.01) | 0.440 | 100% | 0% |
| Statins | 0.00 (-0.01 to 0.01) | 0.640 | 0% | 18% |
| Beta-blockers | 0.01 (0.01 to 0.02) | 0.035 | 88% | 4% |
| ACE-I or ARBs | 0.01 (-0.01 to 0.03) | 0.362 | 0% | 30% |
| Antiplatelet therapy | 0.00 (-0.01 to 0.01) | 0.970 | 0% | 24% |
| **Heart failure** | | | | |
| Year of publication | -0.01 (-0.03 to 0.02) | 0.588 | 0% | 0% |
| Age | 0.00 (-0.08 to 0.07) | 0.919 | 0% | 0% |
| Female | -0.01 (-0.04 to 0.02) | 0.506 | 0% | 0% |
| Diabetes | 0.00 (-0.02 to 0.01) | 0.784 | 0% | 0% |
| Hypertension | 0.00 (-0.01 to 0.01) | 0.699 | 0% | 0% |
| Statins | 0.00 (-0.01 to 0.01) | 0.801 | 0% | 0% |
| Beta-blockers | 0.00 (0.01 to 0.01) | 0.806 | 0% | 0% |
| ACE-I or ARBs | 0.01 (-0.01 to 0.01) | 0.639 | 0% | 0% |
| Antiplatelet therapy | 0.00 (-0.01 to 0.01) | 0.781 | 0% | 0% |
| **Revascularization** | | | | |
| Year of publication | -0.02 (-0.05 to 0.02) | 0.348 | 57% | 0% |
| Age | -0.02 (-0.10 to 0.07) | 0.658 | 0% | 0% |
| Female | 0.00 (-0.04 to 0.04) | 0.997 | 0% | 60% |
| Diabetes | -0.01 (-0.03 to 0.00) | 0.143 | 43% | 39% |
| Hypertension | -0.01 (-0.01 to 0.00) | 0.105 | 56% | 32% |
| Statins | 0.00 (-0.01 to 0.01) | 0.452 | 0% | 61% |
| Beta-blockers | 0.00 (-0.02 to 0.02) | 0.817 | 0% | 67% |
| ACE-I or ARBs | 0.00 (-0.02 to 0.01) | 0.803 | 0% | 55% |
| Antiplatelet therapy | 0.00 (-0.01 to 0.06) | 0.522 | 0% | 68% |
| **Stroke** | | | | |
| Year of publication | 0.00 (-0.04 to 0.04) | 0.964 | 0% | 17% |
| Age | 0.01 (-0.01 to 0.11) | 0.924 | 0% | 21% |
| Female | 0.03 (-0.01 to 0.08) | 0.170 | 0% | 11% |
| Diabetes | 0.00 (-0.02 to 0.03) | 0.568 | 0% | 23% |
| Hypertension | 0.01 (-0.01 to 0.02) | 0.460 | 0% | 23% |
| Statins | 0.01 (-0.01 to 0.02) | 0.439 | 0% | 0% |
| Beta-blockers | 0.01 (-0.01 to 0.03) | 0.385 | 22% | 25% |
| ACE-I or ARBs | 0.01 (-0.01 to 0.03) | 0.308 | 0% | 0% |
| Antiplatelet therapy | 0.00 (-0.02 to 0.02) | 0.851 | 0% | 41% |
| **Serious infection or sepsis** | | | | |
| Year of publication | 0.00 (-0.02 to 0.03) | 0.732 | 0% | 8% |
| Age | -0.02 (-0.06 to 0.03) | 0.461 | 34% | 3% |
| Female | 0.02 (-0.01 to 0.04) | 0.134 | 100% | 0% |
| Diabetes | 0.01 (0.00 to 0.02) | 0.132 | 100% | 0% |
| Hypertension | 0.00 (0.00 to 0.01) | 0.471 | 20% | 4% |
| Statins | 0.00 (-0.01 to 0.01) | 0.512 | 0% | 6% |
| Beta-blockers | 0.00 (-0.01 to 0.01) | 0.815 | 0% | 0% |
| ACE-I or ARBs | 0.01 (-0.01 to 0.02) | 0.801 | 85% | 1% |
| Antiplatelet therapy | 0.00 (-0.01 to 0.01) | 0.529 | 0% | 0% |

Abbreviations: ACE-i, angiotensin converting enzyme inhibitor; ARB, angiotensin receptor blockers.

**SUPPLEMENTARY FIGURES**

**Supplementary Figure 1. Systematic research flow.**

The figure illustrates the level at which each study was excluded during the review process.

Abbreviations: N, number.

**Supplementary Figure 2. Network of treatment.**

The figure illustrates the network of treatments for the indirect comparison of anti-inflammatory drugs. Circles are proportional to the number of patients receiving the treatment, while lines are proportional to the number of trials comparing the treatments.

Abbreviations: N, number of patients; P-Year, patient-year.

**Supplementary Figure 3. Overall risk of bias.**

Abbreviations: RoB, risk of bias.

**Supplementary Figure 4. Individual risk of bias across the included studies.**

**Supplementary Figure 5. Funnel plots and Egger’s test for publication bias.**

The plot shows funnel plots for each prespecified endpoints. Light gray zones represent zones of 99% confidence interval for publication bias, while dark gray zones represent zones of 95% confidence interval for publication bias. Results of the Egger tests are shown in the top left corner for each endpoint.
Abbreviations: MACE, major adverse cardiovascular events; P-Egger: p-value for the Egger’s regression test; SAE, serious adverse events.

**Supplementary Figure 6. Influence analysis for MACE.**

The plot shows assessment of the contrast between individual trial influence on results (left) and heterogeneity for the endpoint (bottom). Trials on the top right corners have the highest influence on both treatment effect estimate and heterogeneity, while trials in the bottom left corner have the lower influence on both. Circles are proportional to the trial’s weight on the pooled effect-size estimation.
Abbreviations: MACE, major adverse cardiovascular events

**Supplementary** **Figure 7. Forest plot and interaction analysis for MACE after excluding MRC-ILA, STAT-MI and Akramy et al.**

The plot exemplifies treatment effect estimates for anti-inflammatory therapy and each subgroup. The pooled effect is displayed as a diamond shape, while single trial’s effect and relative confidence intervals are displayed as squares and bar, respectively. For each subgroup, specific pooled estimate is displayed along with heterogeneity estimates and p-value for effect within the subgroup. Overall pooled estimate is displayed at the bottom, along with heterogeneity estimates and results of the test for between-subgroup interaction.

**Supplementary Figure 8. Forest plot and interaction analysis for all-cause death.**

The plot exemplifies treatment effect estimates for anti-inflammatory therapy and each subgroup. The pooled effect is displayed as a diamond shape, while single trial’s effect and relative confidence intervals are displayed as squares and bar, respectively. For each subgroup, specific pooled estimate is displayed along with heterogeneity estimates and p-value for effect within the subgroup. Overall pooled estimate is displayed at the bottom, along with heterogeneity estimates and results of the test for between-subgroup interaction.

**Supplementary Figure 9. Forest plot and interaction analysis for cardiovascular death.**

The plot exemplifies treatment effect estimates for anti-inflammatory therapy and each subgroup. The pooled effect is displayed as a diamond shape, while single trial’s effect and relative confidence intervals are displayed as squares and bar, respectively. For each subgroup, specific pooled estimate is displayed along with heterogeneity estimates and p-value for effect within the subgroup. Overall pooled estimate is displayed at the bottom, along with heterogeneity estimates and results of the test for between-subgroup interaction.**Supplementary Figure 10. Forest plot and interaction analysis for myocardial infarction.**

The plot exemplifies treatment effect estimates for anti-inflammatory therapy and each subgroup. The pooled effect is displayed as a diamond shape, while single trial’s effect and relative confidence intervals are displayed as squares and bar, respectively. For each subgroup, specific pooled estimate is displayed along with heterogeneity estimates and p-value for effect within the subgroup. Overall pooled estimate is displayed at the bottom, along with heterogeneity estimates and results of the test for between-subgroup interaction.

**Supplementary Figure 11. Forest plot and interaction analysis for heart failure.**

The plot exemplifies treatment effect estimates for anti-inflammatory therapy and each subgroup. The pooled effect is displayed as a diamond shape, while single trial’s effect and relative confidence intervals are displayed as squares and bar, respectively. For each subgroup, specific pooled estimate is displayed along with heterogeneity estimates and p-value for effect within the subgroup. Overall pooled estimate is displayed at the bottom, along with heterogeneity estimates and results of the test for between-subgroup interaction.

**Supplementary Figure 12. Forest plot and interaction analysis for revascularization.**

The plot exemplifies treatment effect estimates for anti-inflammatory therapy and each subgroup. The pooled effect is displayed as a diamond shape, while single trial’s effect and relative confidence intervals are displayed as squares and bar, respectively. For each subgroup, specific pooled estimate is displayed along with heterogeneity estimates and p-value for effect within the subgroup. Overall pooled estimate is displayed at the bottom, along with heterogeneity estimates and results of the test for between-subgroup interaction.

**Supplementary Figure 13. Forest plot and interaction analysis for stroke.**

The plot exemplifies treatment effect estimates for anti-inflammatory therapy and each subgroup. The pooled effect is displayed as a diamond shape, while single trial’s effect and relative confidence intervals are displayed as squares and bar, respectively. For each subgroup, specific pooled estimate is displayed along with heterogeneity estimates and p-value for effect within the subgroup. Overall pooled estimate is displayed at the bottom, along with heterogeneity estimates and results of the test for between-subgroup interaction.

**Supplementary Figure 14. Forest plot and interaction analysis for serious infection or sepsis.**

The plot exemplifies treatment effect estimates for anti-inflammatory therapy and each subgroup. The pooled effect is displayed as a diamond shape, while single trial’s effect and relative confidence intervals are displayed as squares and bar, respectively. For each subgroup, specific pooled estimate is displayed along with heterogeneity estimates and p-value for effect within the subgroup. Overall pooled estimate is displayed at the bottom, along with heterogeneity estimates and results of the test for between-subgroup interaction.

**Supplementary Figure 15. Forest plot and interaction analysis for cancer development.**

The plot exemplifies treatment effect estimates for anti-inflammatory therapy and each subgroup. The pooled effect is displayed as a diamond shape, while single trial’s effect and relative confidence intervals are displayed as squares and bar, respectively. For each subgroup, specific pooled estimate is displayed along with heterogeneity estimates and p-value for effect within the subgroup. Overall pooled estimate is displayed at the bottom, along with heterogeneity estimates and results of the test for between-subgroup interaction.

**Supplementary Figure 16. Forest plot and interaction analysis for pneumonia.**

The plot exemplifies treatment effect estimates for anti-inflammatory therapy and each subgroup. The pooled effect is displayed as a diamond shape, while single trial’s effect and relative confidence intervals are displayed as squares and bar, respectively. For each subgroup, specific pooled estimate is displayed along with heterogeneity estimates and p-value for effect within the subgroup. Overall pooled estimate is displayed at the bottom, along with heterogeneity estimates and results of the test for between-subgroup interaction.

**Supplementary Figure 17. Forest plot and interaction analysis for gastrointestinal adverse events.**

The plot exemplifies treatment effect estimates for anti-inflammatory therapy and each subgroup. The pooled effect is displayed as a diamond shape, while single trial’s effect and relative confidence intervals are displayed as squares and bar, respectively. For each subgroup, specific pooled estimate is displayed along with heterogeneity estimates and p-value for effect within the subgroup. Overall pooled estimate is displayed at the bottom, along with heterogeneity estimates and results of the test for between-subgroup interaction.

**Supplementary Figure 18. Influence analysis for revascularization.**

The plot shows assessment of the contrast between individual trial influence on results (left) and heterogeneity for the endpoint (bottom). Trials on the top right corners have the highest influence on both treatment effect estimate and heterogeneity, while trials in the bottom left corner have the lower influence on both. Circles are proportional to the trial’s weight on the pooled effect-size estimation.

**Supplementary Figure 19. Forest plot and interaction analysis for revascularization after excluding Australian COPS, MRC-ILA, SELECT-ACS, COLCOT and CLEAR SYNERGY (OASIS9) trials.**

The plot exemplifies treatment effect estimates for anti-inflammatory therapy and each subgroup. The pooled effect is displayed as a diamond shape, while single trial’s effect and relative confidence intervals are displayed as squares and bar, respectively. For each subgroup, specific pooled estimate is displayed along with heterogeneity estimates and p-value for effect within the subgroup. Overall pooled estimate is displayed at the bottom, along with heterogeneity estimates and results of the test for between-subgroup interaction.

**Supplementary Figure 20. Influence analysis for revascularization within the colchicine subgroup.**

The plot shows assessment of the contrast between individual trial influence on results (left) and heterogeneity for the endpoint (bottom). Trials on the top right corners have the highest influence on both treatment effect estimate and heterogeneity, while trials in the bottom left corner have the lower influence on both. Circles are proportional to the trial’s weight on the pooled effect-size estimation.

**Supplementary Figure 21. Forest plot for revascularization within the colchicine subgroup after excluding LoDoCo2 trial.**

The plot exemplifies treatment effect estimates for colchicine against placebo. The pooled effect is displayed at the bottom as a diamond shape, along with heterogeneity measures, while single trial’s effect and relative confidence intervals are displayed as squares and bar, respectively.

**Supplementary Figure 22. Influence analysis for pneumonia.**

The plot shows assessment of the contrast between individual trial influence on results (left) and heterogeneity for the endpoint (bottom). Trials on the top right corners have the highest influence on both treatment effect estimate and heterogeneity, while trials in the bottom left corner have the lower influence on both. Circles are proportional to the trial’s weight on the pooled effect-size estimation.

**Supplementary Figure 23. Influence analysis for gastrointestinal adverse events.**

The plot shows assessment of the contrast between individual trial influence on results (left) and heterogeneity for the endpoint (bottom). Trials on the top right corners have the highest influence on both treatment effect estimate and heterogeneity, while trials in the bottom left corner have the lower influence on both. Circles are proportional to the trial’s weight on the pooled effect-size estimation.

**Supplementary Figure 24. Forest plot and interaction analysis for pneumonia after removal of COLCOT trial.**

The plot exemplifies treatment effect estimates for anti-inflammatory therapy and each subgroup. The pooled effect is displayed as a diamond shape, while single trial’s effect and relative confidence intervals are displayed as squares and bar, respectively. For each subgroup, specific pooled estimate is displayed along with heterogeneity estimates and p-value for effect within the subgroup. Overall pooled estimate is displayed at the bottom, along with heterogeneity estimates and results of the test for between-subgroup interaction.

**Supplementary Figure 25. Forest plot and interaction analysis for gastrointestinal adverse events after removal of Deftereos et al. trial.**

The plot exemplifies treatment effect estimates for anti-inflammatory therapy and each subgroup. The pooled effect is displayed as a diamond shape, while single trial’s effect and relative confidence intervals are displayed as squares and bar, respectively. For each subgroup, specific pooled estimate is displayed along with heterogeneity estimates and p-value for effect within the subgroup. Overall pooled estimate is displayed at the bottom, along with heterogeneity estimates and results of the test for between-subgroup interaction.
